# Developing precision nanoparticle-based drug delivery systems for Alzheimer's disease therapy

**DOI:** 10.1016/j.mtbio.2026.103789

**Published:** 2026-10-27

**Authors:** Qiujie Shan, Wenao Zheng, Weina Yao, Yamei Bai, Wanchuan Ding, Ji Wang, Feng Bai

**Affiliations:** a Department of Neurology, Nanjing Drum Tower Hospital, Affiliated Hospital of Medical School, Nanjing University, Nanjing, 210008, China; b Department of Neurology, Zhongnan Hospital of Wuhan University, Wuhan, 430071, China; c School of Nursing, Nanjing University of Chinese Medicine, Nanjing, 210023, China; d Department of Rheumatology and Immunology, Nanjing Drum Tower Hospital, Nanjing, 210008, China

**Keywords:** Alzheimer's disease, Nanoparticles, Precision drug delivery, Disease-modifying therapy, Blood-brain barrier, Nanotechnology

## Abstract

Alzheimer's disease (AD) is an irreversible neurodegenerative disease that has thus far suffered from unsatisfactory therapeutic outcomes. Despite ongoing research into AD pathogenesis and related drugs, few disease-modifying therapies are currently available. Moreover, efficient drug delivery is significantly hindered by low permeability of the blood-brain barrier (BBB) and non-specific distribution within the brain, further limiting the efficacy of existing anti-AD medications. Nanotechnology has recently emerged as a promising approach to overcome these challenges. This review aims to offer a comprehensive overview of recent advances in precision nanoparticle-based drug delivery systems for AD therapy. First, we outline the multiple pathological mechanisms and representative pathogenesis-inspired therapies of AD. Next, we summarize various types of nanoparticles that have been utilized in AD drug delivery, including polymeric, lipid-based, inorganic, and biomimetic nanocarriers. Subsequently, we highlight the nanoparticle-mediated strategies for cascaded precision drug delivery at different levels, focusing on both the BBB and specific pathologies or cell populations within AD lesions. Finally, we discuss potential challenges and prospects in employing precision delivery systems, aiming to accelerate the development and clinical translation of nanoparticle-based therapies for AD.

## Graphical abstract

**Figure:**
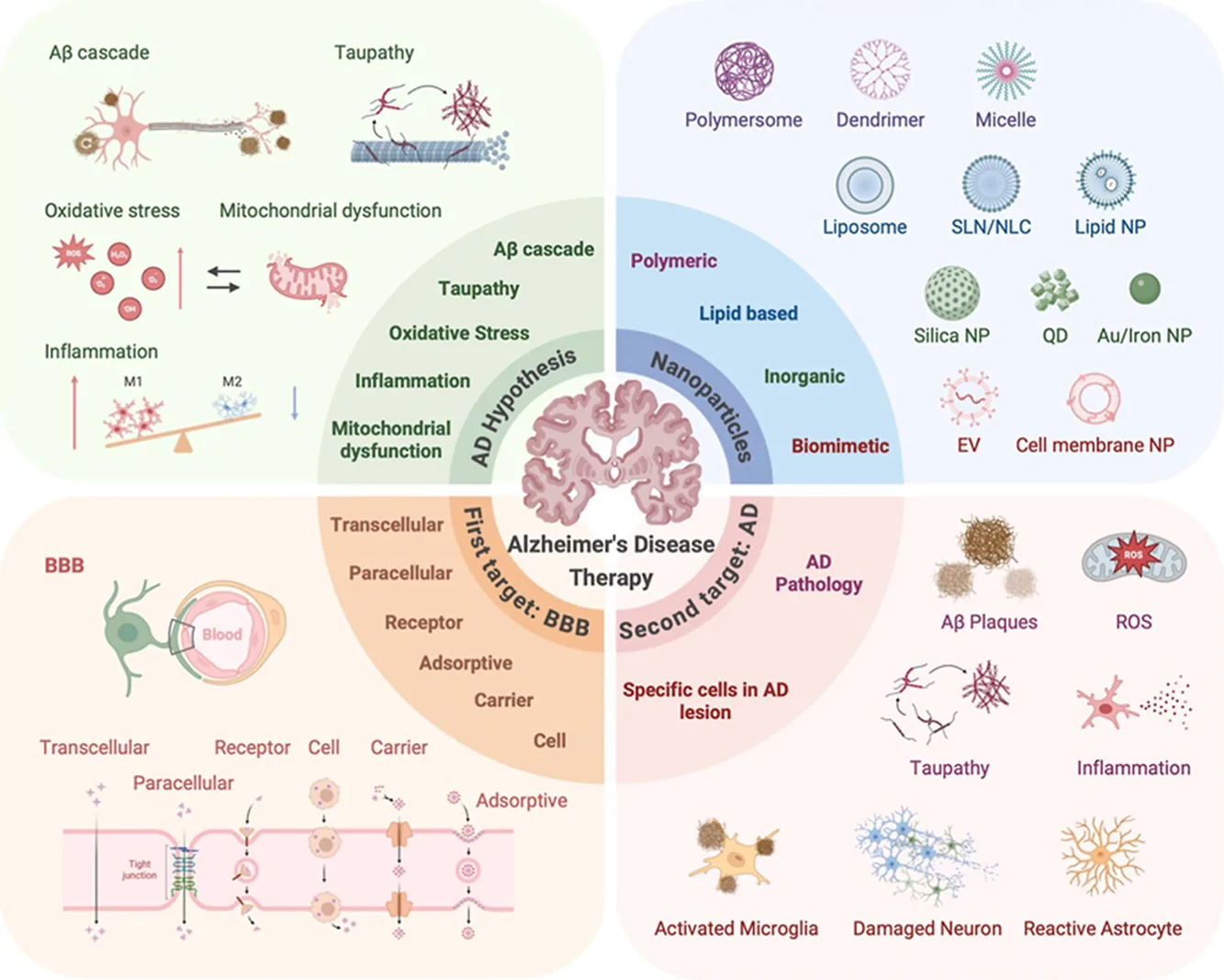

## 1 Introduction

Alzheimer's disease (AD) is a chronic neurodegenerative disease featuring progressive deterioration in cognitive functions and daily living abilities [1]. Globally, an estimated 55.2 million people suffer from dementia, of which AD accounts for 60-80% of these cases [2]. With worldwide life expectancy increasing and the population aging process accelerating, this number is predicted to triple by 2050, which will impose tremendous economic and mental burdens on affected individuals and society [3]. Multiple hypotheses of AD pathogenesis have been proposed, such as β-amyloid (Aβ) deposition, tau hyperphosphorylation, neuroinflammation, oxidative stress, and mitochondrial dysfunction. However, it is unrealistic to fully clarify the particular mechanism by a single pathological process [4]. Instead, multiple pathological factors interact with each other and synergistically promote disease progression and aggravation, thus making the treatment particularly complicated and challenging. To date, the U.S. Food and Drug Administration (FDA) has approved nine drugs for AD treatment in total, including tacrine, donepezil, rivastigmine, galantamine, memantine, namzaric, aducanumab, lecanemab, and donanemab [5], [6], [7], [8]. Notably, tacrine was withdrawn from the market due to severe hepatotoxicity. Conventional drugs, including acetylcholinesterase inhibitors (AChEIs) and N-methyl-D-aspartate receptor antagonists, can merely temporarily alleviate AD symptoms but fail to reverse or even retard disease progression [9]. In this context, disease-modifying therapy (DMT) has been proposed, aiming to intervene in the underlying pathophysiology of AD and alter the trajectory of disease progression [10]. Aducanumab, lecanemab, and donanemab are three recently approved Aβ-targeting monoclonal antibodies that aim to slow down cognitive and functional deterioration in patients by promoting cerebral Aβ clearance, representing the latest promise in DMT for AD [11]. Unfortunately, the development and marketing of aducanumab have ceased due to insufficient evidence of its clinical benefits [12]. Overall, despite enormous time and financial expenses invested over the last decades, the therapeutic efficacy of current anti-AD medications remains unsatisfactory.

In addition, another significant challenge in effective AD treatment is the blood-brain barrier (BBB). Physiologically, the BBB is a dynamic and selectively permeable membrane made up of brain microvascular endothelial cells (BMECs), astrocytes, pericytes, and tight junctions, playing a vital role in regulating the exchange of substances across the brain and maintaining the homeostasis of the central nervous system (CNS). As the most strictly controlled interface *in vivo*, the BBB serves as a protective barrier that isolates toxins, pathogens, and other detrimental substances in the bloodstream from the CNS. Yet, this protective membrane also impedes the entry of almost all the macromolecular drugs and 98% of small-molecular drugs into the brain, posing a great challenge for achieving effective drug accumulation in the brain parenchyma [13]. Despite BBB dysfunction and increased BBB permeability being reported in AD, drug delivery strategies cannot overly rely on this uncertain leakage due to the vast heterogeneity among AD patients [14], [15]. Furthermore, when drugs do cross the BBB and enter the CNS, non-specific distribution within the brain parenchyma, as opposed to precisely targeting the AD lesions, not only compromises therapeutic efficacy but also aggravates adverse effects due to unexpected drug accumulation in other brain regions [16]. Therefore, there is an urgent need to develop effective and safe precision drug delivery strategies that facilitate crossing the BBB while specifically targeting AD lesions.

Advanced nanotechnology has recently emerged, showing great promise in overcoming the aforementioned challenges. Diverse classes of nanoparticles, including polymeric, lipid-based, inorganic, and biomimetic nanoparticles, have been widely exploited as drug-delivery carriers because of their flexible size, customizable architecture, tunable surface charge, desirable stability, and good biocompatibility and biodegradability [17], [18]. Additionally, traditional single-target medications have proven inadequate for addressing the complicated neuropathology of AD. Fortunately, due to their high drug-loading capacity, nanoparticles enable the co-delivery of multiple drugs to the brain within a single formulation, providing a feasible platform for multi-target combination therapy for AD. Notably, some nanomaterials have the intrinsic potential to intervene in AD progression, such as Aβ depolymerization, tau clearance, reactive oxygen species (ROS) scavenging, metal ion chelation, and inflammation suppression, functioning not only as drug delivery carriers but also as novel therapeutic agents for AD [19], [20]. More importantly, these nanoparticles can be engineered through diverse surface modifications and functionalization techniques to facilitate their crossing of the BBB and targeting of neuropathology-specific or cell-specific sites, thereby providing more opportunities for precision delivery and enhanced drug accumulation in AD lesions [21].

In this review, we seek to provide a comprehensive overview of recent advances in precision nanoparticle-based drug delivery systems for AD therapy (Fig. 1). Here, we define precision drug delivery as a disease-guided and lesion-homing delivery paradigm that integrates the optimization of BBB penetration, selective accumulation at AD-relevant pathological sites, preferential engagement of defined brain cell populations, and, where applicable, stimulus-responsive drug release within the disease microenvironment. Whereas conventional brain-targeted nanomedicine has largely focused on enhancing BBB transport and overall brain accumulation, our review highlights their subsequent distribution after entering the brain, aiming to increase drug availability at the intended site while minimizing off-target exposure. Within this conceptual framework, we first discuss the multiple pathological mechanisms of AD and therapies inspired by related pathogenesis. Next, we systematically outline the various classes of nanoparticles that have been utilized in AD treatment. Subsequently, we focus on nanotechnology-mediated approaches to enhance their ability to cross the BBB. Moreover, we also explore versatile strategies for achieving precision targeting of specific pathologies, such as Aβ, tau, and neuroinflammation, as well as AD-relevant cell populations, including activated microglia, damaged neurons, and reactive astrocytes. Finally, we discuss the potential challenges and directions for future research that prospectively advance the clinical translatability of nanoparticle-based therapies for AD.

**Fig. 1:**
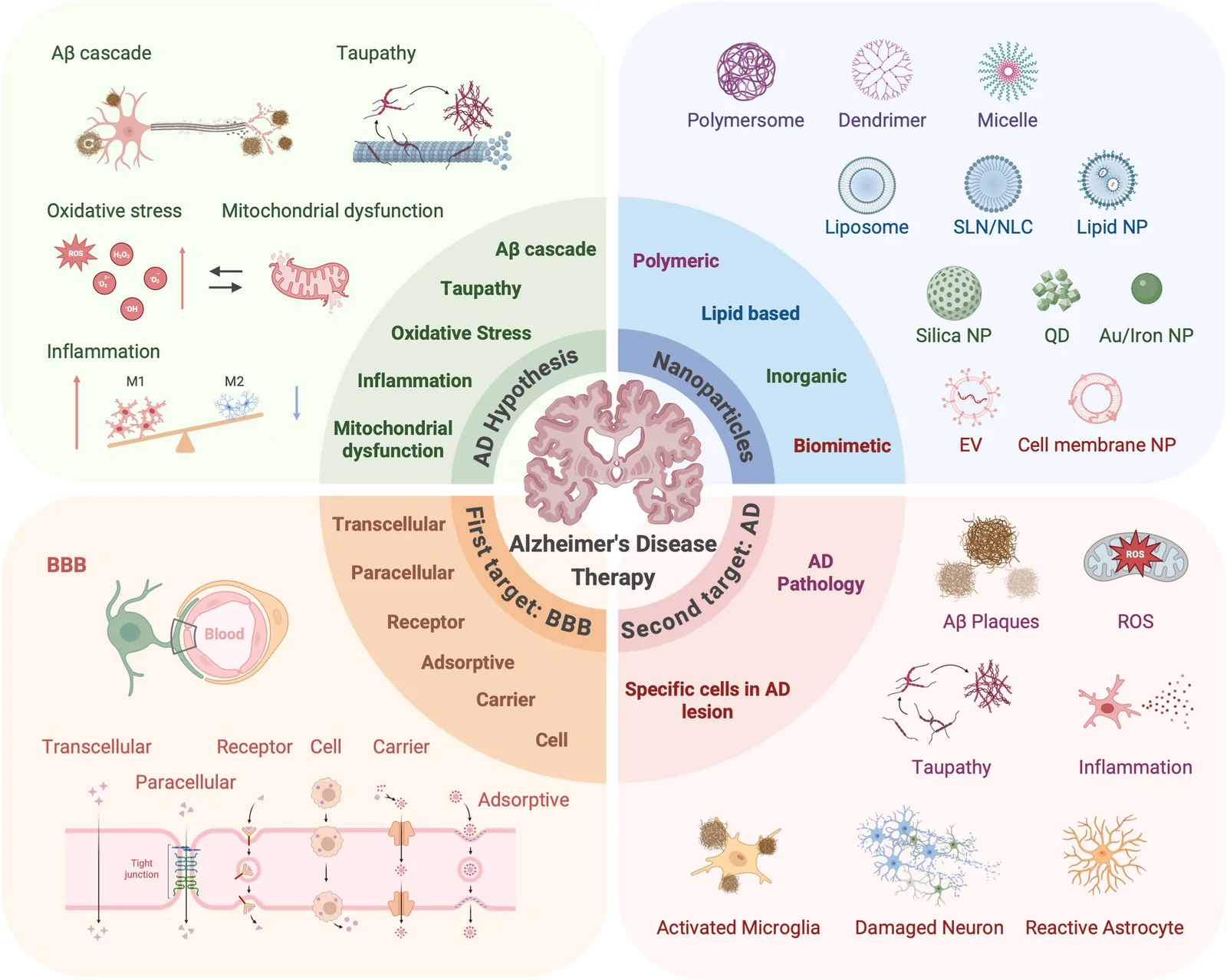
**Schematic illustration of precision nanoparticle-based drug delivery systems for ADtherapy.** Created in BioRender. Shan, J. (2026) https://BioRender.com/hi2dqjb.

## 2 AD pathogenesis and related targeting therapies

### 2.1 Aβ cascade hypothesis and Aβ-targeting therapies

The Aβ cascade hypothesis is considered the predominant hypothesis of AD pathogenesis. Aβ is generated from the abnormal degradation of amyloid precursor protein (APP). Physiologically, APP is sequentially cleaved by α-secretase and γ-secretase through the non-amyloidogenic pathway, producing the soluble extracellular fragment sAPPα that has neuroprotective effects [22]. However, under AD pathology, β-secretase (β-APP-cleaving enzyme-1, BACE1) primarily cleaves APP at the Asp1 site, leading to the generation of secreted sAPPβ and membrane-bound fragment C99, the latter of which can be subsequently cleaved into Aβ monomers of different lengths by γ-secretase [23]. These monomers, particularly the highly hydrophobic Aβ_1-42_, are prone to self-aggregate into various species, including Aβ oligomers, Aβ protofibrils, Aβ fibrils, and Aβ plaques, which exhibit great neurotoxicity and contribute to synaptic dysfunction and neuronal damage. Moreover, the Aβ cascade hypothesis suggests that excessive accumulation of Aβ aggregates (especially soluble Aβ oligomers) also triggers other AD pathological features, such as tau hyperphosphorylation, oxidative stress, neuroinflammatory responses, and mitochondrial dysfunction, collectively contributing to irreversible neuronal death and AD progression [24].

Aβ-targeted therapies can be mainly divided into three categories: (1) to reduce Aβ production; (2) to inhibit Aβ aggregation into forms with higher neurotoxicity; (3) to enhance Aβ clearance [25]. Aβ is generated by the aberrant sequential cleavage of APP by β-secretase and γ-secretase; thus, inhibitors targeting these two enzymes are widely investigated. Many candidates have entered clinical trials, including LY2886721, Atabecestat, Lanabecestat, and Umibecestat. Unfortunately, none of them succeeded in reaching the market due to off-target effects, adverse reactions, and lack of efficacy. In addition, the α-secretase modulator is another potential target to reduce the Aβ generation. MIB-626 is a proprietary formulation of the crystalline form of β-nicotinamide mononucleotide and can enhance α-secretase activity in AD patients, which is now being tested in a phase 2 trial. Preventing the self-assembly of Aβ monomers into more toxic Aβ aggregates is an effective strategy for AD therapy. Related drugs, including various antibodies, small molecules, chelating agents, and peptides, are continuously developed and studied. However, most applications are significantly restricted due to their low BBB permeability, poor bioavailability, and undesirable stability. Aβ immunotherapy has been recently recognized as the most promising strategy for Aβ clearance [26]. Aducanumab was the first FDA-approved Aβ-targeting humanized immunoglobulin gamma (IgG) 1 monoclonal antibody, directly binding to Aβ aggregates and facilitating cerebral Aβ clearance via microglia-mediated phagocytosis of the Aβ-antibody complex [6]. However, due to insufficient evidence of its clinical benefit, the development and sale of Aducanumab have ceased [12]. Two other IgG1 monoclonal antibodies, Lecanemab and Donanemab, obtained accelerated approval by the FDA on January 6, 2023, and July 2, 2024, respectively, bringing a new dawn to slow disease progression of early AD and delay dementia [7], [8].

From a drug-delivery perspective, the heterogeneous distribution of Aβ-related therapeutic targets demands different levels of delivery precision. Aβ antibodies, aggregation inhibitors, and Aβ-clearing agents, which target extracellular Aβ species, require efficient BBB penetration as well as preferential retention in Aβ-rich regions to increase their local effective concentration. In contrast, strategies aimed at reducing Aβ production by modulating APP-processing enzymes, such as BACE1, necessitate efficient neuronal uptake and access to intracellular processing compartments. Thus, precision delivery for Aβ-targeting therapies should be tailored to the specific therapeutic mechanism, ranging from BBB crossing and extracellular Aβ recognition to cellular internalization.

### 2.2 Tau hyperphosphorylation hypothesis and tau-targeting therapies

Tauopathy is another major pathological hallmark of AD. Tau is a microtubule-associated protein predominantly expressed in axons, where it binds to the microtubules and facilitates their assembly, playing a crucial role in regulating microtubule stability and maintaining neuronal function [27]. Under AD pathology, tau protein is abnormally hyperphosphorylated at multiple residues, which reduces its affinity to microtubules and impairs microtubule stability, consequently leading to microtubule depolymerization and axonal transport disruption [28]. Moreover, hyperphosphorylated tau is prone to self-aggregate into paired helical filaments (PHFs) and subsequently form neurofibrillary tangles (NFTs), resulting in synaptic loss, neuronal dysfunction, and cell death [29]. Growing evidence shows that toxic tau can be released from pathological neurons, propagate as a prion-like transferring pattern, and finally spread to various brain regions [30]. Of note, compared to aberrant Aβ deposition, tau hyperphosphorylation correlates more directly with concurrent cognitive impairment, indicating its determinant role in driving AD progression [31].

Tau-targeting therapeutic strategies encompass the reduction of tau production, inhibition of tau hyperphosphorylation and aggregation, enhancement of tau clearance, and stabilization of microtubules. Tau antisense oligonucleotides (ASOs), including BIIB080 and NIO752, have been developed to reduce tau expression by targeting human *MAPT* mRNA, which are now being tested in phase 2 and phase 1 trials, respectively [32]. Tau phosphorylation is primarily regulated by kinases and phosphatases; thus, relevant therapeutics primarily involve kinase inhibitors and phosphatase activators such as Leucettinib-21 and PP2A [33], [34]. However, these agents lack targeting specificity for pathological tau and exhibit a global inhibition of phosphorylation *in vivo*, which might lead to potential off-target effects in AD patients. LMTX (TRx0237) is a small-molecule derivative of methylene blue that can block tau aggregation and degrade existing aggregates and has shown therapeutic effects in AD animal models. However, no positive results have been found in clinical trials [35]. TPI-287 and NAP peptides (davunetide) are used as microtubule stabilizers, which can compensate for the loss of tau function, improving axonal transport and preventing neuronal degeneration [36], [37]. Currently, tau immunotherapy is a rapidly developing field, including active vaccines and anti-tau antibodies. ACI-35 is a liposome-based vaccine containing tau fragment phosphorylated at the residues S396 and S404, demonstrating acceptable safety, tolerability, and immunogenicity in clinical trials [38]. Anti-tau antibodies, such as E2814, Bepranemab, BIIB076, JNJ-63733657, APNmAb005, and MK-2214, can specifically target pathological tau, promote its intracellular degradation, or facilitate its extracellular clearance [28]. E2814 is a monoclonal IgG1 antibody that can recognize an HVPGG epitope in the microtubule-binding domain, which is a predominant component of NFTs and is involved in seeding and spreading pathological tau aggregates [39]. Two phase 3 trials are evaluating the safety, tolerability, and functional efficacy of E2814 alone or concurrently with anti-Aβ antibody lecanemab.

Precision delivery strategy for tau-directed therapeutics may depend on whether the intervention aims to suppress intracellular tau production, hyperphosphorylation, and aggregation or to intercept extracellular tau spreading. For most tau-targeting agents, such as tau ASOs, kinase inhibitors, and phosphatase activators, BBB penetration alone is insufficient, as tau-related pathology, including hyperphosphorylated tau, PHFs, and NFTs, is located intracellularly within vulnerable neurons. Thus, effective precision delivery requires additionally preferential localization to affected brain regions, neuron-selective uptake, efficient endosomal escape, and cytosolic release. Meanwhile, extracellular tau species are involved in trans-neuronal seeding and propagation, providing an alternative accessible target for antibodies or extracellular tau-clearing agents.

### 2.3 Neuroinflammation hypothesis and targeting therapies

Accumulating evidence demonstrates a pivotal role of neuroinflammation in the pathogenesis of AD, which is primarily associated with the overactivation of microglia and astrocytes [40]. AD pathology products, such as Aβ plaques and NFTs, serve as damage-associated molecular patterns and bind to relevant receptors on glial cells, activating the surrounding glial cells and leading to the generation of immune mediators and the recruitment of more glial cells to the AD lesion [41]. In the early stage of AD, activated glial cells can recognize and phagocytize the aggregated Aβ, thereby exerting neuroprotective functions on the brain. However, as the disease progresses, sustained activated glial cells release a variety of pro-inflammatory mediators and neurotoxins, including cytokines, chemokines, ROS, prostaglandins, and nitric oxide, amplifying immune responses and exacerbating neuronal damage [42]. Moreover, activated microglia also interact with other AD pathological processes, such as Aβ generation and aggregation, tau seeding and spreading, and synapse loss and dysfunction, synergistically accelerating AD progression [40]. Additionally, astrocytes play a crucial role in maintaining the BBB. Excessive activation of astrocytes may affect the BBB permeability, allowing peripheral immune cells and neurotoxic substances to infiltrate the CNS [43], [44]. Recently, studies have found that gut microbiota dysbiosis would interrupt the immune homeostasis and increase toxic gut bacterial metabolites (particularly phenylalanine and isoleucine) in the blood, leading to BBB dysfunction and peripheral immune cell infiltration in the brain, thus triggering neuroinflammation and aggravating neurodegeneration in AD [45].

No therapeutics targeting neuroinflammation have been approved for modification of AD pathogenesis, and even fewer agents are currently in late-stage clinical trials. Non-steroidal anti-inflammatory drugs are the most widely used anti-inflammatory agents in clinical practice and the earliest approaches to AD treatment. However, negative results are found in clinical trials. Notably, cyclooxygenase-2-selective agent rofecoxib significantly increased AD dementia incidence by 46% (P = 0.011) in mild cognitive impairment patients, providing critical evidence that certain anti-inflammatory approaches may exacerbate neurodegeneration and accelerate AD progression [42]. In the early stage of AD, microglia can maintain neuronal function by clearing neurotoxic substances, making modulating microglial activation towards a phagocytic phenotype with anti-inflammatory and neuroprotective effects a promising strategy for AD treatment [40]. AL002, a monoclonal antibody targeting triggering receptor expressed on myeloid cells 2 (TREM2) to promote microglial metabolism and phagocytosis of Aβ, was announced to fail to retard cognitive decline in early-stage AD patients in its phase II trial (NCT04592874), reigniting controversy on the feasibility of microglial targets in AD [46]. Other anti-inflammatory agents under investigation include monoclonal antibodies targeting pro-inflammatory cytokines such as tumor necrosis factor-α, interleukin-1β, and interleukin-6, as well as small molecule inhibitors of multiple cytokine signaling pathways. Sodium oligomannate (GV-971), a mixture of acidic linear oligosaccharides derived from brown algae, was reported to restore the gut microbial profile to normal, reduce brain immune cell infiltration, and mitigate microglia activation-mediated neuroinflammation, thus alleviating cognitive deficits and slowing AD progression. On November 2, 2019, Green Valley announced its conditional marketing approval in China for the treatment of mild to moderate AD patients [5].

Precision delivery for neuroinflammation should primarily focus on disease-associated cell populations and their dynamic activation states. Microglia and astrocytes are critical for maintaining CNS homeostasis; thus, broad exposure to anti-inflammatory agents may compromise their physiological and neuroprotective functions while leading to limited therapeutic efficacy. Precision delivery that preferentially targets over-activated microglia or over-reactive astrocytes could therefore increase drug exposure within disease-relevant inflammatory populations whilst reducing off-target effects on homeostatic glial cells and peripheral immune compartments. Furthermore, the inflammatory microenvironment is characterized by altered levels of ROS, inflammatory mediators, enzymes, and vascular adhesion signals, which could provide additional opportunities for stimulus-triggered accumulation or controlled cargo release.

### 2.4 Oxidative stress hypothesis and targeting therapies

Oxidative stress generally refers to the perturbation of redox homeostasis wherein free radicals, including ROS and reactive nitrogen species (RNS), are overproduced, overwhelming the cellular endogenous antioxidant defenses [47]. Excessive accumulation of free radicals and their derived products attacks lipids, proteins, nucleic acids, and other critical biomolecules, interfering with their physiological structures and/or functions, leading to severe damage to brain cells and tissue. As an organ with high oxygen consumption and unique lipid composition, the brain exhibits heightened vulnerability to free radicals that can easily oxidize polyunsaturated fatty acids, initiating an amplified cascade of redox reactions and eventually leading to neurodegeneration [48]. Perturbation of redox homeostasis is considered a bridge that connects different AD pathogenic mechanisms as well as an intrinsic molecular aspect of these pathogeneses, including Aβ deposition, tau hyperphosphorylation, mitochondrial dysfunction, calcium imbalance, and neuroinflammation [48]. Together with the above AD pathogeneses, oxidative stress acts synergistically to cause synaptic impairment and neuronal dysfunction, thus accelerating CNS neurodegeneration and AD progression [49].

Antioxidant-based therapies, including natural or synthetic compounds, inorganic molecules, and vitamins, can exert beneficial effects in the brain by inhibiting chain reactions involving the production of ROS/RNS, improving endogenous antioxidant defenses, and restoring or removing the impaired cell membranes and other biomolecules [50]. Natural compounds, mostly polyphenols and flavonoids extracted from plants or microorganisms, have gained increasing interest for their remarkable capacity to combat oxidative stress and mitigate oxidative damage. Among these molecules, resveratrol, ferulic acid, curcumin, quercetin, and epigallocatechin-3-gallate have been widely studied in current preclinical studies and clinical trials for AD [51]. Nanozymes are recently developed nanomaterials that mimic various natural enzyme activities, such as superoxide dismutase, catalase, and peroxidase, thereby scavenging multiple free radicals [52]. Compared to natural enzymes, nanozymes have enhanced structural stability, lower cost, higher functional versatility, and better catalytic efficiency, making them a research hotspot for AD treatment [53]. Promising results have been observed in many preclinical studies of antioxidant-based therapies on AD animal models, which, however, are contradictory to the results in human studies. The therapeutic outcomes of these antioxidants in AD patients are disappointing, failing to alleviate cognitive impairment and retard AD progression [48]. This dilemma may result from the insufficient pharmacological concentrations in AD lesions due to rapid metabolism and clearance, poor BBB permeability, and low bioavailability of these agents, posing a great challenge to translate them from bench to bedside for AD antioxidant-based treatment.

Oxidative stress offers a distinct opportunity for precision delivery, as elevated ROS can serve both as a therapeutic target and as an endogenous trigger for site-selective drug release. ROS-responsive nanocarriers can specifically release their payloads in oxidative pathological microenvironments, thereby increasing local pharmacological activity while limiting unnecessary exposure in unaffected brain regions. This property may be particularly beneficial for antioxidants with rapid clearance, poor BBB penetration, or limited systemic bioavailability. More importantly, given the extensive crosstalk between oxidative stress and other AD pathological processes, redox-responsive nanocarriers also provide a promising platform for co-delivering antioxidants with therapeutics targeting interconnected pathological pathways.

### 2.5 Neurovascular unit (NVU) dysfunction and targeting therapies

Neurovascular unit dysfunction has become recognized as an important contributor to AD progression. The NVU, which comprises BMECs, pericytes, astrocytic end-feet, neurons, and microglia, plays a critical role in maintaining BBB integrity, regulating cerebral blood flow and neurovascular coupling, and supporting nutrient exchange and the clearance of neurotoxic metabolites. Growing evidence indicates that neurovascular abnormalities can arise early in the AD continuum, with capillary damage and BBB breakdown observed in individuals with early cognitive decline [54]. NVU dysfunction is characterized by increased vascular permeability and dysregulated BBB influx/efflux systems, leading to impaired oxygen and nutrient supply, reduced vascular Aβ clearance, and increased entry of exogenous toxic substances, all of which collectively drive AD progression. Moreover, previous research has reported that the receptor for advanced glycation end products (RAGE) is upregulated at the BBB, where it binds to circulating Aβ and promotes its influx into the brain, thereby exacerbating Aβ burden [55]. Furthermore, Aβ pathology can in turn exacerbate NVU dysfunction, with evidence showing that Aβ promotes pro-inflammatory signaling in pericytes, subsequently inducing tight-junction deficits and impairing adjacent endothelial cells, thereby establishing a vicious cycle that accelerates disease progression [56].

NVU modulation has been proposed as a potential disease-modifying strategy for AD, involving restoring BBB integrity and physiological Aβ transport, preserving pericyte function and cerebral microcirculation, and suppressing vascular inflammation and oxidative stress. Experimental evidence indicates that normalizing NVU function may concurrently improve cerebrovascular homeostasis and interrupt multiple downstream AD pathological cascades [57]. Several NVU-related interventions have entered clinical evaluation. Fasudil, a Rho kinase inhibitor with vasodilatory and neuroprotective activities, is currently being evaluated in a phase 2 trial in patients with early AD [58]. Another ongoing trial is examining whether intensive systolic blood pressure control, mainly using losartan and amlodipine, can alter cerebral perfusion as well as brain Aβ and tau deposition in cognitively normal older adults at increased risk of AD. In parallel, the mTOR inhibitor sirolimus (rapamycin) is being evaluated in a phase 2 trial in patients with amnestic mild cognitive impairment or early AD, supported by preclinical evidence showing that rapamycin restores cerebral blood flow and vascular integrity through activation of endothelial nitric oxide synthase, thereby reducing Aβ burden and enhancing cognitive function in AD mice. Nevertheless, definitive clinical evidence that NVU modulation can attenuate AD progression remains lacking.

In AD, the NVU and BBB serve not only as barriers to drug delivery but also as therapeutic targets. Therefore, for precision delivery of NVU-directed therapies, it may be preferable to retain drugs within the pathological NVU rather than to promote their transcytosis into the brain parenchyma. Moreover, disease-associated vascular alterations, such as upregulated endothelial RAGE expression, inflammatory activation, oxidative stress, or other pathological changes of damaged vessels, can be exploited for targeted delivery to the dysfunctional NVU while reducing unintended modulation of healthy vasculature [59].

### 2.6 Other pathogenesis and targeting therapies

Mitochondrial dysfunction is also an important contributor to AD onset and progression. As the central organelle of cellular energy metabolism, mitochondria generate adenosine triphosphate (ATP) through the electron transport chain while also producing substantial amounts of ROS. Additionally, mitochondria play a crucial role in the maintenance of intracellular and extracellular calcium homeostasis [60]. Under AD pathology, the impaired mitochondria in neurons have a limited ability to regulate calcium equilibrium, resulting in intracellular calcium overload, which can exacerbate oxidative stress and compromise ATP production, and finally lead to energy depletion and neuronal death [61]. Concurrently, excessive ROS further attacks mitochondria and aggravates mitochondrial dysfunction, creating a self-perpetuating vicious cycle of oxidative stress and mitochondrial damage [49]. Antioxidants can mitigate mitochondrial damage by scavenging excessive ROS, thereby restoring mitochondrial function. Dimebon (Latrepirdine), an antihistamine proposed to be neurogenic and to exert protective effects on neuronal mitochondria, has been tested in mild to moderate AD patients [62]. Moreover, emerging evidence suggests that mitophagy function, the selective autophagic removal of damaged or dysfunctional mitochondria, is impaired in AD, leading to the excessive or aberrant removal of healthy mitochondria in neurons and further aggravating ATP depletion. Mitophagy impairment can also lead to a cascade of pathological consequences, including misfolded Aβ and tau accumulation, microglial activation, synaptic function and plasticity damage, synergistically contributing to neurodegeneration and AD progression [63]. Thus, mitophagy-enhancing agents, such as nicotinamide riboside and USP30 inhibitors, represent a compelling strategy for addressing the mitochondrial pathophysiology in AD [64]. For precision delivery of mitochondria-targeting agents, successful BBB penetration and brain accumulation are merely the initial steps. The therapeutic cargo must subsequently reach vulnerable neuronal populations, undergo efficient cellular internalization, overcome intracellular trafficking barriers, and ultimately access dysfunctional mitochondria. Accordingly, hierarchical delivery systems that integrate brain entry, neuron-selective targeting, and mitochondrial localization could substantially increase the therapeutics that reach their actual site of action.

The cholinergic hypothesis is one of the earliest theories for the pathogenesis of AD. Cholinergic neurons are widely distributed in the CNS and provide extensive cholinergic projections to the cerebral cortex and hippocampus. Acetylcholine (ACh) serves as a key excitatory neurotransmitter critically involved in advanced cognitive processes such as learning, memory, and attention. Previous studies have demonstrated severe degeneration and atrophy of the basal forebrain, particularly the nucleus basalis of Meynert, in AD patients, accompanied by a significant loss of cholinergic neurons, which collectively leads to impaired synthesis, storage, and release of ACh [65], [66]. Evidence has shown that even in surviving cholinergic neurons, there is a marked reduction in choline acetyltransferase (ChAT) activity, loss of acetylcholine receptors (AChRs), and depletion of presynaptic ACh. Consequently, the disruption of central cholinergic signaling and decreased ACh levels contribute directly to cognitive decline in AD patients, with the severity of impairment correlating closely with the extent of cholinergic damage [67]. The cholinergic hypothesis has served as a foundational framework that advances early-stage research and therapeutic development for AD. AChEIs, including donepezil, galantamine and rivastigmine, have been approved by the FDA to increase synaptic ACh levels in AD patients, representing the mainstay of AD drugs in clinical treatment [68]. However, these agents fail to halt disease progression and offer only symptomatic relief, exhibiting limited efficacy and adverse effects in severe AD, highlighting the need for more potent AChEIs or combining with other DMT drugs to improve therapeutic effects and quality of life for AD patients [9]. Precision delivery of cholinergic-targeting therapies requires nanocarriers that can preferentially accumulate in vulnerable cholinergic regions or interact with specific cholinergic neurons. For conventional AChEIs that primarily enhance synaptic ACh levels, regional enrichment may improve therapeutic efficacy without necessitating extensive intracellular delivery, whereas potential regenerative or gene-regulatory agents may additionally require neuronal internalization to exert their effects. Importantly, given that cholinergic therapies are predominantly symptomatic, nanoparticle-mediated co-delivery of cholinergic modulators with DMTs may offer a promising strategy to combine symptomatic improvement with modification of the underlying AD process.

Metal ion dyshomeostasis is probably not the primary cause of AD, but might be closely associated with AD development and progression. Metal ions, including Fe^2+^, Cu^2+^, and Zn^2+^, are involved in maintaining cellular structure, regulating enzyme activity, and modulating redox balance, synaptic plasticity, and signaling pathways related to learning and memory, playing a vital role in the maintenance of normal neurophysiological function [69]. Substantial evidence has demonstrated the disrupted metal ion equilibrium in the brains of AD patients, which can promote Aβ aggregation, oxidative stress, synaptic damage, and neuronal death, thereby collectively accelerating AD progression [70], [71]. For example, aberrant accumulation of metal ions potentially impairs the activity of critical enzymes, altering the conformation of proteins, and interfering with their clearing pathways, thus ultimately leading to inappropriate deposition of misfolded proteins such as Aβ and hyperphosphorylated tau [72]. In addition, excessive accumulation of metal ions within protein aggregates can trigger a cascade of ROS generation, contributing to metal-induced proteotoxicity and oxidative stress, and eventually exacerbating synaptic dysfunction and neuronal loss [70]. Further research is essential to clarify the mechanistic role of metals in AD. Metal ion chelators, such as the Fe^3^^+^ chelator deferoxamine and the Cu^2+^/Zn^2+^ chelator clioquinol, have garnered extensive interest for remodeling metal homeostasis, and related preliminary clinical trials are currently underway [69]. Since aberrant metal accumulation simultaneously promotes Aβ aggregation and ROS generation, nanocarriers capable of recognizing Aβ or responding to redox microenvironments are promising for increasing local chelating activity while minimizing disruption of physiological metal homeostasis. Moreover, metal chelation is particularly suitable for combination delivery with anti-Aβ or antioxidant agents, allowing a single nanoplatform to concurrently modulate multiple interconnected pathogenic processes.

## 3 Barriers and administration routes for precision AD drug delivery

Effective precision drug delivery in AD requires therapeutics to overcome sequential barriers from systemic circulation to their final site of action within the brain [73]. After systemic administration, drugs and delivery systems are subject to renal and hepatic clearance, uptake by the mononuclear phagocyte system (MPS), enzymatic degradation, and nonspecific interactions with circulating proteins, all of which reduce the proportion that ultimately reaches the cerebral vasculature [74]. In addition, protein adsorption can further alter the biological identity and targeting properties of delivery systems [75]. Moreover, the BBB constitutes the primary anatomical barrier to brain entry. Tight junctions between BMECs, low basal vesicular transport, and active efflux systems collectively restrict paracellular and transcellular transport. Importantly, BBB dysfunction in AD is heterogeneous, characterized by varying degrees of endothelial injury, increased permeability, and altered transporter and receptor expression, such as glucose transporter 1 (GLUT1) and low-density lipoprotein receptor-related protein-1 (LRP-1), making brain entry variable across disease stages and individuals [76]. After crossing the BBB, therapeutics must diffuse through the highly tortuous and charged brain extracellular space, where extracellular matrix components, interstitial fluid and cerebrospinal fluid (CSF) flow, perivascular deposits, and disease-associated deposits can limit tissue penetration and residence time [77]. Finally, therapeutics directed toward intracellular targets must be taken up by disease-relevant cell populations and escape endosomal-lysosomal sequestration to reach the cytosol or specific organelles [78]. Cell-type heterogeneity and disease-dependent receptor expression further complicate selective delivery to neurons, microglia, and astrocytes [75]. Therefore, successful precision delivery requires coordinated control of systemic exposure, BBB crossing, lesion-specific distribution, cell-selective targeting, and intracellular bioavailability.

Administration route also critically influences brain exposure, targeting precision, degree of invasiveness, and clinical feasibility in AD therapy. Systemic administration, including oral and intravenous delivery, remains the most clinically practical approach for chronic treatment. Oral dosing is convenient and suitable for repeated administration but is limited by gastrointestinal degradation, first-pass metabolism, variable bioavailability, and the subsequent requirement for therapeutics to cross the BBB. Intravenous administration provides rapid and complete systemic exposure, particularly compatible with biologics and engineered delivery systems. However, peripheral clearance, off-target organ accumulation, and restricted BBB penetration remain major limitations [79]. Intranasal administration has attracted increasing interest as a non-invasive alternative that can partially bypass the BBB through olfactory and trigeminal pathways, potentially reducing systemic exposure and enabling rapid brain access. Nonetheless, its efficiency is constrained by limited dosing volume, mucociliary clearance, enzymatic degradation, and poor mucosal permeability [80]. Direct delivery into the CSF through intrathecal or intracerebroventricular administration can bypass the BBB, particularly attractive for macromolecules and nucleic-acid therapeutics. However, rapid CSF turnover, limited penetration from CSF into the deep brain parenchyma, and the risks and burden associated with repeated invasive administration restrict their clinical application [81]. Intraparenchymal delivery can achieve high local drug concentrations and minimize peripheral exposure, but is highly invasive and provides limited coverage of the diffusely affected AD brain [79]. Therefore, the administration route should be determined by cargo properties, anatomical target, required spatial coverage, dosing frequency, and disease stage.

## 4 Nanoparticle-based delivery systems for AD drug delivery

Nanoparticles are nanoscale materials with dimensions typically ranging from 1 to 1000 nm, exhibiting unique physicochemical properties due to their high surface area and small size. Based on their composition and properties, they can be generally classified into polymeric, lipid-based, inorganic, and biomimetic nanoparticles, which provide versatile platforms for therapeutic loading and precision delivery in AD (Fig. 2). Their diverse architectures and high surface-area-to-volume ratios enable the loading, encapsulation, or conjugation of various therapeutic cargos and facilitate the co-delivery of multiple agents, providing opportunities for multi-target combination therapy. In addition, surface modification and functionalization can enhance BBB transport and facilitate preferential targeting of AD-associated pathological regions and diseased cell populations, thereby enabling more precise drug delivery and controlled release at disease sites.

**Fig. 2:**
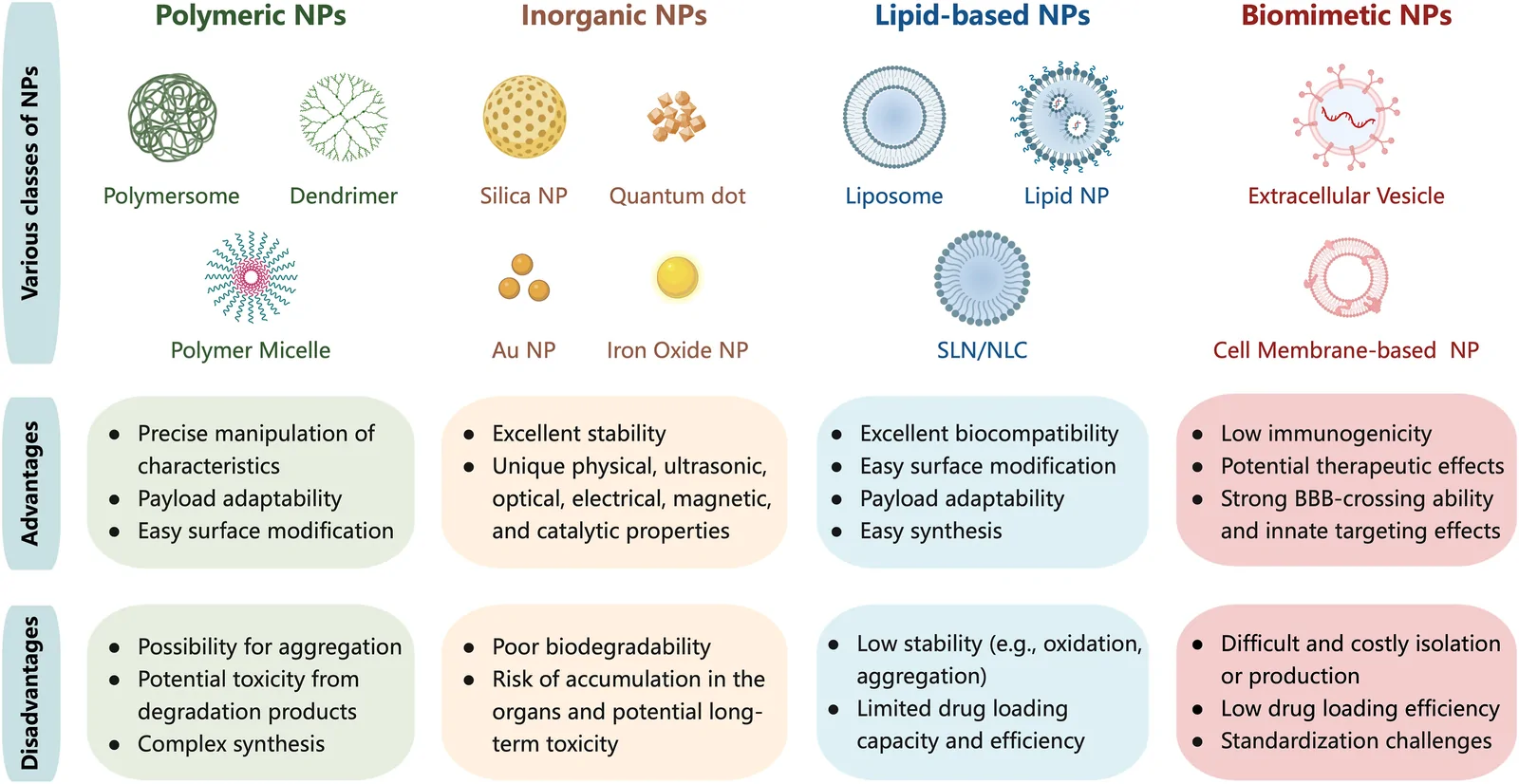
**Various classes ofnanoparticlesas well as their advantages and disadvantages for AD drug delivery.** Created in BioRender. Shan, J. (2026) https://BioRender.com/wcjhxwf.

Notably, in addition to these carrier-based categories, carrier-free nanodrugs have emerged as an alternative formulation strategy in which therapeutic molecules themselves constitute the major components of the nanostructure through self-assembly or drug-drug conjugation. These systems exhibit high drug loading capacity, reduced carrier-associated toxicity, and relatively simple synthetic procedures [82]. Recent studies have demonstrated their potential for AD therapy. For example, Yang et al. developed an intranasal nanomodulator self-assembled by donepezil and simvastatin through noncovalent interactions, which collectively attenuated neuroinflammation and oxidative stress, improved cognitive performance, and restored brain microenvironmental homeostasis in FAD^4T^ transgenic mice [83]. More recently, a ROS-responsive carrier-free nanosystem integrating fingolimod with siBACE1 was developed to repolarize microglia and decrease Aβ production, highlighting the potential of this simple formulation for AD combination therapy [84]. However, the clinical translation of carrier-free nanoparticles remains challenging, impeded by unpredictable self-assembly, insufficient stability and delivery efficiency, limited control over pharmacokinetics, and an incompletely understood *in vivo* fate, which requires further systematic investigation [82].

### 4.1 Polymeric nanoparticles

Polymeric nanoparticles are synthesized from natural or synthetic homopolymers or copolymers, comprising polymersomes, dendrimers, and polymer micelles [73]. As a delivery system, these nanoparticles are promising candidates due to their desirable tunability, payload adaptability, biodegradability, long circulation, and controllable drug release [85]. In addition, through rational formulation design and surface functionalization, their targeting precision and controllability can be significantly enhanced, making them effective vehicles for brain-targeted drug delivery in AD [86].

Polymersomes are artificial vesicles that can be easily synthesized from different polymers, including natural materials, such as alginate, chitosan, collagen, and albumin, as well as synthetic alternatives, such as poly(lactide-co-glycolide) acid (PLGA), poly(ethylene glycol) (PEG), polycaprolactone (PCL), and poly(dimethylsiloxane) [87]. PLGA, a biodegradable copolymer of lactic acid and glycolic acid, is one of the most widely investigated biomedical material for AD therapy. Recently, Lei, T. et al. used α-mannopyranoside-modified PLGA-PEG as the structural skeleton, of which the hydrophilic and electronegative properties contributed to the effective penetration through the mucus barrier after oral administration (Fig. 3A) [88]. The grafted α-mannopyranoside facilitated the co-targeting and permeation of the intestinal epithelial barrier and BBB via a glycemic control strategy. Then, the encapsulated fingolimod accumulated effectively in the brain, where it modulated aberrant hyperactivation of microglia and astrocytes and facilitated the remodeling of the AD lesion microenvironment. This intelligent brain-targeting polymeric nanocarrier represents a promising strategy for effective orally administered treatment in AD.

**Fig. 3:**
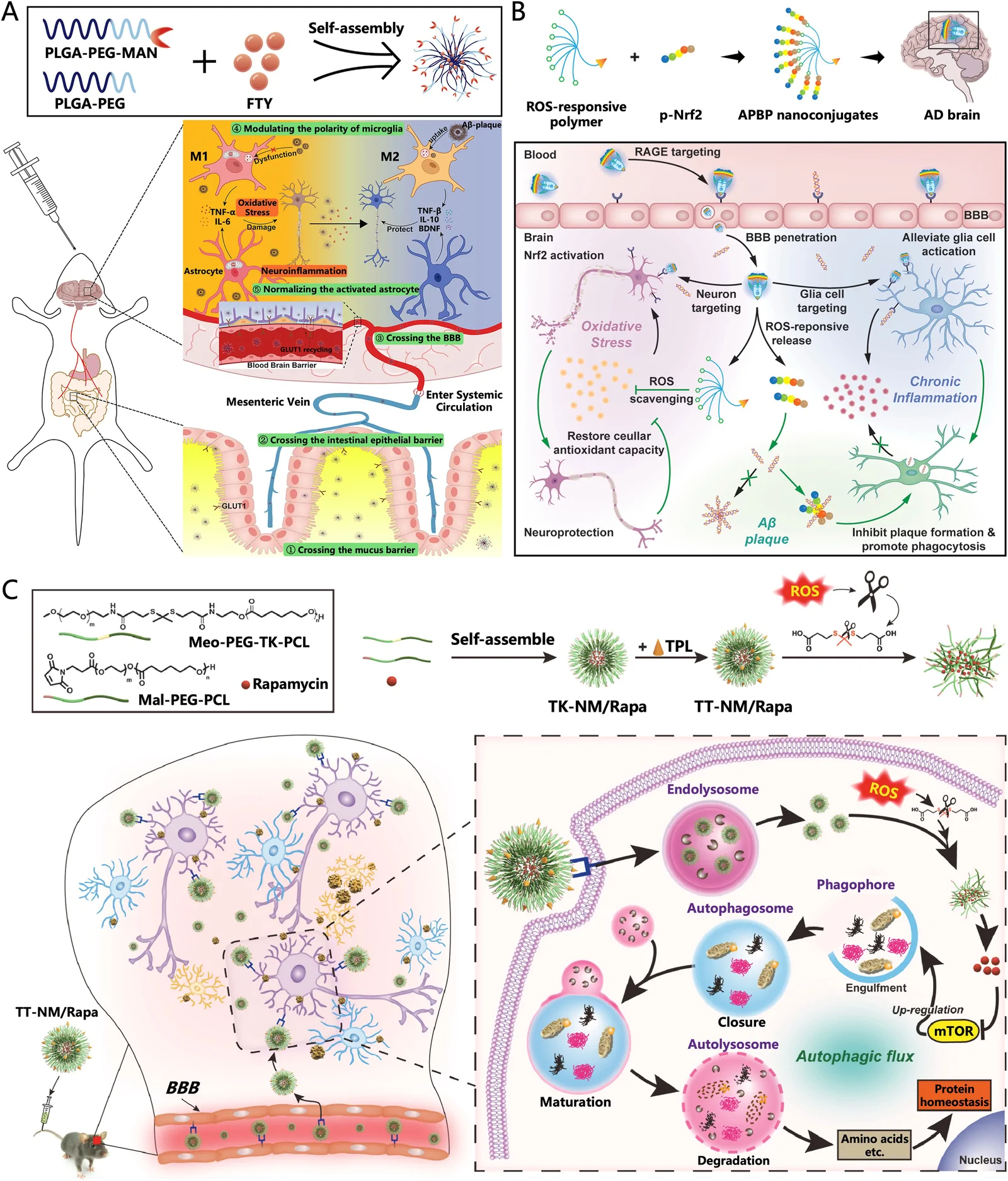
**Polymeric nanoparticles for AD treatment.** (A) Schematic illustration of the intelligent oral brain-targeting nanoparticle based on a PLGA-PEG framework, encapsulating fingolimod and surface-functionalized with mannose for multi-target AD therapy. Reproduced with permission [88]. Copyright 2024, American Chemical Society. (B) Schematic illustration of the biomimetic dendrimer-peptide nanoconjugate for modulating AD microenvironment by restoring cellular antioxidant ability, alleviating glial cell activation, and promoting Aβ phagocytosis. Reproduced with permission [89]. Copyright 2021, John Wiley and Sons. (C) Schematic diagram of the ROS-responsive and neuron-targeted micellar system for improving neuronal proteostasis by upregulation of autophagic flux. Reproduced with permission [90]. Copyright 2022, Xu et al.

Dendrimers are three-dimensional polymeric materials with tree-like architectures, consisting of hydrophobic cores with a high degree of repetitive branches that expand outward. The unique hierarchical structures of dendrimers enable the stepwise addition of branching units via iterative synthesis methods, such as divergent or convergent growth, providing abundant active surface sites at the exterior [73]. Hence, therapeutic agents can be loaded at the core of the dendrimers, whilst multiple functional biomolecules can be conjugated to the reactive groups on the surface for more effective and precise drug delivery. Liu et al. designed a biomimetic dendrimer-peptide nanoconjugate (APBP) composed of Ab peptides (KLVFFAED), PEG-based phenylboronic dendrimer, and nuclear factor (erythroid-derived 2)-like 2 (Nrf2) peptides (Fig. 3B). After intravenous administration, APBP effectively penetrated the BBB and bound to the overexpressed RAGE in AD lesions via Ab peptide, scavenging ROS and releasing Nrf2 peptides, synergistically contributing to the mitigation of inflammatory responses and neuroprotective effects in APPswe/PSEN1dE9 (APP/PS1) AD mice [89]. Notably, studies have shown that dendrimers can easily traverse the slightly impaired BBB and selectively target the hyperactive plaque-associated microglia (PAM) in AD pathology [[91], [92]]. Henningfield et al. used hydroxyl dendrimers to deliver a colony-stimulating factor-1 receptor inhibitor for the therapy of transgenic mice with five familial AD (5×FAD) mice [91]. Results illustrated that this systemically injected nanomedicine could cross the BBB and was preferentially internalized by PAMs in the brain while leaving other cell types unaffected, leading to a significant decrease in Aβ deposition, active PAM, and pro-inflammatory gene transcription in 5×FAD mice.

Micelles comprise amphiphilic block copolymers that form core-shell nanospheres through self-assembly in aqueous solution, featuring hydrophobic cores inward and hydrophilic terminals outward [73]. This design serves to encapsulate hydrophobic therapeutics within the inner cores, with hydrophilic coronas stabilizing the micelles and mitigating opsonization in physiological environments, thereby protecting them from the reticuloendothelial system and prolonging their systemic circulation [87]. Furthermore, micelles can be easily functionalized with a variety of targeting ligands as well as stimuli-responsive motifs, allowing for site-specific drug delivery and spatiotemporal control over payload release. Considering the oxidative environment in AD neurons, Xu et al. reported a novel micelle system exhibiting high brain-neuron targetability and desirable ROS-responsive drug release. The system was constructed using a methoxy-PEG-PCL copolymer incorporating a ROS-cleavable thioketal moiety as the polymeric backbone, with further surface modification by the fusion peptide TPL (Fig. 3C) [90]. TPL, comprised of BBB-targeting peptide TGN and GT1b ganglioside receptor-binding peptide Tet1, significantly enhanced drug delivery efficiency to AD-affected neurons. After the uptake by neurons with high levels of ROS, the system underwent rapid ROS-triggered disassembly, accelerating rapamycin release and enhancing its interaction with related proteins in the cytoplasm. Consequently, this micelle system greatly restored autophagic flux and recovered neuronal proteostasis, contributing to the amelioration of memory defects and delay of disease progression of triple transgenic (3×Tg) AD mice.

### 4.2 Lipid-based nanoparticles

Lipid-based nanoparticles are artificial nanocarriers with phospholipid monolayers, bilayers or multiple bilayers consisting of liposomes, lipid nanoparticles (LNPs), solid lipid nanoparticles (SLNs), nanostructured lipid carriers (NLCs), and hybrid lipid nanoparticles. These nanoparticles possess highly diversified drug delivery capacities, as lipophilic drugs can be loaded in the lipid layer, while hydrophilic drugs can be loaded in the aqueous interior core. Meanwhile, different agents can also be linked to the surface of lipid-based nanoparticles through physical adsorption or covalent conjugation, providing a large variety of methods for drug loading [93]. In addition, they exhibit many other advantages, including high biocompatibility and biodegradability, low immunogenicity, and simplicity in synthesis and surface modification that facilitates targeted delivery and controlled release in therapeutic applications [94].

Liposomes are among the most extensively investigated nanomaterials for drug delivery, with well-established biocompatibility and substantial clinical experience across several approved formulations [95]. Structurally, they are hollow spherical nanovesicles with bilayers of amphiphilic phospholipid molecules surrounding the aqueous core. Due to their bilayer structures, hydrophobic and hydrophilic drugs can be respectively carried in the phospholipid bilayers and the hydrophilic core. Different lipid components involved in the formulation can lead to liposomes with different electric charges, including anionic, cationic, and neutral liposomes [96]. Notably, positively charged liposomes exhibit enhanced binding affinity to the negatively charged BMECs of the BBB, thus promoting more efficient drug transportation to the brain. Moreover, the biomimetic structure of phospholipid bilayers enables easy modifications of multiple functional ligands to liposomes, facilitating more precise and efficient drug delivery to AD lesions. Recently, Su et al. designed a liposomal nanodrug (DPMT@PEI/miR-195) for the brain-targeted delivery of a polyethyleneimine (PEI)/miR-195 complex (Fig. 4A) [97]. The nanodrug was modified with P-aminophenyl-α-d-mannopyranoside and the cell-penetrating peptide to enhance BBB transport through receptor-mediated and adhesion-mediated uptake, respectively. In APP/PS1 models, DPMT@PEI/miR-195 reduced Aβ production, tau phosphorylation, and M1-like microglial polarization. Under the experimental conditions used, DPMT@PEI/miR-195 exhibited a similar degree of cognitive improvement to that observed in donepezil- and aducanumab-treated groups, although differences in dosing regimens and administration routes preclude direct conclusions about therapeutic superiority or equivalence. These findings support the preclinical potential of this miR-195 liposomal formulation for targeting multiple AD-related pathological processes.

**Fig. 4:**
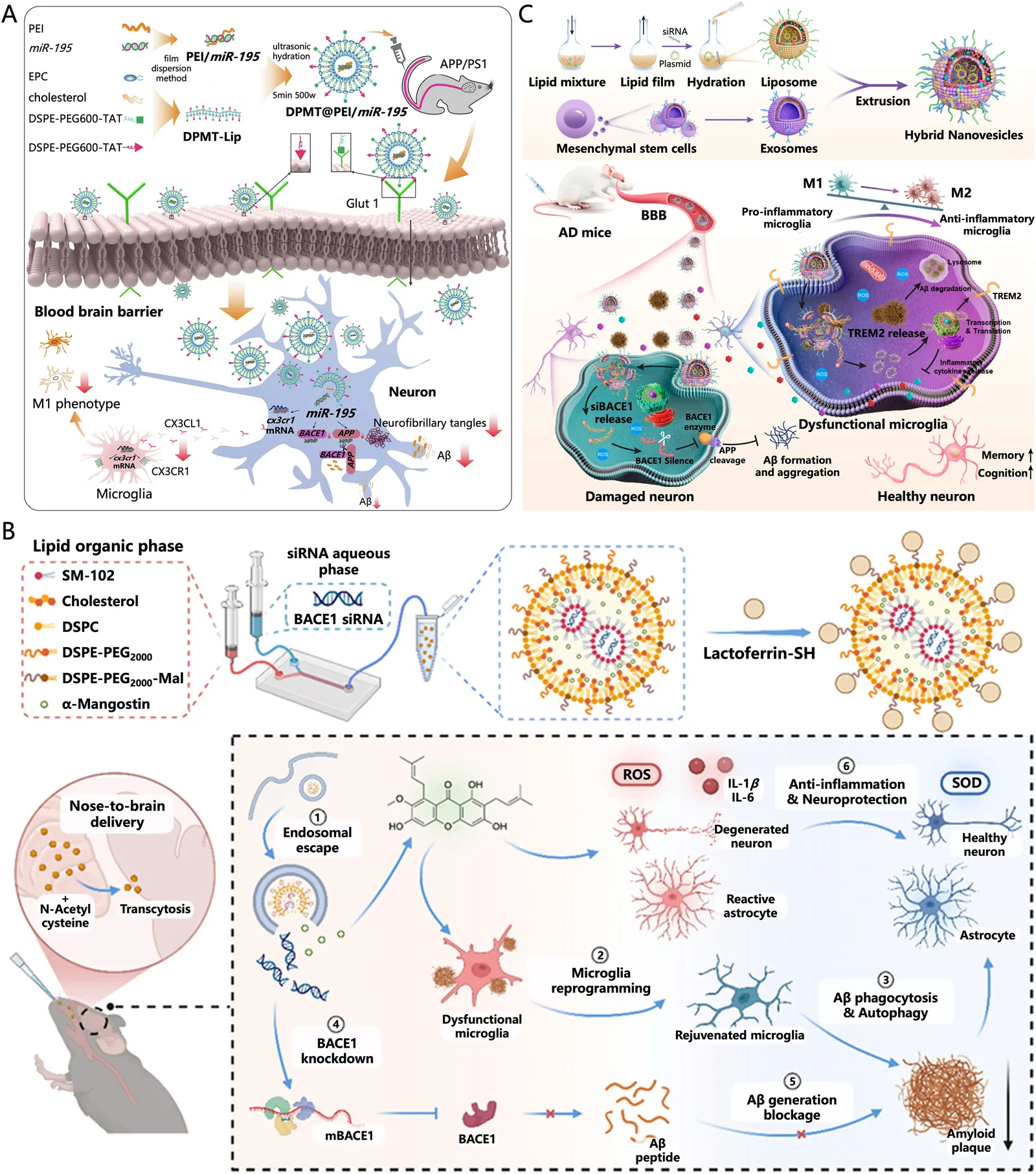
**Lipid-based nanoparticles for AD treatment.** (A) Schematic illustration of brain-targeted liposomal nanodrug (DPMT@PEI/miR-195) to inhibit Aβ generation, tau phosphorylation, and M1 microglial polarization in AD mice. Reproduced with permission [97]. Copyright 2024, Elsevier. (B) Schematic illustration of the lactoferrin-functionalized LNPs for the co-delivery of α-mangostin and BACE1 siRNA to collectively restore the abnormal microenvironment in AD brains. Reproduced with permission [98]. Copyright 2025, Elsevier. (C) Schematic illustration of exosome-liposome hybrid nanovesicle using membrane fusion technology for the co-delivery of BACE1 siRNA and a TREM2 plasmid to ameliorate the cognitive deficit in APP/PS1 mice. Reproduced with permission [99]. Copyright 2024, American Chemical Society.

LNPs are liposome-like lipid-based nanoparticles that are commonly exploited for nucleic acid delivery, including plasmid DNA, microRNA, messenger RNA, small interfering RNA (siRNA), and ASOs, providing a promising platform for gene-based therapies of AD [[100], [101]]. Typical LNPs comprise four major components: ionizable lipids, cholesterol, phospholipids, and PEGylated lipids. Ionizable lipids exhibit positive charges at acidic pH and encapsulate negatively charged nucleic acids through electrostatic interactions, thus protecting payloads from enzymatic degradation, promoting endosomal escape, and facilitating cellular uptake [[102], [103]]. Meanwhile, they remain electrically near-neutral in physiological environments, which significantly enhances their biocompatibility and reduces their toxicity. Xu et al. developed an innovative lactoferrin-functionalized LNP via a microfluidic approach for the co-delivery of α-mangostin and BACE1 siRNA, combining the neuroprotection and dysfunctional microglia reprogramming abilities of α-mangostin with the transcriptional silencing of the key enzyme in Aβ generation by BACE1 siRNA (Fig. 4B) [98]. *In vivo* investigations illustrated that this intricate LNP system remarkably remodeled the aberrant lesion microenvironment and restored cognitive impairment through distinct yet complementary mechanisms, providing a promising strategy for multi-pathway treatment of AD. Badr, A. et al. formulated a mannose-coated LNP loading microRNA-17 antagomir to treat 5×FAD mice [104]. Results showed that after intra-cisterna magna injection, microRNA-17 antagomir LNPs significantly downregulated microRNA-17 expression in microglia, decreased inflammation and Aβ deposition, improved autophagy, and alleviated spatial memory deficits and anxiety-like behavior in AD mice.

SLNs are colloidal systems featuring solid lipid cores stabilized by peripheral amphiphilic surfactants [105]. Various drugs, including Huperzine A, galantamine, quercetin, piperine, curcumin, and other anti-AD agents, have been encapsulated in SLNs for AD treatment, exhibiting better bioavailability and therapeutic efficacy than the free drugs [[106], [107], [108], [109]]. Compared with SLNs, NLCs, considered as the next generation of SLNs, are lipid-based nanoparticles fabricated from a fusion of solid and liquid lipids, leading to structural rearrangements and a less ordered crystalline configuration. This unique structure with reduced crystallinity increases the drug loading capacities and prevents payload leakage from the matrix, and thus exhibits higher storage stability than SLNs [87]. Khonsaria et al. developed transferrin-decorated NLCs (Tf-NLCs) loading with rapamycin for the treatment of AD rats [110]. Compared to the rapamycin solution-treated group, Tf-NLCs not only protected the rapamycin from chemical and enzymatic degradation but also abolished the immunosuppressive effects and delayed wound healing adverse reactions of rapamycin, facilitating the efficient drug BBB crossing and accumulation in the brain of AD rats.

Hybrid lipid nanoparticles have emerged as novel nanocarriers by integrating lipid-based nanoparticles with other materials, such as exosomes and cell membranes, to overcome the drawbacks of single-component nanoparticles [94]. Jiang et al. designed an exosome-liposome hybrid nanocarrier based on membrane fusion technology, achieving BACE1 siRNA and TREM2 plasmid co-delivery for AD therapy (Fig. 4C) [99]. Owing to the BBB-targeting ability of angiopep-2 (Ang-2) peptide conjugated to liposomes and the homing ability of exosomes, the biomimetic nanovesicles could efficiently penetrate the BBB and actively migrate to AD lesion sites. Then, BACE1 siRNA downregulated the BACE1 expression and reduced the production of Aβ, while TREM2 plasmid upregulated the TREM2 expression and reprogrammed the activated microglia phenotype, thus collectively ameliorating the cognitive deficit in APP/PS1 mice. Overall, this hybrid lipid nanoparticle achieved combined gene therapy and offered more options for synergistic AD therapy.

### 4.3 Inorganic nanoparticles

Inorganic nanoparticles, including gold nanoparticles (AuNPs), quantum dots (QD), iron oxide nanoparticles, and silica, have demonstrated significant potential in both diagnostic and therapeutic applications for AD. Despite being slightly inferior in terms of biocompatibility and safety compared to organic nanoparticles, they exhibit superior stability, greater flexibility in manipulating size and morphology, and higher drug delivery efficiency. More importantly, inorganic nanoparticles are characterized by their unique physical, ultrasonic, optical, electrical, magnetic, and catalytic activities, enabling versatile functions in disease diagnosis, controlled drug delivery, and nanoparticle-based therapy for AD [111], [112]. For example, inorganic nanoparticles have been widely employed in biomedical imaging, magnetic or ultrasonic-mediated delivery, photothermal or photodynamic therapy, as well as nanozyme-based catalytic therapy [113]. AuNPs, known for their exceptional photothermal conversion efficiency and remarkable photostability, have been extensively used in photothermal therapy for photothermal ablation of Aβ aggregates in AD lesions [19]. Moreover, the local thermal effects of AuNPs can transiently enhance the BBB permeability, thereby promoting passive accumulation of therapeutics in the brain. QD are nano-sized semiconducting materials with specific optoelectrical properties that enable their widespread applications in high-quality bio-imaging and targeted therapeutic agent delivery [114]. As illustrated in Fig. 5A and B, Huang et al. encapsulated AgAuSe QD in PLGA nanoparticles coated with rabies virus glycoprotein (RVG)-overexpressed neural stem cell (NSC) membrane, achieving real-time in situ visualization of the brain-targeted delivery process *in vivo* from systemic circulation and BBB penetration down to the single-cell scale via QD-based near-infrared-II imaging [115]. Iron oxide nanoparticles, including magnetite (Fe_3_O_4_) or maghemite (Fe_2_O_3_), have been widely applied as magnetic resonance imaging (MRI) contrast agents, drug delivery vehicles, or thermal-based therapeutics. Importantly, due to their unique magnetic properties, these nanoparticles can be precisely guided to target regions using external magnetic fields, thus enabling spatiotemporally controlled drug delivery. Mesoporous silica nanoparticles (MSNs) feature a honeycomb-like porous structure, providing high specific surface area and substantial pore volume that enable extremely high loading efficiency of different types of AD drugs [116]. In addition, MSNs can also serve as a substrate nanocarrier to load other nanoparticles, forming advanced inorganic nanocomposites with different properties for multiple biomedical applications. Chen et al. designed a multifunctional integrated system based on MSN, surface-modified with ultrasmall ceria and iron oxide nanoparticles (Fig. 5C and D) [117]. In this system, ceria exerted its enzyme-mimetic and ROS scavenging effects, while iron oxide acted as the high-resolution MRI media that enabled bimodal imaging to trace the nanocomposite *in vivo* (Fig. 5E). Methylene blue, which was encapsulated within the mesopores of the MSN, further inhibited tau aggregation and neuronal apoptosis, collectively leading to the alleviation of memory impairment in AD rats. Overall, inorganic nanocomposites can integrate the diverse properties of multiple nanoparticles to meet different requirements in application and provide a promising candidate for developing synergistic therapies against AD.

**Fig. 5:**
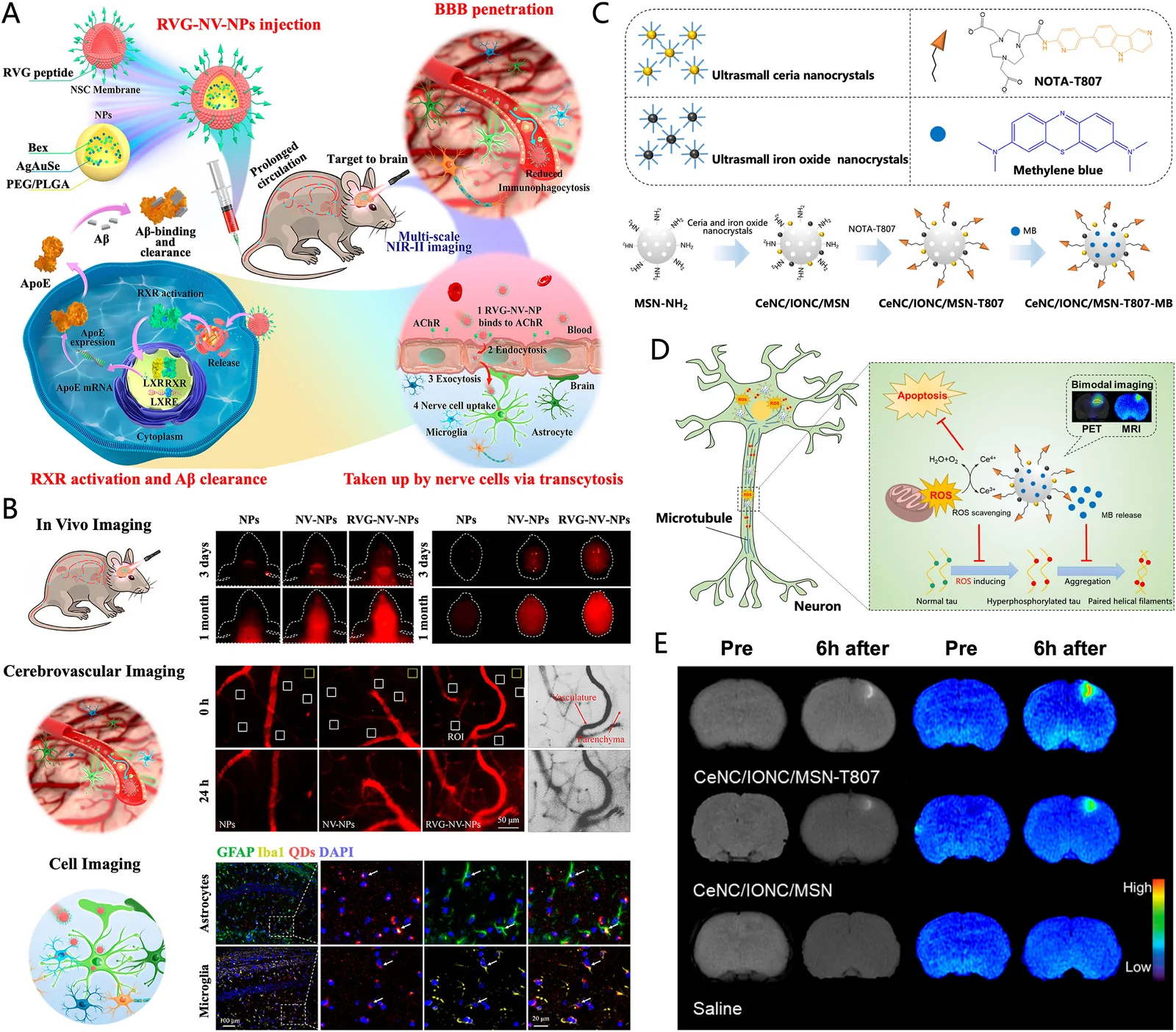
**Inorganic nanoparticles for AD treatment.** (A) Schematic illustration of RVG-overexpressed NSC membrane-coated nanoparticles (RVG-NV-NPs) for brain-targeted drug delivery in APP/PS1 mice. (B) Schematic illustration of *in vivo* RVG-NV-NPs tracking in APP/PS1 mice by multiscale AgAuSe QD-based near-infrared-II imaging, as well as representative fluorescence images of whole-brain distribution, extravasation from cerebral vasculature into brain parenchyma, and colocalization of QDs with microglia or astrocytes in hippocampal sections. Reproduced with permission [115]. Copyright 2023, American Chemical Society. (C-D) Schematic illustration of the synthesis of CeNC/IONC/MSN-T807-MB with bimodal imaging capability and its tau-targeted synergistic therapy. (E) *In vivo T*_1_-weighted MR images (left) and corresponding color-mapped images (right) of the brain of okadaic acid-treated rats at 6 h post-injection of CeNC/IONC/MSN-T807, CeNC/IONC/MSN, and saline. Reproduced with permission [117]. Copyright 2018, American Chemical Society. (For interpretation of the references to color in this figure legend, the reader is referred to the Web version of this article.)

### 4.4 Biomimetic nanoparticles

Biomimetic nanoparticles are novel bioactive materials that utilize or mimic natural biological processes and interactions *in vivo* for drug delivery with enhanced efficiency and precision [118]. Despite remarkable advances in the design and efficacy of traditional synthetic nanomaterials, biological and biomimetic nanocarriers exhibit higher biosafety and multiple functions. These nanoparticles generally include extracellular vesicles (EVs) and cell membrane-based nanoparticles.

EVs, once considered cell debris, are heterogeneous membranous particles secreted by various cells into extracellular areas. They can be categorized into exosomes, microvesicles, and apoptotic bodies based on their size and biogenesis. Growing evidence indicates that EVs play essential roles in various biological processes, including cell-cell communication, tissue development and maintenance, and immune response [119], [120], [121]. Among them, stem cell-derived EVs, particularly from mesenchymal stem cells (MSCs) and NSCs, have attracted considerable attention as promising therapeutic candidates for AD and other neurodegenerative disorders. These EVs carry a diverse cargo, including Aβ degradation-related enzymes and lipids, growth factors, neurotrophic factors, and therapeutic microRNAs, thus facilitating cognition improvement, Aβ/α-syn clearance, neuroprotection, and neural regeneration [122]. Previous studies have illustrated that MSC-EVs were of great significance in modulating multiple AD pathological processes. Specifically, they can enhance the expression of enzymes involved in Aβ degradation (e.g., neprilysin and insulin-degrading enzyme), suppress inducible nitric oxide synthase, and promote angiogenesis and neurogenesis. Additionally, MSC-EVs contribute to improved mitochondrial and synaptic function, as well as attenuating glial activation, neuroinflammation, and oxidative stress [123], [124], [125], [126]. Wang et al. reported that intravenous injection of MSC-EVs could facilitate the restoration of neuron morphology, neuron excitability, and mitochondrial alterations in APP/PS1 mice. These neuroprotective effects contributed to reduced Aβ deposition and attenuated neuronal loss in the hippocampus, which could be associated with the Nrf2 signaling pathway [127]. NSCs are endogenous stem cells residing in specific niches of the CNS that can differentiate into neurons, astrocytes, and oligodendrocytes, playing an important role in neurogenesis and neural repair [128]. Compared to MSC-EVs, NSC-EVs demonstrate enhanced efficacy in promoting neural functional recovery, likely due to their inheritance of neurogenic and neuro-regenerative potential from their parental cells [129]. Apart from using EVs as direct therapeutic agents, there is a recent increasing focus on their application as novel drug delivery vehicles. Compared to traditional synthetic nanoparticles, EVs with endogenous and membrane-like properties exhibit relatively lower immunogenicity, longer blood circulation, greater BBB penetrability, and higher bioavailability and biocompatibility. More importantly, evidence has demonstrated that EVs possess the property of chemotaxis and homing towards the site of neuropathology, significantly improving the targeted delivery efficiency of drugs to AD lesions [115]. In addition, targeting peptides, proteins, or antibody domains can be easily modified on the surface of EVs through genetic methods, click chemistry, and other approaches for more controllable and precise delivery [100]. Recently, Du et al. developed an engineered hybrid exosome system for co-delivering PDA and resveratrol, combining the intrinsic brain-homing effects of brain microvascular endothelial cell-derived exosomes with the innate inflammation-targeting abilities of macrophage-derived exosomes (Fig. 6A) [130]. Together with the surface-modified Aβ-targeting aptamer, this multi-targeted exosome efficiently crossed the BBB and accumulated at inflammatory sites after capturing Aβ, intervening in AD pathology through multiple pathways and remarkably restoring the memory deficits of APP/PS1 mice, providing a promising platform for multifaceted AD treatment.

**Fig. 6:**
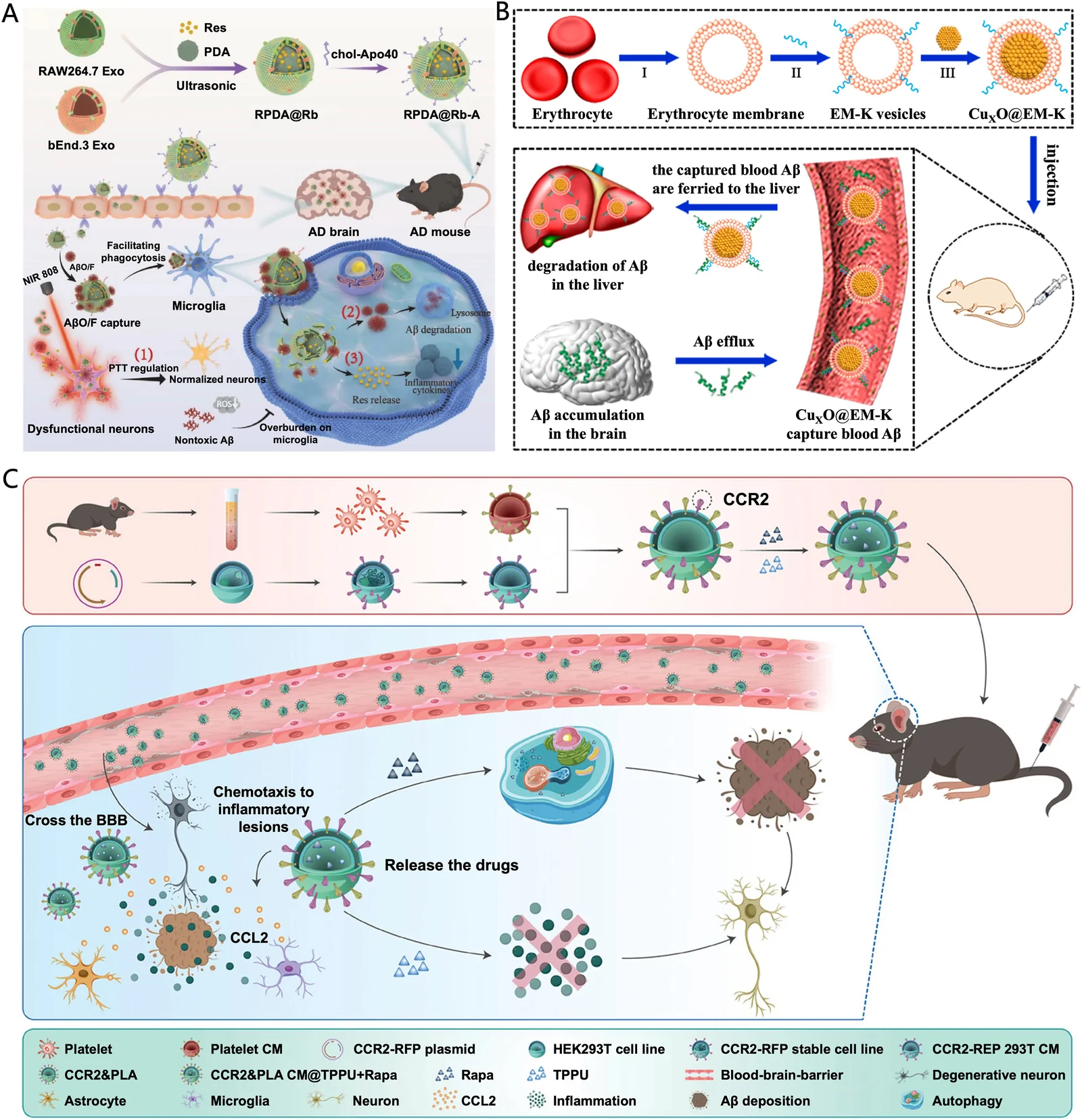
**Biomimetic nanoparticles for AD treatment.** (A) Scheme of the synthetic procedure of the multi-targeted hybrid exosome (RPDA@Rb-A) and its multi-mechanistic intervention in AD pathology. Reproduced with permission [130]. Copyright 2025, Elsevier. (B) Scheme of the synthetic procedure of erythrocyte membrane-coated nanoparticles (Cu_*x*_O@EM-K) and their therapeutic effects through peripheral Aβ clearance. Reproduced with permission [131]. Copyright 2020, American Chemical Society. (C) Scheme of the synthetic procedure of hybrid cell membrane liposomes and their application for neuroinflammation-targeted drug delivery in AD mice. Reproduced with permission [132]. Copyright 2024, John Wiley and Sons.

Cell membrane-based nano-delivery systems have emerged as promising carriers for AD therapy. By encapsulating drugs within the cell membrane, nanoparticles can mimic the natural biological process *in vivo*, which contributes to prolonged blood circulation, increased BBB penetration, reduced immunogenicity, and enhanced biocompatibility. In addition, the unique substances located at the cell membranes contribute to additional properties and biological functions of their source cells, including endothelial transport, selective focal adhesion, inflammatory site targeting, and regenerative capabilities [133]. A variety of cell types, such as red blood cells (RBCs), macrophages, microglia, platelets, and different stem cells, have been exploited to develop membrane-based nanocarriers for AD drug delivery. RBCs are the most abundant cellular components in blood. Nanoparticles coated with RBC membranes retain the CD47 transmembrane protein, a cell surface glycoprotein molecule that can bind to signal regulatory protein α on macrophages. This interaction transmits a “don't eat me” signal, effectively suppressing macrophage phagocytosis and enabling the nanoparticles to evade immune clearance, thus significantly prolonging their systemic circulation time [134]. Of note, evidence illustrated that RBC membranes could capture Aβ peptides in the blood via C3b-complement receptor 1 interaction and facilitate their transport to the liver for degradation, thus clearing peripheral Aβ, promoting a large efflux of brain-derived Aβ into peripheral circulation through the sink effect, and ultimately decreasing the Aβ levels in the brain [[135], [136]]. Based on this, Ma et al. reported a novel RBC membrane modified nanoparticle (Cu_x_O@EM-K) for peripheral AD treatment (Fig. 6B) [131]. As a result, Cu_x_O@EM-K significantly decreased the Aβ burden in both the bloodstream and the brain, contributing to the amelioration of cognitive functions of 3×Tg-AD mice. Microglia, the resident macrophages of the CNS, are naturally recruited into neuroinflammation-prone areas, playing an important part in AD pathogenesis and progression [137]. Thus, nanocarriers with microglia membrane coating could actively move to AD lesions due to inflammation-directed chemotaxis [138]. Wang et al. used BV2 cell membranes to coat PEGylated liposomes for the delivery of human fibroblast growth factor 21 (hFGF21) through subcutaneous injection. Due to the overexpressed α4/β1 integrin and vascular cell adhesion molecule-1 (VCAM-1) on the BV2 membrane, the hFGF21-loaded liposomes could specifically target Aβ-activated microglia through integrin-VCAM-1 interaction and alleviate Aβ-induced cognitive deficits by inhibiting neuroinflammation [139]. Apart from single-cell membranes, hybrid membranes integrating components from multiple cells have been developed to combine the biological functions of different cells for more precise and efficient drug delivery. Lin et al. proposed a novel delivery system by fusing platelet membranes with those of HEK293T cells transfected to overexpress chemokine (C-C motif) receptor 2 (CCR2) (Fig. 6C) [132]. In this system, platelet membranes served as transporters for enhanced BBB penetration and longer circulation duration, while CCR2‐transfected HEK293T cell membranes selectively targeted the CCL2 that was upregulated in neuroinflammatory regions, facilitating precise drug accumulation in AD lesions. Overall, hybrid membrane-based nanoparticles provide new opportunities to achieve precision drug delivery to the CNS and AD pathology [132].

### 4.5 Cargo-nanoparticle matching for precision drug delivery

Different nanoparticles possess distinct advantages and limitations that determine their suitability for different delivery requirements. A range of cross-platform parameters, such as particle size, surface charge, rigidity, degradability, ligand density, circulation time, and endosomal escape capacity, collectively influence nanoparticle behavior *in vivo*, including BBB transport, brain distribution, and targeting specificity [73]. These properties are also crucial for biosafety and translational feasibility. Table 1 provides a cross-platform comparison of these parameters across different nanoparticle types and summarizes their implications for AD drug delivery. Moreover, the selection of an appropriate nanocarrier should be guided by the physicochemical properties, structural stability, and site of action of the therapeutic cargo. Small-molecule drugs are compatible with polymeric and lipid-based nanoparticles, which can improve the solubility, pharmacokinetics, and stability of poorly soluble compounds. Peptides and proteins can be efficiently delivered by polymeric, lipid-based, or biomimetic nanoparticles, as these platforms can reduce proteolytic degradation and preserve biological activity [140]. Antibodies, due to their large molecular size and mainly extracellular targets in AD, do not necessarily require nanoparticle encapsulation and are more commonly conjugated to the surface of lipid-based, polymeric, inorganic nanoparticles, or EVs. Ionizable lipid nanoparticles are especially advantageous for nucleic-acid therapeutics because they efficiently encapsulate negatively charged nucleic acids, protect them from nuclease degradation, and promote endosomal escape and cytosolic release [141]. Cationic or pH-responsive polymeric nanoparticles and engineered extracellular vesicles provide alternative platforms for RNA delivery. For gene-editing cargos, ionizable lipid nanoparticles are particularly suitable for co-delivery of Cas9 mRNA and guide RNA, whereas polymeric or lipid-polymer hybrid systems have also shown considerable potential for delivery of preassembled Cas9 ribonucleoprotein complexes, owing to their ability to stabilize large protein-RNA cargos and facilitate cytosolic release [142]. In contrast, inorganic nanoparticles are generally less suitable for encapsulation of biomacromolecules but are particularly valuable when therapeutic delivery is integrated with material-intrinsic magnetic, catalytic, optical, imaging, or other externally controllable functions [143]. A comparative overview of different nanoparticle types in terms of preferred cargos, major advantages and limitations, and current clinical translation in AD is provided in Table 2. Notably, nanoparticles can be further engineered to accommodate distinct cargos and overcome specific barriers along the precision delivery cascade. Representative engineered nanoparticle-based delivery systems developed for AD therapy are summarized in Table 3.

**Table 1:** Comparison of NP design parameters and their implications for AD delivery.

Parameters	Polymeric NPs	Lipid-based NPs	Inorganic NPs	Biomimetic NPs	Design implications for AD delivery
Particle size	Highly tunable	Highly tunable	Precisely controllable	Heterogeneous	Regulates endothelial uptake, BBB transcytosis, clearance, and brain diffusion; oversized NPs impair BBB penetration and increase sequestration.
Surface charge	Highly tunable	Tunable; ionizable	Highly tunable by surface coatings	Usually inherit mildly negative	Near-neutral surfaces improve circulation; cationic surfaces enhance BBB uptake but cause non-specific binding and toxicity.
Rigidity	Tunable	Generally soft and deformable	Generally rigid	Soft and deformable	Maintains stability and ligand presentation for targeting; excessive stiffness limits endocytosis and brain diffusion; excessive softness risks premature drug release and safety.
Degradability	Highly degradable; degradation rate regulate release	Highly degradable	Variable/limited; prolonged tissue persistence	Highly degradable	Poor degradation increases long-term accumulation and toxicity; premature degradation reduces delivery efficiency.
Ligand density	Highly controllable by polymer conjugation chemistry	Highly controllable by functional lipids/PEG linkers	Highly controllable by surface conjugation chemistry	Less controllable	Optimal density improves BBB transport and brain targeting; excessive density causes endothelial retention, steric hindrance, or nonspecific binding.
Circulation	Prolonged by PEG/hydrophilic coatings	Prolonged by PEG/near-neutral ionizable formulations	Size- and coating-dependent	Immune-evasive/homing potential	Long circulation enhances BBB exposure but may cause immunogenicity, peripheral accumulation and off-target effects
Endosomal escape	High using pH-responsive, protonatable polymers	High, especially ionizable LNPs	Generally low	Variable and often insufficient	Essential for intracellular delivery; excessive escape causes endothelial retention or membrane toxicity; insufficient escape limits efficacy.
Protein corona	Moderate	Moderate	Notable due to high-energy surfaces	Limited	May mask ligands, altering BBB transport, brain distribution, immune clearance, and targeting specificity.

**Table 2:** Cargo compatibility, advantages, limitations, and clinical translation of representative NP platforms for AD.

NP type		Preferred cargo	Advantages	Limitations	AD clinical trials
Polymeric NPs	Polymersomes	Small molecules; peptides/proteins; antibodies	Dual hydrophilic/hydrophobic loading; sustained/controlled release	Potential protein activity loss; complex fabrication	None
	Dendrimers	siRNA; mRNA; plasmid DNA	Multivalent cargo complexation; precise surface functionalization; cellular uptake/endosomal escape	Cationic toxicity; complex synthesis; limited biodegradability	None
	Micelles	Hydrophobic small molecules	High solubilization capacity, simple self-assembly, small size	Premature disassembly; limited macromolecule loading	NanoLithium® NP03 (NCT05423522, completed): micelle-based lithium for mild-severe AD
Lipid-based NPs	Liposomes	Small molecules; peptides/proteins; antibodies	Dual hydrophilic/hydrophobic loading; biocompatibility; easy surface modification	Drug leakage; limited stability; weak endosomal escape	ACI-24.060 (NCT05462106, recruiting): liposomal anti-Aβ active immunotherapy for prodromal AD JNJ-64042056 (NCT06544616, active, not recruiting): liposomal anti-p-tau active immunotherapy for preclinical AD APH-1105 (NCT03806478, unknown): liposome for mild-moderate AD
	LNPs	siRNA; mRNA; ASO; gene-editing cargos	High RNA encapsulation; nuclease protection; cellular uptake and endosomal escape	Liver tropism; storage instability	ALN-APP (NCT05231785, recruiting): lipid-conjugated siRNA (not a conventional LNP) for early-onset AD
	SLNs/NLCs	Hydrophobic small molecules	High lipophilic-drug loading; improved solubility; sustained release	Limited macromolecule loading; lipid crystallization; drug expulsion	Longvida®curcumin (NCT01001637, terminated): SLN-based curcumin for moderate-severe AD/normal cognition
Inorganic NPs	MSNs	Small molecules; peptides/proteins; antibodies	High surface area and pore volume; high loading capacity	Poor biodegradability; long-term retention	None
	AuNPs	Imaging/photothermal agents	Easy surface conjugation; tunable optical properties; theranostic applications	Limited intrinsic cargo capacity; size-dependent; long-term retention	None
	Quantum dots	siRNA/oligonucleotides; imaging agents	Strong photostability; Tunable fluorescence; imaging-guided delivery	Heavy-metal toxicity; RES accumulation; long-term retention	None
	Magnetic NPs	Small molecules; siRNA; peptides/proteins; imaging agents	Magnetic guidance; MRI compatibility; externally controllable localization; theranostic potential	Aggregation; iron-related toxicity; long-term retention	None
Biomimetic NPs	Extracellular vesicles	siRNA/miRNA; peptides/proteins; small molecules	Intrinsic biocompatibility; BBB-crossing ability; biological homing; therapeutic effects	Source heterogeneity; limited drug loading efficiency; scale-up and standardization challenges; stability	NSC-EVs (NCT07554872, not yet recruiting): moderate-severe early-onset AD Adipose MSC-EVs (NCT04388982, unknown): mild-moderate AD
	Cell membrane-based NPs	Small molecules; nucleic acids; proteins	Membrane-mediated immune evasion and biological homing	Membrane heterogeneity; coating reproducibility; stability	None

**Table 3:** Various engineered NPs previously developed for the efficient delivery of AD drugs.

NP type	Drugs	Size (nm)	AD model/delivery route	Modification/Functionalization/Supplementary components	Refs.
PLGA	Fingolimod	99.5 ± 2.7	APP/PS1 mice Oral administration	Mannose ligand: hitchhike the GLUT1 circulation across the intestinal epithelial barrier and the BBB via CMT.	[88]
	Berberine	264.2 ± 44.7	i.c.v.-STZ-injected rats i.v.	Tet-1: high neuronal affinity to shunt the BBB.	[144]
	CeNP	143.6 ± 6.3	5×FAD mice i.v.	CKLVFFAED: target RAGE for BBB penetration and recognize intracerebral soluble Aβ.	[145]
	NAD^+^/Beclin1	58.8	APP/PS1 mice i.v.	Ang2: enhance the targeting and internalization toward the BBB via LRP-1.	[146]
	Necrostatin-1s	90 ± 15.2	APP/PS1 mice i.v.	CRT peptide: improve BBB penetration by targeting iron-importing protein complex of transferrin and TfR; CD47: prolong the circulating half-life of the drug.	[147]
	Curcumin	247.3 ± 0.6	C57BL/6 mice i.n.	Chitosan: enhance the permeability through the mucosa and delivery of curcumin to brain.	[148]
	Lutein	142.3 ± 2.57	Sprague Dawley rats i.n.	Chitosan: enhance mucoadhesion and mucosal permeation.	[149]
	Basic fibroblast growth factor	178.9 ± 11.7	i.c.v.-Aβ-injected mice i.n.	*Solanum tuberosum* lectin: selectively bind to N-acetylglucosamine on nasal epithelial membrane.	[150]
	Huperzine A	153.2 ± 13.7	Kunming mice i.n.	Chitosan: promote mucosal absorption; Lactoferrin: enhance nose-to-brain delivery.	[151]
	Curcumin	150	i.p.	B6 peptide: increase BBB permeability	[152]
	Vitamin D-binding protein	226.6 ± 44.4	5×FAD mice i.v.	PVA layer: increase the stability of colloidal suspension	[153]
PCL	Romidepsin/metformin	89.35 ± 6.23	i.c.v.-STZ-injected mice i.v.	Poloxamer: adjust the BBB penetrability	[154]
PMLA	miRNA-186	3.811 ± 1.58	5×FAD mice i.v.	D3 peptide: aid BBB crossing and specific targeting in the parenchyma via LRP-1.	[155]
PLA	H102/NAP	140	3×Tg-AD mice i.v.	H102: a β-sheet breaker acting on Aβ; NAP peptide: a microtubule stabilizer acting on p-tau CGN: BBB targeting ligand; Tet1 peptide: neuron-targeting ligand.	[156]
	NAP	170.7 ± 4.2	i.c.v.-Aβ-injected mice i.v.	TPL: fusion peptide comprising a BBB-penetrating peptide TGN and a neuron-binding peptide Tet1	[157]
PTMC	HNSS peptide	127.7 ± 0.9	3×Tg-AD mice i.v.	FGL peptide: bind to cholinergic neurons in the lesion site; HNSS: antioxidant peptide SS31 and neuroprotective peptide S14GHumanin;	[158]
PVP	Rhein/polydopamine	78	APP/PS1 mice i.v.	KLVFFAED: cross the BBB and target Aβ	[159]
Chitosan	Catechin	40-45	i.p.-AlCl_3_-injected rats Oral administration	Chitosan: improve their bioavailability by enhancing their intestinal absorption, stability, sustained release, and plasma concentration	[160]
	Resveratrol	19.60 ± 1.14	obesity-related AD mice i.g.	TGN: brain targeting peptide	[161]
	GQD	4-20	i.c.v.-STZ-injected rats i.n.	Chitosan: open the tight junctions of cells and exhibit intrinsic mucoadhesive features	[162]
	Oxytocin	122	APP/PS1 mice i.v.	Ang-2: increase the BBB crossing via LRP-1	[163]
PDMAEMA	BACE siRNA	70-80	APP/PS1 mice i.v.	CGN peptide: BBB penetration peptide; Tet1 peptide: neuron-specific binding.	[164]
PDA	PDA/KLVFF	90	FAD^4T^ mice i.v.	KLVFF: enhance pathological BBB crossing and lesion site accumulation via Aβ hitchhiking	[20]
	Hydroxychloroquine/all-trans retinoic acid	75.42	APP/PS1 mice i.v.	DAG: target astrocytes via connective tissue growth factor; KLVFFAED: target microglia via RAGE; TK-linker: enable ROS-sensitive drug release.	[165]
Dendrimer	CSF1R inhibitor (D-45113)	-	5×FAD mice i.p.	Hydroxyl dendrimer: cross the BBB and are preferentially phagocytosed by plaque-associated microglia (PAMs)	[91]
	Prussian blue	49 ± 15	APP/PS1 mice i.v.	Ang-2: increase the BBB crossing via LRP-1	[92]
	BACE1 siRNA/Rapamycin	129.6 ± 22.6	ICV-Aβ-injected mice i.n.	*Aleuria aurantia* lectin (AAL): bind with L-fucose residues expressed on the olfactory epithelium for effective transportation into the brain. KLVFF: recognize and bind to Aβ.	[166]
	LQLDEETGEFLPIQ	21	APP/PS1 mice i.v.	KLVFFAED: actively target the AD lesion area via RAGE	[89]
	BACE1-AS shRNA plasmids	110	APP/PS1 mice i.v.	D-TLKIVW: disrupt the p-tau associated fibril formation RVG29: a brain-targeting peptide	[167]
Micelle	Rapamycin	41.05 ± 1.20	3×Tg-AD mice i.v.	TPL: BBB-penetrating peptide TGN and GT1b ganglioside receptor-binding peptide Tet1	[90]
	Solanesol/chlorogenic acid	198.40 ± 18.87	APP/PS1 mice i.v.	CKLVFFAED: increase BBB permeability and Aβ targeting ability IR780: controlled drug release under irradiation with 808 nm laser	[168]
	Linoleic acid	126 ± 11.3	AlCl_3_-induced rats Oral administration	Lactoferrin: improve BBB permeability.	[169]
	Curcumin	65	APP/PS1 mice i.v.	KLVFFAED: mimic Aβ transport via RAGE-mediated targeting. (PEG)-LysB: ROS responsiveness	[170]
Liposome	TREM2 ASO/Resveratrol	123.42 ± 11.82	APP/PS1 mice i.v.	D-T7 (HRPYIAHC): increase BBB crossing via TfR; MG1 (CHHSSSAR): target M1 microglia.	[171]
	MiR-195	173.6 ± 1.16	APP/PS1 mice i.v.	Mannopyranoside/TAT: increase BBB crossing via GLUT1 and cell membrane-penetrating capability	[97]
	siROCK2/Lithospermic acid B	168.20 ± 2.28	3×Tg mice i.v.	TPL: BBB-penetrating peptide TGN and GT1b ganglioside receptor-binding peptide Tet1 for BBB and neuron-targeting	[172]
	Felodipine	131.10 ± 16.39	5×FAD mice i.v.	LIPUS irradiation: assist BBB-crossing by temporarily and reproducibly disrupting BBB integrity.	[173]
	hFGF21	98.91 ± 2.87	Aβ_1-42_-injected mice Subcutaneous injection	BV2 cell membrane coating: target Aβ-activated microglia via VCAM-1 and integrin α4/β1 interaction	[139]
	Ceria	95.6 ± 12.6	APP/PS1 mice i.n.	KLVFF: bind to Aβ via hydrogen-bonding interactions	[174]
	Insulin	137.9 ± 28.2	i.p.-STZ-injected rats i.n.	Chitosan: increase insulin permeability through the nasal mucosa	[175]
	Plasmid BDNF	178.6 ± 0.91	APP/PS1 mice i.v.	Mannose/Penetratin/RDP/CPP: enhance translocation across BBB	[176]
LNP	SSK1	166 ± 5	3×Tg mice i.v.	Neurotransmitter-derived synthetic lipid: improve BBB penetration capability	[177]
	α-mangostin/BACE1 siRNA	107.89 ± 1.34	APP/PS1 mice i.n.	Lactoferrin: improve brain-targeting efficacy through RMT	[98]
	MiR-17 antagomir	-	5×FAD mice Intracisternal-magna injection	Mannose modification: target to mannose receptors readily expressed on microglia	[104]
	Curcumin/SPION	180	APP/PS1 mice i.v.	CRT (CRTIGPSVC): increase BBB crossing via TfR QSH (QSHYRHISPAQV): bind to early Aβ plaques.	[178]
SLN	Rapamycin	150 ± 9	i.c.v.-Aβ_25-35_-injected mice	Transferrin: improve BBB permeability via TfR.	[179]
	Resveratrol	152.42 ± 0.37	APP/PS1 mice i.v.	RVG: improve brain targeting; Triphenylphosphine: improve mitochondria targeting; RBC coating: endow unique biological functions of RBCs.	[180]
	Curcumin	100.9 ± 1.4	Aβ and d-gal-injected rats i.v.	Lactoferrin modification: increase BBB permeability via RMT.	[181]
MSN	CeNP	110	APP/PS1 mice i.v.	1F12: specifically bind to soluble Aβ42 monomers, oligomers, protofibrils, and insoluble fibers and plaque; CeNP: scavenge ROS; RVG29: a brain-targeting peptide binding to nAChR	[182]
	Curcumin/CeO_2_	100	APP/PS1 mice i.v.	IR780: a photosensitizer for controlled drug release CKLVFFAED: recognize and bind to Aβ and inhibit Aβ aggregation	[183]
	Ceria	78.5	i.c.v.-OA-injected rats i.c.v.	AT8: specifically bind with phosphorylation sites (Ser202/Thr205) IONC: monitor drug delivery and disease treatment through MRI.	[184]
	Methylene blue/ceria	131.6	OA-injected rats intracranial injection	T807: bind to tau aggregates with high selectivity; IONCs: high-resolution MRI agents.	[117]
	Clioquinol	52.13	ICR mice i.v.	AuNP: decrease the Aβ self-assembly	[185]
MNP	siNF-κB/rosmarinic acid	205.6	APP/PS1 mice i.v.	RVG 29: improve BBB penetration capability via AChR of neurons	[186]
	CeO_2_	330	5×FAD mice Extracorporeal bloodstream circuit	Fe_3_O_4_: achieve facile magnetic separation; CeO_2_: scavenge ROS; Aβ antibody: capture Aβ peptides specifically.	[187]
	C_3_N_4_ nanodots	20-40	APP/PS1 mice i.v.	Fe_3_O_4_: magnetize to the targeted Aβ regions under extrinsic magnetic field; BTA conjugation: specifically binds to the β2 position of the Aβ fibers.	[188]
	Osmotin	90	Aβ_1-42_-treated mice i.v.	Dextran coating: enhance the blood circulation time and stabilize colloidal solutions	[189]
	D-TLKIVWC peptide	121.4 ± 0.7	PS19 mice i.v.	D-TLKIVWC: tau fibril-binding inhibitor MNP: improve BBB penetration capability	[190]
	Aβ antibody W20	28.7 ± 2.1	APP/PS1 mice i.v.	W20: Aβ oligomer‐specific scFv antibody; XD4: activate the class A scavenger receptor (SR-A) on the microglia and promote phagocytosis.	[191]
	Rutin	13	APP/PS1 mice i.v.	Congo red: specifically bind to amyloid plaques that have extensive β-sheet structures	[192]
	γ377-395 peptide	21 ± 3.5	rTg4510 tau-transgenic mice intracranial injection	fibrinogen-derived γ377-395 peptide: a specific inhibitor of microglial activation via the MAC-1 receptor	[193]
Black phosphorus	Methylene blue	291	OA-injected mice i.n.	Hydrogel: provide high nasal mucosal retention and controlled drug release.	[194]
	Methylene blue	200	OA-injected mice i.v.	NIR: increase BBB permeability through the photothermal effect of black phosphorus	[195]
Prussian Blue	Prussian Blue	163.5 ± 10.0	APP/PS1 mice i.v.	CKLVFFAED: improve the BBB penetration and Aβ-targeting abilities.	[196]
	Interleukin-3/PBMn	144.0 ± 0.3	OA-injected mice i.v.	Dopaminergic MES23.5 neuronal cell membranes coating: target microglia via VCAM-1 and integrin α4/β1 interaction	[197]
Manganese dioxide	Manganese dioxide	118	TgCRND8 mice i.v.	TP: BBB-crossing terpolymer; 4G8: anti-Aβ antibody	[198]
QD	C_3_N nanodots	4.5 ± 0.4	APP/PS1 mice i.p.	C_3_N: inhibit Aβ aggregation and disaggregate mature Aβ fibrils	[199]
	Carbon dots	5	5×FAD mice i.c.v.	Aβ-binding DNA aptamers: bind to Aβ species	[200]
AuNP	AuNP	25.3 ± 2.55	-	PDA coating: improve the stability and biocompatibility of AuNPs Aβ_1–40_ aptamer: targeting Aβ_1–40_ peptides	[201]
	CeO_2_	100	APP/PS1 mice i.v.	KLVFF: recognize and bind the full length Aβ monomer sequence; NIR: improve the redox performance of ceria and BBB permeability.	[19]
	KLVFF/polyoxometalates	33	S4880202 mice i.v.	NIR: promote the dissociation of Aβ fibrils	[202]
	AuNP/Glutathione	9/15	APP/PS1 mice i.v.	Glutathione coating: increase BBB permeability via high levels of glutathione transporters located in the brain.	[203]
SeNP	SeNPs	101 ± 30	i.c.v.-STZ-injected rats i.v.	Amphipathic dipeptide: stabilize the NPs.	[204]
	Resveratrol	14 ± 0.12	AlCl_3_ with D-gal-induced mice Oral administration	TGN: BBB transport-targeting peptide	[205]
	SeQD	2-20	Aβ_1-42_ injected mice i.v.	SeQDs: ultrasmall size enabling quickly BBB penetration	[206]
RuNP	NGF	80	APP/PS1 mice i.v.	Se-Se-bond: break in the higher ROS and release NGF; RVG: improve the BBB penetration	[207]
PdNP	PdNPs	15.6	3×Tg-AD mice i.v.	Borneol-modification: a powerful BBB-opening effect by affecting neurotransmitters	[208]
CeNP	CeO_2_	22	5×FAD mice i.c.v.	TPP: a lipophilic cation targeting mitochondria	[209]
	Resveratrol	120	5×FAD mice i.v.	Lactoferrin modification: modulate BBB crossing and drug release rate and improve the biocompatibility.	[210]
Macrophage membrane	MoS_2_ QDs	125.62 ± 4.132	APP/PS1 mice i.v.	Macrophage Membrane: active targeting inflammation function NIR irradiation could actively increase the cross of BBB	[211]
	CQD	51.16 ± 2.13	APP/PS1 mice i.v.	CQD: capture excess Cu^2+^ and inhibit rapid Aβ aggregation NIR: dissolve fiber precipitates and improve BBB permeability	[212]
Hybrid membrane	Rapamycin/TPPU	100	5×FAD mice i.v.	CCR2 overexpressed cell hybrid membrane: cross the BBB and target neuroinflammatory lesions.	[132]
RBC membrane	PDA/CQD	169.9 ± 2.9	APP/PS1 mice i.v.	RBC: anti-immunorecognition properties to evade immune clearance NIR: dismantle formed Aβ fibers and enhance BBB permeability.	[213]
	Cu_x_O nanozyme	165.3 ± 3.9	3×Tg-AD mice i.v.	KLVFF: Aβ-specific ligand to selectively capture Aβ in the blood	[131]
Exosome	PDA/resveratrol	117.37 ± 4.58	APP/PS1 mice i.v.	bEnd.3 exosome: natural brain homing ability; RAW264.7 exosome: natural inflammation tropism; Apt40: nucleic acid Aβ40 aptamer	[130]
	BACE1 siRNA/TREM2 plasmid	134 ± 2.67	APP/PS1 mice i.v.	BACE siRNA: reduce the production of Aβ plaques; TREM2 plasmid: reprogram microglia from M1 to M2 phenotype; Exosome: provide homing ability and migrate to the lesion site; Ang-2: increase the BBB crossing via LRP-1	[99]
	BACE1 siRNA/caspase-3 siRNA	155.5	3×Tg mice i.n.	RVG 29: specifically bind to nAChR of neurons. BAP: achieve the controlled release in the high-ROS environment.	[214]

## 5 First target: targeting the BBB

Precision drug delivery for AD therapy typically progresses through two sequential stages. Firstly, administered nanoparticles circulate to the cerebral vasculature and penetrate the BBB via transcellular or paracellular transport pathways. Subsequently, they migrate to specific areas or cells associated with AD neuropathology in the brain, where they release the drugs and exert therapeutic effects at disease sites [75]. To achieve precision drug delivery with high efficiency, it is crucial to enhance the specific targetability of nanoparticles at two different levels: first, toward the BBB for effective entry, and second, toward AD-specific pathologically affected regions.

Generally, there are six ways by which substances penetrate the BBB under physiological conditions, including transcellular diffusion, paracellular transport, carrier-mediated transport (CMT), receptor-mediated transcytosis (RMT), adsorptive-mediated transcytosis (AMT), and cell-mediated transport (Fig. 7A and B) [[230], [231]]. Various nanoparticle systems have been strategically designed to enhance their BBB permeability through pathways as previously mentioned. The targeting specificity, advantages, limitations, cargo compatibility, and translational maturity of these BBB-targeting strategies are comparatively summarized in Table 4.

**Fig. 7:**
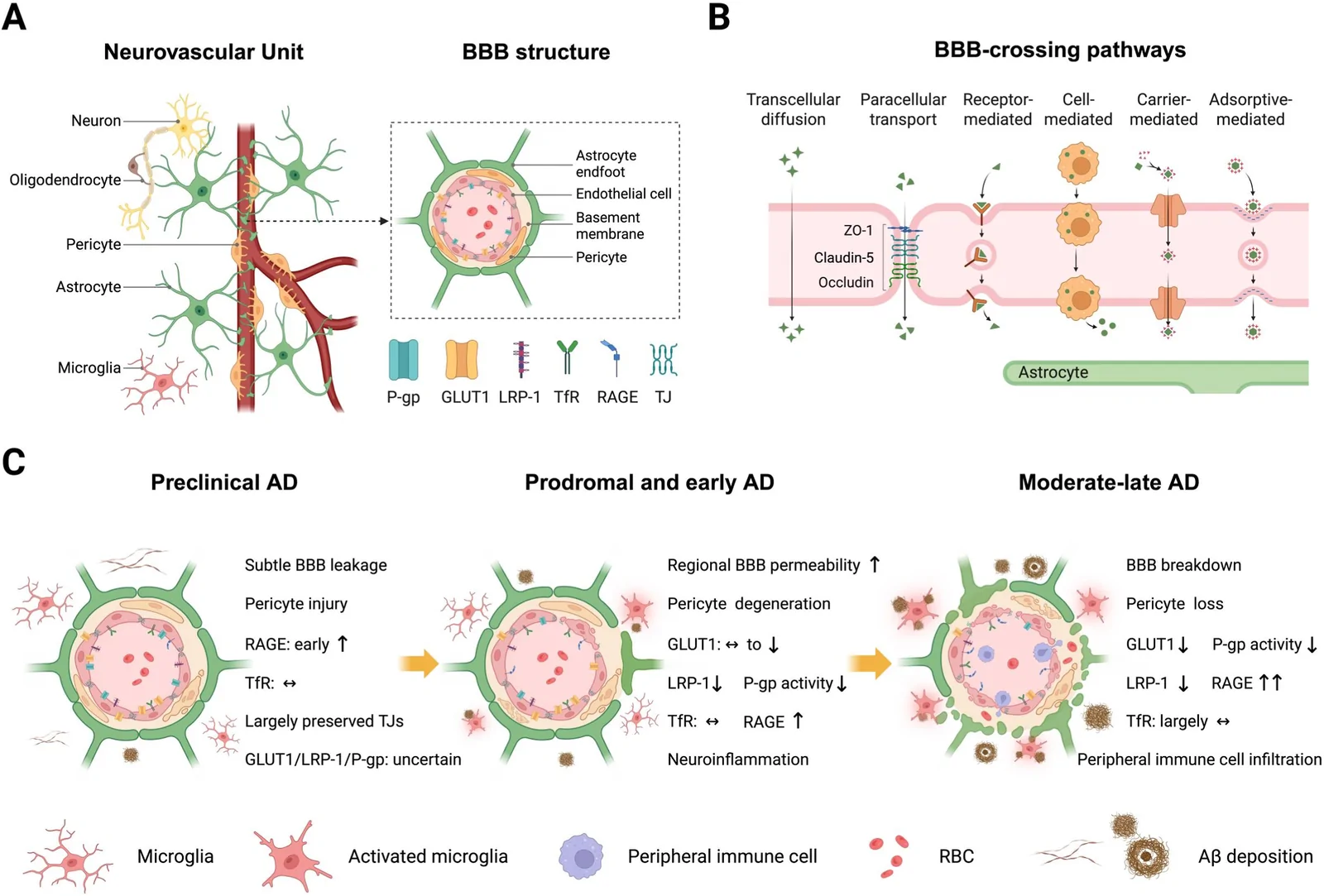
**BBB structure and alterations across AD progression.** (A) Structure of the neurovascular unit and BBB. (B) Schematic illustration of different pathways for BBB crossing. (C) Dynamic BBB alterations across different stages of AD. Created in BioRender. Shan, J. (2026) https://BioRender.com/izpt7z5.

**Table 4:** Strategies for BBB-crossing and their advantages, limitations, suitable cargo, and clinical translation.

Strategies	Material/Technique	Specificity	Advantages	Limitations	Suitable cargo	Clinical translation
Externally assisted strategies	Focused ultrasound	Spatially localized BBB opening at sonicated regions	High spatial and temporal precision; controllable and reversible BBB opening	Low molecular specificity; equipment dependence; repeat-dose vascular safety	Broad cargo compatibility	Early clinical/clinical proof-of-concept in AD
	Magnetic guidance	Magnetic field-directed regional accumulation	External spatial control; compatible with MRI; controllable release	Depth-limited targeting; RES uptake; aggregation; heating injury; repeat-dose-related cumulative iron toxicity	Broad cargo compatibility	Preclinical
	Laser/photothermal technique	Light-triggered localized BBB opening	High spatial resolution; controllable and reversible BBB opening; imaging and photothermal therapy	Limited transcranial penetration; thermal injury; repeat-dose safety	Broad cargo compatibility	Preclinical
Carrier-mediated transport	Mannose/Glucose [[88], [97], [215]]	GLUT1	Non-disruptive; physiologically and repeated dose compatible; molecular specificity; rapid transport; high transport capacity	Strict substrate requirements; transporter saturation/competition; disease-dependent transporter expression	Small molecules, transporter-mimetic prodrugs	Clinical validation: LAT1-L-DOPA; glutathione- PEGylated liposome (none for AD)
	Valine, Phe-Phe dipeptide, phenylalanine [[216], [217], [218]]	LAT1				
Receptor-mediated Transcytosis	Transferrin, CRT, B6, D-T7 [[152], [169], [171], [178], [219]]	TfR	High molecular specificity; non-disruptive; compatible with macromolecules, NPs, and repeated dosing; adaptable to diverse ligands and carriers; amenable to dual/cascade targeting	Saturable; peripheral receptor sequestration; endothelial sequestration or lysosomal routing due to excessive avidity; altered receptor abundance in AD	Antibodies, peptides/proteins, oligonucleotides, and ligand-functionalized NPs	Trontinemab: TfR1 brain-shuttle module coupled anti-Aβ antibody in two phase 3 clinical trials in MCI to mild AD dementia
	Ang-2, K16ApoE [[92], [146], [163], [220], [221], [222]]	LRP-1				
	HIRMAb [223]	InsR				
	RVG29 [[168], [196]]	nAChR				
	Lactoferrin [[151], [181], [210]]	LfR				
	DAG [224]	CTGF				
	RAP [225]	RAGE				
Adsorptive-mediated transcytosis	Cationic proteins, peptides (CPP), or NP surfaces [[97], [226]]	Negatively charged endothelial membranes	Receptor-independent; relatively simple surface engineering; high endothelial uptake	Poor molecular specificity; nonspecific peripheral interaction and uptake; cationic toxicity; repeat-dose safety	Cationic drugs and positively charged NPs	TAT-linked peptide nerinetide reached large phase 3 stroke trials (none for AD)
Cell-mediated transport	Monocytes, macrophages, neutrophils, lymphocytes, stem cells [[227], [228], [229]]	Inflammation/lesion-directed delivery; tissue-specific homing	Natural inflammatory, tissue-specific and lesion-homing; BBB crossing at diseased sites; protect cargo from systemic clearance; therapeutic potential of stem cells	Inflammation-dependent; slow/variable trafficking; difficult control of cellular phenotype, viability, and cargo loading and release; complex manufacturing; inflammatory effects; repeat-dose difficulty	NPs, proteins, other macromolecular cargos	Preclinical

### 5.1 Transcellular and paracellular transport

Under physiological conditions, transcellular diffusion is strictly limited to small gaseous molecules and lipophilic substances with molecular weights below 400-600 Da and fewer than 9-10 hydrogen bonds [[16], [230]]. Meanwhile, paracellular permeability of BMECs is virtually non-existent because of tight junctions, which physically block the paracellular transport of almost all drugs across the BBB. However, BBB integrity may be compromised under AD pathology [230]. Evidence suggests that Aβ or tau deposition surrounding the blood vessels may induce vascular damage, disrupt tight junctions, and alter the morphology and function of endothelia, pericytes, and astrocytes. These changes can further result in increased BBB permeability, which, nevertheless, also provides more opportunity for brain-targeted drug delivery [[232], [233], [234]].

To enhance brain delivery efficiency via transcellular and paracellular transport, nanoparticles can be lipidized or engineered to induce transient BBB opening. Growing evidence indicates that lipid-based nanoparticles can facilitate BBB transport due to their membrane-compatible lipid composition [118]. Meanwhile, various agents that can induce temporary BBB opening can be co-loaded with the therapeutic drugs. Several pharmaceutical compounds with this function have been used, such as zonula occludens (ZO) toxin, chitosan, borneol, histamine, bradykinin, oleic acid, and lysophosphatidic acid [[148], [162], [208], [230], [235]]. For instance, chitosan, a natural polysaccharide, can reversibly open tight junctions of endothelial cells to increase BBB permeability and has been widely incorporated into nanocarrier systems for AD therapy [[148], [162]]. Jia et al. functionalized their palladium nanozyme platform with borneol, an ingredient derived from Chinese traditional medicine that can open the BBB by modulating neurotransmitters, significantly increasing the palladium accumulation in the brains of 3×Tg-AD mice [208].

Exogenous effects, including focused ultrasound, magnetic field force, and laser, can also be exploited for CNS-targeted delivery by opening the BBB. Focused ultrasound, as a non-invasive technique, can achieve temporary and reproducible disruption of BBB integrity through different physical effects within the vasculature, creating localized windows for drug delivery to the brain parenchyma [[236], [237]]. Microbubbles are usually co-injected, which will rapidly collapse under acoustic pressure after arriving at the targeted area, generating shear forces and circumferential stresses to perforate the endothelial membrane and leading to transient BBB-opening with sub-millimeter accuracy [[173], [238]]. Both safety and efficacy of ultrasound-induced BBB-opening for AD treatment have been demonstrated in many studies, with BBB integrity typically restored within 4-6 h and few side effects (mainly headaches) reported [[173], [239], [240]]. He et al. developed a multifunctional delivery system (felodipine@LND) for 5×FAD mice therapy, applying a transcranial low-intensity pulsed ultrasound (LIPUS) irradiation to assist BBB crossing (Fig. 8A) [173]. Results showed that LIPUS opened the BBB reversibly and effectively promoted more felodipine@LND accumulation in the brain parenchyma than the sham groups. Histological analysis confirmed preserved vascular endothelial integrity, negligible neuronal degeneration, and no extravasation of erythrocytes after injection, indicating the feasibility and safety of ultrasound-assisted drug delivery (Fig. 8B). Magnetic control provides another non-invasive strategy for precision CNS drug delivery in AD therapy. Magnetic field force has been reported to possess the ability to reversibly interrupt the endothelial tight junctions and enhance the BBB permeability, which is potentially associated with the temporary disturbance of ZO-1 and magnetic hyperthermia induced by magnetic stimulus [[241], [242]]. Moreover, laser stimulation can exhibit similar effects. The paracellular tight junctions are temporarily disrupted by the local biophysical effects of picosecond laser pulses, thereby activating the paracellular transport pathway and increasing drug accumulation in specific areas [243]. Recently, Li et al. reported a feasible nanotechnology that could modulate BBB opening through transcranial picosecond laser stimulation of tight junction-targeting plasmonic AuNPs (Fig. 8C) [243]. The enhanced BBB permeability triggered by localized AuNP-laser pulse interaction effectively facilitated the delivery of antibodies, adeno-associated viral vectors, and liposomes to the CNS, with no evidence of overt impairment on cerebral vascular dynamics and brain parenchyma (Fig. 8D–F). Similarly, Ge et al. synthesized gold nanorod-based nanocomposites that could generate controlled photothermal effects under 808 nm near-infrared laser for safe BBB opening and efficient CNS-targeted delivery in AD mice, suggesting laser-mediated nanotechnology as a feasible avenue for therapeutic agent delivery in AD and other neurological disorders [19].

**Fig. 8:**
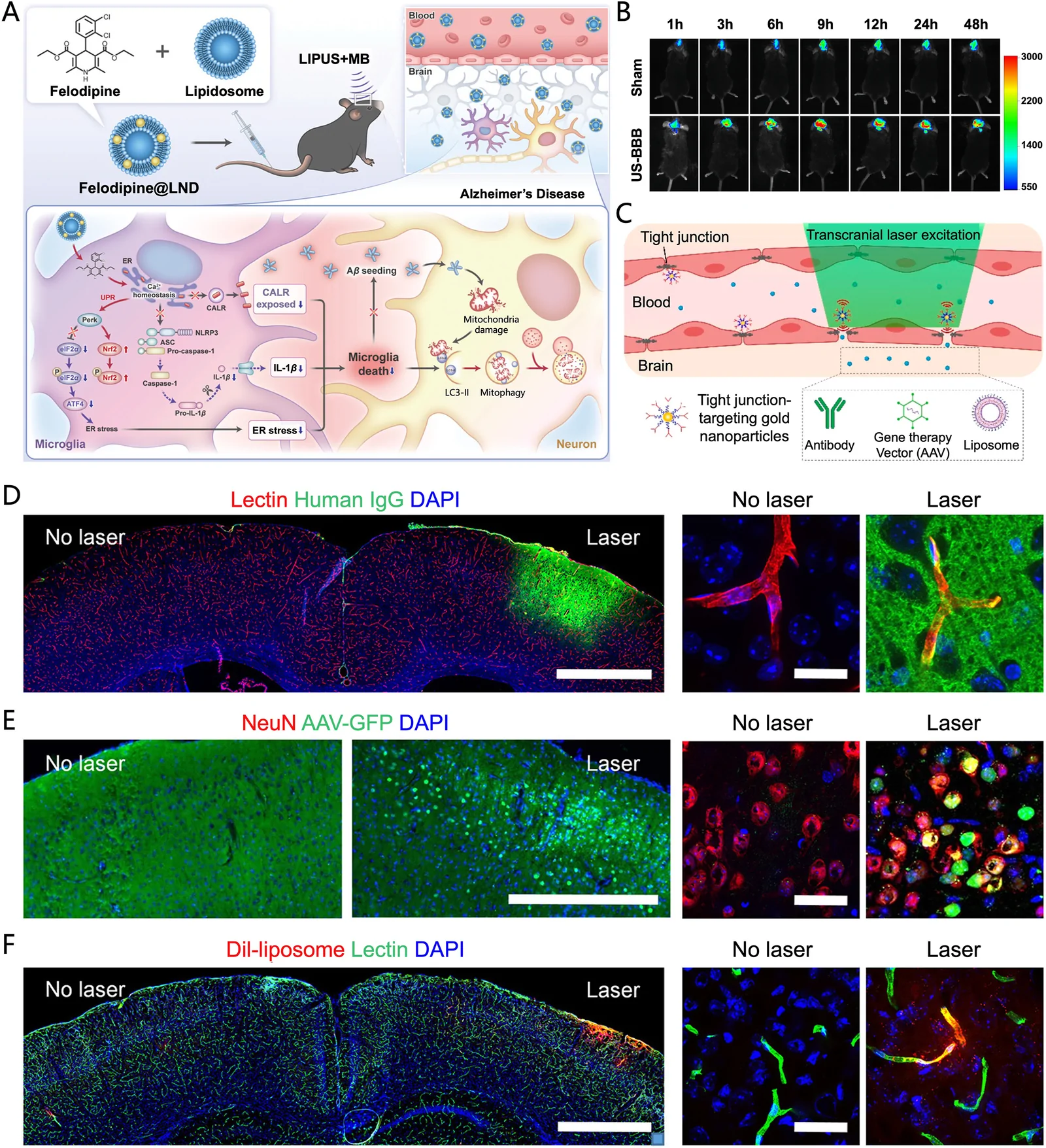
**BBB manipulation through exogenous effects for brain-targeted drug delivery.** (A) Scheme of LIPUS-mediated delivery of felodipine nanodrug (felodipine@LND) and its mechanism for BBB penetration to alleviate the anxiety‐like behavior and cognitive deficits in 5×FAD mice. (B) *In vivo* fluorescence imaging of the brains from mice with or without ultrasound after intravenous injection of DiR-labeled felodipine@LNDs. Reproduced with permission [173]. Copyright 2024, John Wiley and Sons. (C) Schematic illustration of transcranial laser excitation of tight junction-targeting AuNPs to induce transient BBB opening. (D-F) Representative fluorescence images of human IgG antibodies (D), adeno-associated viral vectors (E), and liposomes (F) delivered into the brain with or without laser-assisted BBB modulation. Reproduced with permission [243]. Copyright 2021, American Chemical Society.

### 5.2 Carrier-mediated transport

CMT refers to the translocation of specific molecules from one side of the endothelial cell membranes to the other side via the protein carriers or transporters expressed on the BBB. In brief, specific substances, such as glucose, amino acids, and nucleosides, bind to their corresponding transporters on the luminal side, triggering the conformation of the proteins and contributing to the transfer of molecules to the abluminal side, along or against the concentration gradient [244]. Several transporters involved in nutrient and metabolite transport are highly expressed at the BBB, including GLUT1, L-type amino acid transporter, adenosine transporter, and monocarboxylic acid transporter. Among these, GLUT1 is one of the most highly expressed transporters at the BBB and has been widely exploited for facilitating brain drug delivery via CMT [230]. Accordingly, nanoparticles can be engineered with substrate-mimicking moieties that are recognized by these endogenous transport systems, thereby promoting their transport across the BBB. For example, Zhou et al. suggested a “Trojan horse” strategy to deliver BACE1 siRNA in AD mice, functionalizing nanoparticles with D-galactose for more effective BBB-crossing via GLUT1 cycling (Fig. 9A) [245]. Specifically, luminal GLUT1 expression was initially elevated by hypoglycemia, and then recycled to the abluminal side upon glucose replenishment, thereby facilitating significantly increased delivery of glycosylated siRNA nanomedicine across the BBB. As illustrated in Fig. 9B, this strategy led to a 5.8 times higher brain accumulation than non-galactose-decorated nanoparticles. Similarly, Lei et al. designed a brain-targeted PLGA nano-delivery system externally grafted with α-mannopyranoside, exploiting the glycemia-controlled GLUT1-mediated transport for efficient BBB penetration and brain accumulation of these mannose-integrated nanoparticles in APP/PS1 mice [88].

**Fig. 9:**
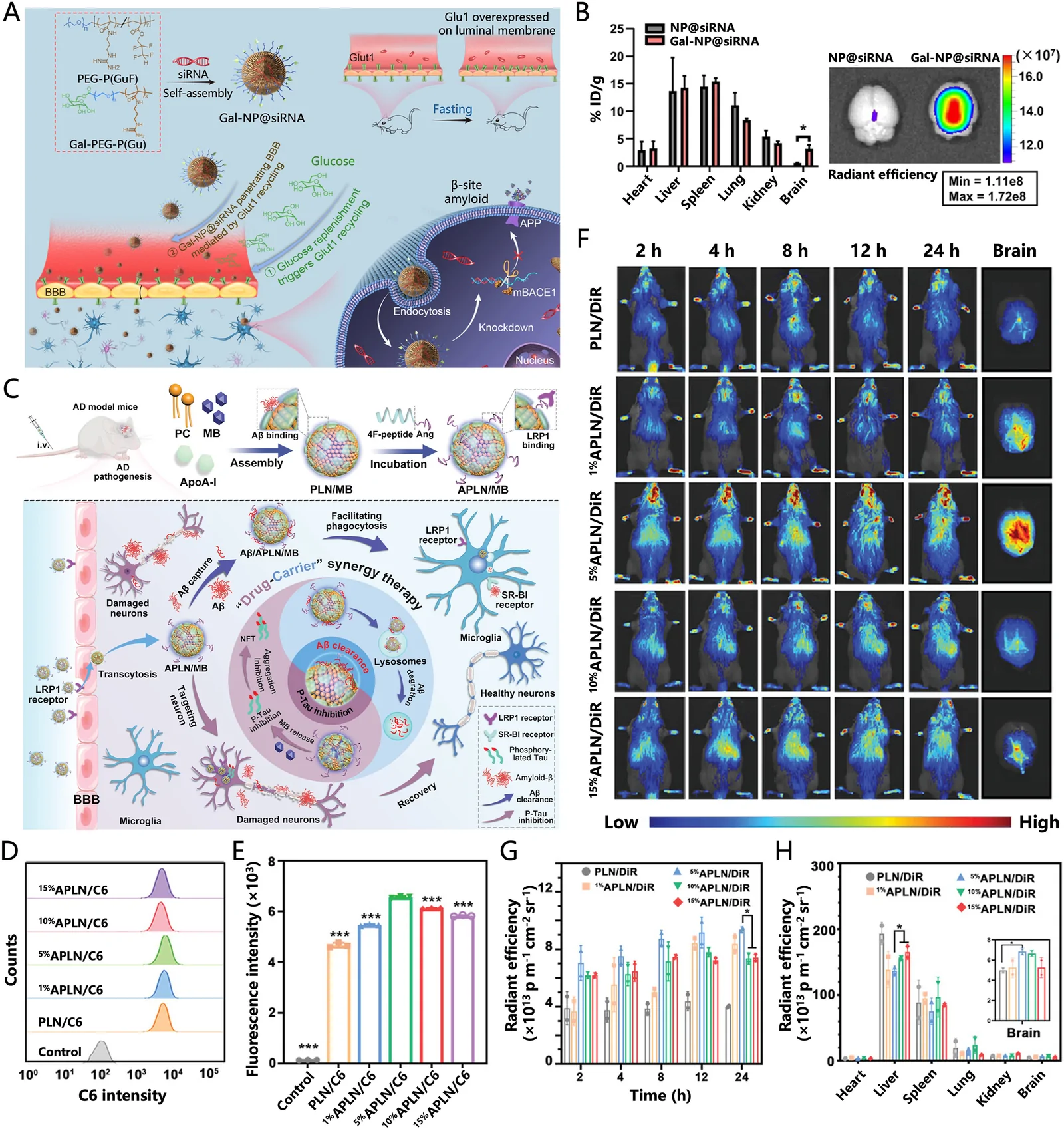
**Carrier- or receptor-mediated BBB transport for brain-targeted drug delivery.** (A) Schematic illustration of the synthesis of the glycosylated “triple-interaction”-stabilized siRNA nanomedicine (Gal-NP@siRNA) and its therapeutic mechanism against AD pathology in APP/PS1 mice. (B) Quantitative analysis of Cy5-siRNA accumulation in major organs and representative fluorescence images of whole brains from NP@siRNA and Gal-NP@siRNA treated mice at 1 h post-intravenous administration. Reproduced with permission [245]. Copyright 2020, American Association for the Advancement of Science. (C) Schematic illustration of the synthetic procedure of APLN/MB and its mechanism of “Drug‐Carrier” synergistic therapy in AD model mice. (D-E) Flow cytometry analysis of the uptake efficiency of PLN/C6 with different Ang densities (1%, 5%, 10%, and 15%) by hCMEC/D3 cells. (F) *In vivo* fluorescence images of mice treated with PLN/DiR with different Ang densities at different time points and *ex vivo* brain fluorescence images at 24 h post-intravenous administration. (G-H) Quantification of fluorescence intensity of brains at different time points and major organs. Reproduced with permission [246]. Copyright 2022, John Wiley and Sons.

### 5.3 Adsorptive-mediated transport

AMT involves the endocytic internalization of substances via electrostatic interactions between cationic substances and the anionic surface of the BBB [247]. The luminal plasma membrane of the BBB is rich in anionic molecules, such as proteoglycans, sialoglycoproteins, and sialoglycolipids, leading to a highly negatively charged surface [248]. Therefore, cationization of nanoparticles, either by introducing positive surface charges into nanoparticles or conjugating the nanoparticles with cationic moieties, represents a promising strategy to facilitate BBB penetration via AMT [230]. Positively charged nanoparticles have been demonstrated to exhibit higher efficiency in BBB penetration and brain accumulation compared to their neutral or anionic counterparts in many studies [[166], [167]]. In addition, various cell-penetrating peptides, which are typically short cationic or amphipathic peptides, are increasingly being applied to promote AMT. For example, the trans-activator of transcription (TAT) peptide has been identified as one of the most efficient sequences, significantly increasing the intracellular transport and brain accumulation of nanoparticles [[226], [249]]. Feng et al. constructed TAT-modified chondroitin sulfate-coated AuNPs (TAT-CS@Au) for AD therapy, achieving efficient internalization by SH-SY5Y cells through TAT-mediated endocytosis [249]. Arora et al. employed penetratin, another widely used cell-penetrating peptide, to functionalize liposomes for the delivery of a plasmid encoding brain-derived neurotrophic factor (BDNF) in APP/PS1 mice [176]. In this system, penetratin facilitated adsorptive membrane interaction and translocation, whereas mannose was additionally introduced to exploit GLUT1-mediated CMT for BBB crossing. The dual-functionalized penetratin-mannose liposomes achieved greater cerebral BDNF expression than liposomes modified with either penetratin or mannose alone, with an approximately 95% increase relative to PBS-treated APP/PS1 mice, highlighting the potential of integrating CPP-assisted AMT with GLUT1-mediated CMT for AD drug delivery.

### 5.4 Receptor-mediated transcytosis

RMT represents the primary pathway for the endocytic uptake of large molecules. Typically, the process begins with specific binding of molecular ligands to the corresponding receptors on the endothelial surface, triggering the subsequent membrane invagination, followed by clathrin-mediated or caveolin-mediated endocytosis, intracellular trafficking, and vesicular sorting, and culminating in abluminal endothelial membrane fusion and discharge of cargoes to the brain parenchyma [250]. Nanoparticles can be functionalized with versatile targeting moieties to exploit this natural transport mechanism. A variety of receptors, such as transferrin receptors (TfRs), low-density lipoprotein receptor, nicotinic AChRs (nAChRs), insulin receptors, and folate receptors, are highly expressed on the BBB surface, making RMT a feasible and promising strategy for efficient BBB transport [[230], [242]]. Among these, TfR is the most widely exploited target for RMT. Numerous studies have confirmed that conjugating various types of TfR ligands, such as transferrin, CRT peptide (CRTIGPSVC), and B6 peptide (CGHKAKGPRK), can significantly promote BBB crossing and increase cerebral drug accumulation [[251], [252]]. Ang-2, a 19-amino acid peptide, exhibits high binding affinity to LRP-1, which is highly expressed on the BBB endothelial cells and neurons. Various Ang-2-decorated nanoparticles have been applied for CNS-targeted delivery in AD therapy. Han et al. designed a biomimetic Ang-functionalized protein-lipid nanocomposite that could simultaneously target Aβ and tau-related pathways (Fig. 9C). With the help of LRP-1-mediated BBB transport, the Ang(5 wt%)-modified nanoparticles exhibited a 1.51-fold higher cellular uptake by hCMEC/D3 *in vitro* (Fig. 9D–E) and a 2.15-fold higher drug intracerebral accumulation *in vivo* (Fig. 9F–H) than Ang-absent-modified nanoparticles, contributing to significantly better therapeutic effects in neuroprotection and alleviation of cognitive impairment in AD mice [246]. Notably, in addition to receptors constitutively expressed at the BBB, several AD-associated molecular targets, such as connective tissue growth factor (CTGF) and RAGE, are also reported to be upregulated within the NVU, offering new opportunities for more disease-selective RMT. Lei et al. developed a multifunctional nanocleaner modified with the cyclic DAG peptide (CDAGRKQKC), which can recognize CTGF enriched in neurovascular endothelial cells and reactive astrocytes in AD lesions [224]. Results demonstrated that DAG could promote preferential binding, internalization, and subsequent transcytosis of nanoparticles at the diseased BBB, leading to a 1.76-fold higher brain fluorescence intensity than non-DAG-modified nanoparticles. Similarly, RAP peptide (CELKVLMEKEL) can recognize RAGE, which is highly expressed on the diseased NVU in AD, and promote BBB crossing and lesion accumulation via RMT [225]. He et al. constructed a RAP-functionalized co-delivery system (Ibu&FK@RNPs) for ibuprofen and FK506. Compared with non-targeted nanoparticles, this system exhibited 1.42-fold higher uptake by Aβ-stimulated brain endothelial cells and 1.55-fold higher brain accumulation in APP/PS1 mice. After crossing the BBB, the nanoparticles further targeted RAGE-expressing cells within AD lesions and released ibuprofen and FK506, resulting in attenuated neuroinflammation and improved cognitive function in AD mice. These studies illustrate that exploiting disease-associated receptor alterations at the BBB may extend conventional RMT from general brain targeting toward lesion-selective and cascaded precision delivery in AD.

### 5.5 Cell-mediated transport

Cell-mediated transport is an emerging “Trojan horse” strategy that exploits the intrinsic migratory and homing properties of circulating cells to deliver therapeutic cargos across the BBB [253]. This process relies on the transendothelial migration of carrier cells through paracellular or transcellular diapedesis. In neuroinflammation-related diseases, chemokine signaling and upregulated endothelial adhesion molecules promote the recruitment of circulating leukocytes, including monocytes, macrophages, and neutrophils. Most commonly, leukocytes adhere to and crawl along the endothelium, then migrate between adjacent endothelial cells through transiently opened intercellular junctions, thereby crossing the BBB via the paracellular pathway. In a minority of cases, transcellular diapedesis may occur, in which adhesion molecule-mediated endothelial cytoskeletal remodeling enables leukocytes to migrate directly through the endothelial cell body [254]. For drug delivery, nanoparticles can be internalized by or attached to immune cells, which subsequently act as “Trojan horses” to protect and ferry therapeutic cargos across the BBB and toward inflamed brain lesions [227]. As NVU dysfunction and neuroinflammation play critical roles in AD pathogenesis and progression, cell-mediated transport represents a feasible and promising strategy for brain-targeted drug delivery. In addition to immune cells, neural cells, particularly NSCs, have attracted increasing attention due to their intrinsic brain tropism, which enables them to migrate toward the CNS and preferentially accumulate at sites of cerebral injury [255]. This inherent homing capacity makes NSCs promising carriers for cell-mediated brain-targeted delivery.

### 5.6 Dynamic BBB alterations in AD and implications for BBB-crossing strategies

Notably, the BBB is not static during AD progression but undergoes dynamic remodeling that can affect drug delivery efficiency and safety (Fig. 7C and Table 5). In preclinical and early-stage disease, vascular abnormalities may precede overt cognitive impairment, including early pericyte injury, subtle and regional leakage, basement-membrane remodeling, and impaired perivascular and glymphatic clearance [[54], [256]]. As AD progresses, BBB exhibits increased permeability, progressive pericyte degeneration, deteriorating neurovascular coupling and cerebral perfusion, and gradual loss of perivascular AQP4 polarization in astrocytic endfeet [257]. Meanwhile, BBB transport systems are remodeled. Endothelial RAGE is generally increased, whereas GLUT1, LRP-1 and P-gp expression or activity is reduced, impairing nutrient transport and brain-to-blood Aβ clearance while enhancing RAGE-mediated influx of circulating Aβ [[258], [259]]. Accumulated Aβ activates oxidative and inflammatory signaling, and disrupts endothelial tight junctions, further compromising BBB integrity [260]. At more advanced stages, these abnormalities converge into widespread NVU dysfunction, characterized by endothelial mitochondrial dysfunction, tight-junction disruption, substantial pericyte loss, basement-membrane thickening and splitting, astrocytic endfoot retraction, and cerebral amyloid angiopathy [261].

**Table 5:** Dynamic BBB alterations across AD progression and their implications for nanomedicine delivery.

BBB alterations	Preclinical AD	Prodromal and early AD	Moderate-late AD	Implications for nanomedicine delivery
Endothelial cells and vascular function	Early neurovascular dysfunction	Endothelial barrier dysfunction; regional hypoperfusion; impaired neurovascular coupling and vascular Aβ clearance	Marked endothelial dysfunction with ultrastructural damage; CAA and cerebral microbleeds; hypoperfusion	Alter transcellular transport, CMT and RMT efficiency; hypoperfusion reduces vascular exposure and limits systemic NP delivery
TJs and paracellular permeability	Largely preserved TJs; subtle BBB leakage	Progressive TJ abnormalities; regional and heterogeneous increase in permeability	Extensive TJ disruption, vascular leakage, and BBB breakdown	May increase nonspecific entry of small solutes and macromolecules
Pericytes	Early pericyte injury, particularly in APOE4 carriers	Progressive pericyte degeneration	Significant pericyte loss and dysfunction	Increase BBB permeation; impair perfusion and perivascular clearance
BBB transporters/receptors	RAGE: early upregulation; TfR: preserved; GLUT1/P-gp/LRP-1: uncertain	RAGE↑; GLUT1: preserved to ↓; LRP-1-mediated Aβ efflux ↓; P-gp activity↓; TfR: preserved	RAGE ↑↑; GLUT1↓; LRP-1-mediated Aβ efflux ↓; P-gp activity ↓; TfR relatively preserved	Affect RMT and CMT efficiency; upregulated RAGE and preserved TfR may remain exploitable for RMT
Neuroinflammation	Emerging, heterogeneous endothelial/glial activation	Increasing endothelial/glial inflammation	Marked endothelial/glial inflammation; peripheral immune cell infiltration	Enable inflammatory endothelial (e.g. ICAM-1 and VCAM-1) targeting and cell-mediated delivery
Astrocytic endfeet	Subtle functional abnormalities; early disturbances of AQP4 organization emerge	Progressive endfoot dysfunction; increasing AQP4 mislocalization	Marked endfoot disorganization; perivascular AQP4 depolarization; glymphatic dysfunction	Affect the interstitial distribution, retention, and clearance of therapeutics after BBB penetration
Basement membrane	Early ECM/BM remodeling	Progressive BM/ECM remodeling and impaired perivascular drainage	Marked BM thickening/splitting; impaired perivascular Aβ clearance; CAA	Restrict NP perivascular transport and clearance; CAA increases hemorrhagic and edema-related risk

These stage-dependent alterations influence the BBB-crossing efficiency and safety of nanoparticles. At earlier stages, when endothelial junctions and cerebral perfusion remain relatively preserved, passive or paracellular entry is limited, favoring active transport mechanisms such as CMT and RMT for ligand-functionalized nanoparticles. As AD progresses, moderate but heterogeneous BBB leakage may provide additional paracellular access. However, progressive impairment and reduction of physiological BBB transporters and receptors, such as GLUT1 and LRP-1, may compromise the efficiency of CMT and RMT-mediated delivery [262]. TfR-mediated RMT is promising, as studies indicated that BBB TfR appeared comparatively preserved in human and AD mice [263]. Progressive endothelial RAGE upregulation provides a disease-associated targeting opportunity. Moreover, RAGE-targeting ligands can further facilitate subsequent lesion- and cell-directed drug delivery due to increased RAGE expression on diseased astrocytes, microglia and neurons in AD [264]. Importantly, increased BBB permeability does not necessarily translate into more effective or more precise brain delivery. Hypoperfusion, capillary stalling, and extracellular-matrix remodeling may limit nanoparticle arrival, brain distribution, and elimination [265]. AMT may increase nonspecific endothelial interactions and cationic toxicity in already compromised vasculature, potentially further aggravating BBB breakdown. BBB manipulation raises similar safety concerns, which may lead to edema, inflammation or microhemorrhage, particularly in patients with cerebral amyloid angiopathy [266]. Cell-mediated transport may exploit neuroinflammation-associated homing but may induce greater delivery variability and immunological risk [267]. Overall, optimal BBB-crossing strategies should fully consider these dynamic BBB alterations to achieve precision delivery with high efficiency and safety.

## 6 Second target: targeting AD pathology and specific cell types in the brain

Notably, crossing the BBB alone is insufficient to achieve precision drug delivery in AD treatment. Indeed, the non-specific distribution of therapeutics within the brain after BBB penetration not only diminishes the therapeutic effects for AD but also raises concerns regarding off-target drug activity and potential neurotoxicity. Therefore, when solving the BBB entry problems in AD nanoparticle design, emphasis should also be placed on targeting AD-related pathology or specific cell types for more precise drug delivery. Representative nanoplatforms directed toward AD pathology are summarized in Table 6, and cell-type-selective targeting approaches are summarized in Table 7.

**Table 6:** Representative NP strategies for AD pathology and microenvironment in the brain.

Targeting level	Targeting ligand/responsive moiety	Representative nanoparticles	AD model	Administration protocol	Therapeutic efficacy	Safety evaluation
Aβ pathology	KLVFF (RAGE) [[20], [89], [131], [159], [166], [174], [196]]	T-CeNP [145]	4-month-old 5×FAD mice	i.v.; 1 mg kg^−1^, twice weekly for 2 m	Improved cognition; reduced oxidative stress, Aβ, neuroinflammation, and microglial activation	Organ histopathology; blood biochemistry; body weight; acute toxicity (100× dose)
	QSH [[178], [268]]	SDP@Cur-CRT/QSH [178]	7-month-old APP/PS1 mice	i.v.; 25 mg kg^−1^ every 4 d for 3 m	Reduced Aβ and NLRP3-associated neuroinflammation; enhanced neuroprotection/neurogenesis; improved cognition	Cytotoxicity; hemolysis and thromboelastography; body weight; organ histopathology
	ApoE3 [269]	ApoE3-rHDL [269]	7-month-old SAMP8 mice	i.v.; 5 mg kg^−1^ DMPC daily for 4 w	Reduced Aβ deposition and microglial activation; preserved neuronal integrity; improved spatial learning and memory	Organ histopathology
	GM1 [270]	NAP-GM1-rHDL [270]	Hippocampal Aβ+ibotenic acid-induced AD mice	i.n.; lipid dose 5 mg kg^−1^ daily for 2 w	Reduced Aβ deposition and neuronal damage; enhanced neuroprotection; improved spatial learning and memory	Organ histopathology; nasal mucosa/olfactory nerve NSE immunohistochemistry
	BTA [188]	BTA-Fe_3_O_4_@MSN@C_3_N_4_ [188]	8-month-old APP/PS1 mice	i.v.; 5 mg kg^−1^ for 4 w	Reduced Aβ and neuronal apoptosis; Cu^2+^ chelation and Aβ fibril disassembly; improved cognition	Cytotoxicity; BBB/NIR safety; brain histology; organ histopathology
	Congo red [[192], [271]]	Congo red/Rutin-MNPs [192]	13-month-old APP/PS1 mice	i.v.; 2 mg daily for 40 d	Reduced Aβ plaques, ROS and neuronal loss; improved spatial learning and memory	Cytotoxicity; biodistribution/clearance by Prussian-blue staining
	1F12 antibody [[182], [187]]	RVG29-bMSNs@Ce-1F12 [182]	14-month-old APP/PS1 mice	i.v.; 10 mg kg^−1^ once weekly for 4 w	Reduced Aβ, ROS, p-tau and microglial activation; improved cognition	Cytotoxicity; biodegradation; hemolysis assays; organ histopathology
Tau pathology	DMK6240 [272]	Fe-MIL-88B-NH2-NOTA-DMK6240/MB [272]	5-month-old OA-induced AD rats	Intrahippocampal; single dose, (MB 1.5 mg mL^−1^)	Reduced p-tau aggregation and neuronal death; improved learning and memory	Cytotoxicity; organ histopathology
	4B1 (p-Tau^396,404^) [273]	HA-DMSN@4B1 [273]	10-month-old htau and 5×FAD mice	i.n.; 100 μg twice weekly for 4 w	Reduced p-tau^396,404^, neuronal loss, Aβ pathology and microglial activation; improved cognition	Cytotoxicity; hemolysis; body weight; organ and nasal-mucosa histology
	PROTAC-7 [274]	Glycoengineered PROTAC NPs [274]	9-month-old 3×Tg mice	i.v.; 12 doses over 28 d in 3×Tg mice	Lesion-restricted p-tau degradation; reduced tau phosphorylation/spread; restored synaptic plasticity and cognition	Organ histopathology, brain Nissl staining, hematology and serum biochemistry
	AT8 (p-Ser^202^/Thr^205^) [184]	THN [184]	Adult OA-induced AD rats	i.c.v.; single injection, (Fe: 600 μg mL^−1^)	Reduced pathogenic tau and microglial activation; enhanced autophagic tau clearance; improved cognition	Cytotoxicity
	T807 [117]	CeNC/IONC/MSN-T807-MB [117]	3-month-old OA-induced AD rats	Intrahippocampal; single dose, (MB 3.6 mg mL^−1^)	Reduced ROS, tau hyperphosphorylation, neuronal loss; improved spatial memory	Cytotoxicity; *in vitro*/*in vivo* degradation
	TLK [[167], [275]]	DGLs-PEG-RVG29-D-peptide/pshBACE1-AS NPs [167]	30-34-week-old APP/PS1 mice	i.v.; weekly from 30 to 34 weeks	Reduced p-tau-associated fibrils and Aβ plaques; reduced BACE1; improved spatial memory	Body weight; ALT/AST; toxicity and immunogenicity assessment
Inflammation lesions	Macrophage membrane [[130], [211], [212], [276], [277]]	MoS_2_ QDs/MM [211]	6-month-old APP/PS1 mice	i.v.; 5 mg kg^−1^ for 1 m	Improved learning and memory; reduced ROS, Aβ deposition, neuronal apoptosis, and IL-1β/IL-6/TNF-α	Cytotoxicity; body weight; organ histopathology
	CCR2 (CCL2) [132]	TR@CPL [132]	3-month-old 5×FAD mice	i.v.; 3 times/w for 6 w	Improved cognition; reduced Aβ plaques and glial activation/neuroinflammation; improved autophagy	Cytotoxicity; organ histopathology
ROS microenvironment	Boronic acids/esters [[89], [192], [214], [278]]	APBP dendrimer [89]	6-month-old APP/PS1 mice	i.v.; weekly for 3 m	Improved cognition; reduced ROS and Aβ plaques; normalized glial function and enhanced Aβ clearance.	Liver toxicity evaluation (ALT/AST); organ histopathology
	Thioketal [[90], [279]]	TT-NM/Rapa [90]	9-10-month-old 3×Tg-AD mice	i.v.; Rapa 0.24 mg kg^−1^ d^−1^ for 4 w	Improved cognition; restored autophagic flux; reduced Aβ, p-tau, microglial activation and neuronal loss	Cytotoxicity
	Diselenide bond [[99], [207]]	[99] exosome	10-month-old APP/PS1 mice	i.v.; 0.2 mL of 2 μg mL^−1^ NPs every 3 d, 7 doses	Improved cognition, TREM2 and microglial Aβ phagocytosis; Reduced BACE1, Aβ and neuroinflammation; promoted M2 polarization	Body weight; organ histopathology; hematology and blood biochemistry; liver/renal function

**Table 7:** Representative NP strategies for cell targeting in the AD brain.

Cell type	Targeting ligand	Representative nanoparticles	AD model	Administration protocol	Therapeutic efficacy	Safety evaluation
Astrocyte	DAG (CTGF) [[165], [224], [280]]	R@(ox-PLGA)-KcD [224]	8-month-old APP/PS1 mice	i.v.; 0.35 or 0.7 mg kg^−1^ every 2 d, 7 doses	Improved cognition; reduced Aβ/p-tau, oxidative stress and neuroinflammation; enhanced autophagic Aβ clearance	Body weight; organ histopathology
Astrocyte and microglia	Hyaluronic acid (CD44) [281]	PLGA-b-HA [281]	13-16-month-old APP/PS1 mice	i.v.; single 16 mg kg^−1^ injection	Preferential binding/retention in CD44^+^ reactive astrocytes and activated microglia; therapeutic efficacy not evaluated	Acute WBC profile; BUN/creatinine; ALT/ALP; albumin/globulin
	RVG29 (nAChR) [115]	RVG-NV-NPs/Bex [115]	9-month-old APP/PS1 mice	i.v.; Bex 1 mg kg^−1^ every 2 d for 1 m	Improved cognition; improved ApoE; reduced soluble Aβ and plaques; protected neurons from apoptosis	Cytotoxicity; hematology; ALT/AST, BUN, ALB, TP, TC, GLOB, and A/G
Microglia	COG1410 (LDLR) [282]	APND-3 [282]	6-month-old APP/PS1 mice	i.v.; 10 mg kg^−1^ every 2 d for 1 w	Improved microglial Aβ uptake/clearance; reduced Aβ deposition and neurological injury; improved cognition	Blood compatibility; organ histopathology
	MG1/CHHSSSAR [[171], [279]]	Res@TcMNP/ASO [171]	10-month-old APP/PS1 mice	i.v.; 2 mg kg^−1^ every 5 d, 7 injections	Reduced microglial activation and inflammatory cytokines; promoted M2 polarization; reduced Aβ/p-tau; improved cognition	Cytotoxicity; hemolysis; body weight; organ histopathology; ALT/AST/BUN/CREA;
	D-Mannose (mannose receptor) [[104], [283]]	Anti-17 MLNPs [104]	3.5-4-month-old 5×FAD mice	i.c.m.; 1.2 nmol anti-miR-17 weekly for 4 w	Restored autophagy; reduced neuroinflammation and Aβ burden; improved spatial memory	Cell-type off-target delivery assessed
	KLVFFAED (RAGE) [165]	DA-PP_TK_@PDA [165]	9-month-old APP/PS1 mice	i.v.; every 5 d for 3 m	Improved cognition; reduced ROS, Aβ, and neuroinflammation; restored glial function and neuronal regeneration	Hemolysis and cytotoxicity assays; organ histopathology
	α4β1 integrin/VCAM-1 on BV2 membrane [139]	hFGF21@BCM-LIP [139]	Aβ_1-42_-induced mice (20-30g)	s.c.; 0.5 mg kg^−1^ every 2 d for 2 w	Reduced microglial activation, IL-6/TNF-α, tau, p-tau and BACE1; improved cognition	Organ and lymph-node histopathology; glucose, AST, ALT
	VCAM-1 on MES23.5 membrane (α4β1 integrin) [197]	PBMn-IL3@CM [197]	6-week-old OA-induced AD-like mice	i.v.; 2 injections on d 6 and 9 after OA	Promoted M2 polarization; reduced ROS and p-tau; neuroprotection; improved cognition	Cytotoxicity; organ histopathology
Pericyte	VHS (VCAM-1) [284]	VLC@Cur-NPs [284]	14-month-old APP/PS1 mice	i.v.; 1.624 mg kg^−1^ once daily for 28 d	Restored pericyte density/coverage, BBB integrity, and CBF; Reduced ROS and inflammation; improved Aβ clearance, neuronal survival, and cognition	Cytotoxicity; hemolysis; body weight; hematology; hepatorenal function; organ histopathology
Neuron	Tet-1 (GT1b) [[90], [156], [164], [172]]	CT/BACE1-siRNA [164]	8-month-old APP/PS1 mice	i.v.; 80 μg siRNA/mouse every 5 d, 4 doses	Reduced BACE1, Aβ plaques and p-tau; promoted neurogenesis and restored cognition	Blood stability; hemolysis; myelination assessment
	RVG29 (nAChR) [[285], [286], [287], [288]]	RIF@PLA-PEG-Gd/Mal-RVG29 [288]	9-10-month-old APP/PS1 mice	i.v.; RIF-equivalent dose 20 mg kg^−1^ d^−1^; every 3 d × 8 doses	Improved cognition; Reduced Aβ and neuronal loss; restored synaptic ultrastructure and enhanced PSD95/SYP	Cytotoxicity; serum creatinine
	C3 (NCAM) [289]	CT-NM/Res [289]	9-month-old APP/PS1 mice	i.v.; Res 2 or 10 mg kg^−1^ d^−1^ for 4 w	Improved cognition; enhanced SIRT1; restored mitochondrial function; reduced Aβ, p-tau, and neuroinflammation	Cytotoxicity
	FGL (FGFR1 on cholinergic neurons) [158]	FGL-NP(Cit)/HNSS [158]	10-month-old 3×Tg-AD mice	i.v.; HNSS 0.3 mg kg^−1^ d^−1^ for 4 w	Improved cognition; restored mitochondrial function; reduced Aβ and p-tau; protected cholinergic neurons	Cytotoxicity; hematology; ALT/AST/BUN/CRE; serum stability; organ histopathology

### 6.1 Targeting AD pathology in the brain

#### 6.1.1 Aβ-targeted drug delivery

Aberrant deposition of neurotoxic Aβ represents a pathological hallmark of AD. In this context, Aβ-targeted drug delivery via nanotechnology holds significant potential for enhancing the therapeutic effects of AD while minimizing undesirable side effects. Versatile Aβ-specific targeting approaches have been reported, including the surface modification of Aβ peptides, Aβ monoclonal or polyclonal antibodies, or the incorporation of small molecules or compounds with high Aβ binding affinity. Among these, peptides, such as KLVFF, KLVFFAED, QSHYRHISPAQV, and LPFFD, are the most widely utilized for targeting Aβ specifically [[178], [290]]. KLVFF, a 5-amino-acid fragment derived from Aβ peptides (Aβ_16-20_), serves as an effective recognition motif and binding element for selectively targeting full-length Aβ through hydrogen-bonding interaction, π-π stacking, and hydrophobic interaction [[166], [291]]. As illustrated in Fig. 10A, Shan et al. fabricated a KLVFF-modified liposome that could specifically recognize and assemble with Aβ, thereby inhibiting its aggregation into toxic species, and promoting its clearance [174]. Together with nanozyme CeO_2_, this nanodrug system effectively reduced cerebral Aβ burden and attenuated oxidative stress in AD brains. Moreover, it was reported that KLVFF could actively target the AD microenvironment via RAGE, a cell-surface receptor highly expressed on BBB endothelial cells, neurons, and microglia that mediated the internalization of plasma Aβ to the CNS [20]. Another important strategy for Aβ targeting is the conjugation of Aβ-specific antibodies, which enable high-affinity binding to Aβ epitopes through precise molecular recognition [[1], [191]]. Zhang et al. developed an intelligent nanocomposite (bMSNs@Ce-1F12) modified with 1F12, an Aβ sequence- and conformation-specific antibody that could target all forms of Aβ species (Fig. 10B) [182]. As illustrated in Fig. 10C–F, with the help of 1F12, bMSNs@Ce-1F12 exhibited high binding affinity to soluble Aβ monomers, oligomers, protofibrils, and insoluble plaques, efficiently accumulating at the Aβ-rich brain regions of APP/PS1 mice after tail vein injection. Notably, Aβ antibodies can be further engineered to identify conformational epitopes exclusive to specific Aβ oligomers, allowing precise targeting of pathogenic forms and thereby avoiding interference with physiological Aβ and minimizing potential off-target effects [292]. In addition, several small molecules, such as benzothiazole aniline (BTA), Congo red, curcumin, and epigallocatechin gallate (EGCG), have also been used for Aβ targeting through structural complementarity and specific molecular interactions. For example, BTA derivatives, such as Pittsburgh Compound B and Thioflavin T, possess a planar rigid structure and an electron-rich aromatic system that can insert into the hydrophobic grooves of Aβ fibrils, stabilized by π-π stacking with aromatic residues and hydrogen bonding between the aniline -NH_2_ group and Aβ backbone carbonyls [[230], [293]]. Gong et al. designed a multifunctional Aβ nanocaptor that loaded carbon nitride nanodots to BTA-modified Fe_3_O_4_@MSN [188]. In this system, carbon nitride acted as a metal chelator that could effectively capture excessive Cu^2+^ to prevent Cu^2+^-Aβ complexes formation and decrease copper ions-associated toxicity. The Fe_3_O_4_ core enabled the nanocaptor to accumulate at the designated Aβ lesions under an externally applied magnetic field. BTA conjugation endowed the nanocaptor with specific binding capacity to the β-sheet structure of Aβ fibers as well as fluorescence imaging property for real-time detection of Aβ aggregates. Similarly, Congo red derivatives also exhibit >100-fold higher binding affinity for toxic Aβ aggregates over monomers due to recognition of the cross-β “steric zipper” architecture unique to amyloids [192]. Moreover, EGCG shows high selectivity towards pathogenic Aβ oligomers, resulting from not only molecular interaction and conformational remodeling, but also its remarkable ability of metal chelation, thereby dismantling neurotoxic metal (Cu^2+^/Zn^2+^)-Aβ complexes and suppressing ROS generation [294]. To summarize, all these strategies highlight the potential of precise Aβ-targeting delivery of AD drugs via nanotechnology.

**Fig. 10:**
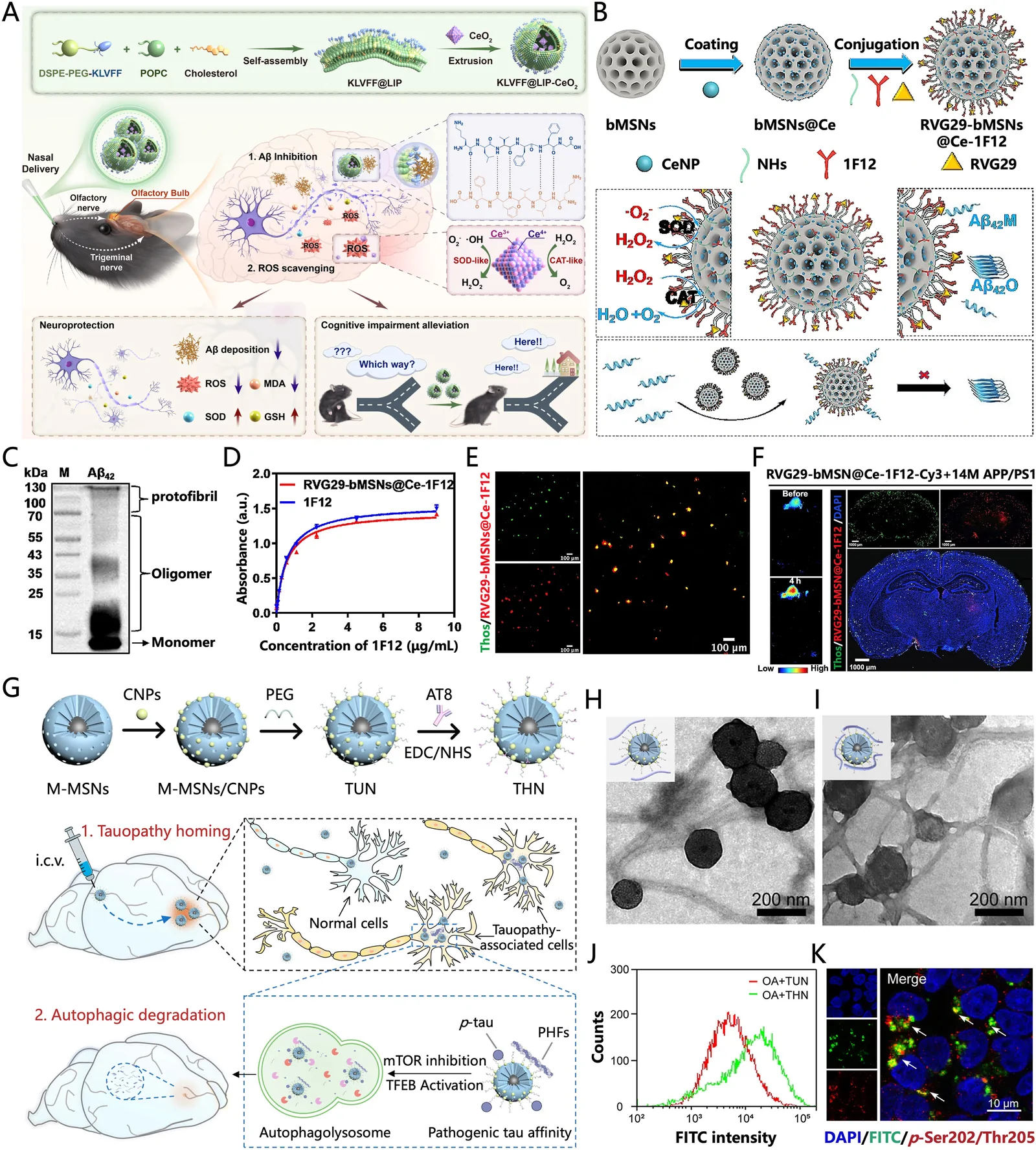
**AD pathology-targeted nanoparticles for precision drug delivery.** (A) Scheme of Aβ-targeted KLVFF@LIP-CeO_2_ for the treatment of AD. Reproduced with permission [174]. Copyright 2024, Elsevier. (B) Scheme of the synthetic procedure of RVG29-bMSNs@Ce-1F12 and its therapeutic mechanism. (C) Identification of various Aβ_42_ forms by immunoprecipitation-Western blot analysis of RVG29-bMSNs@Ce-1F12. (D) The binding affinity of RVG29-bMSNs@Ce-1F12 to Aβ_42_. (E) Representative images of Aβ plaques co-stained with RVG29-bMSNs@Ce-1F12 (red) and thioflavin S (green). Scale bar = 100 μm. (F) Representative *in vivo* whole-body fluorescent imaging and fluorescent images of brain Aβ plaques after tail vein injection of RVG29-bMSNs@Ce-1F12. Scale bar = 1000 μm. Reproduced with permission [182]. Copyright 2024, Springer Nature. (G) Scheme of tauopathy-homing nano-assembly (THN) with autophagy-activating capacity for AD treatment. (H-I) Transmission electron microscope images of PHF-treated tauopathy untargeted nanocomposite (TUN) and THN. (J) Flow cytometric analysis of cellular uptake of FITC-labeled THN and TUN by okadaic acid-treated SH-SY5Y cells. (K) Representative fluorescent images illustrating the co-localization of FITC-labeled THN (green) and phospho-Ser202/Thr205 of tau (p-Ser202/Thr205, red, representing pathogenic tau) in okadaic acid-treated SH-SY5Y cells. Reproduced with permission [184]. Copyright 2021, American Chemical Society. (For interpretation of the references to color in this figure legend, the reader is referred to the Web version of this article.)

#### 6.1.2 Tau-targeted drug delivery

Tauopathy is another major pathological hallmark of AD. Under AD conditions, hyperphosphorylated tau impairs microtubule stability and self-aggregates into insoluble PHFs and NFTs, leading to synaptic loss, neuronal dysfunction, brain atrophy, and ultimately cognitive deficit [28]. Given the crucial physiological functions of tau protein in neurons, precisely targeting pathogenic tau via nanotechnology is highly desired. Chen et al. reported a tau-targeted ligand T807 with preferential accumulation in tauopathy-affected regions [117]. They constructed a multifunctional nanosystem fabricated by loading methylene blue (a tau aggregation inhibitor) in the internal pores whilst modifying ceria nanocrystal (CeNC, a tau hyperphosphorylation inhibitor), iron oxide nanocrystal (IONC, a high-resolution MRI agent), and T807 on the surface of MSN. Facilitated by T807-mediated targeting, the nanocomposite selectively targeted hyperphosphorylated tau in neurons, concurrently inhibiting tau hyperphosphorylation, tau aggregation, and neuronal apoptosis. Similarly, Sun et al. designed a tauopathy-homing nano-assembly (THN) conjugated with AT8, a monoclonal antibody that selectively bound to phosphorylated epitopes (Ser202/Thr205) of pathogenic tau with high affinity and promoted the accumulation of nanoparticles in the tauopathy-associated neurons (Fig. 10G) [184]. As illustrated in Fig. 10H–K, THN exhibited preferential entanglement with PHFs and accumulation in okadaic acid-treated SH-SY5Y cells compared to the non-AT8-modified nanocomposites. Further *in vivo* results demonstrated that this nano-assembly was specifically retained in the hippocampus with tauopathy, where it facilitated the clearance of hyperphosphorylated tau by activating autophagy in diseased cells, ultimately contributing to alleviating neuronal degeneration and memory impairment in AD rats. In addition, a D-enantiomeric tau-targeting peptide (D)-TLKIVW was developed, employing the atomic structure of the tau aggregates motif (VQIVYK) as the template [295]. This peptide exhibited a high binding affinity to tau aggregates over tau monomers, achieved through hydrogen bonding and hydrophobic interaction with β-sheet-rich pathological assemblies. Sun et al. constructed an intelligent nano-inhibitor using self-assembled PCL-PEG polymeric micelles with the surface modification of this tau-binding peptide [275]. The fabricated nanocomposite specifically recognized and captured misfolded tau aggregates, inhibited their growth, blocked their transcellular propagation, and facilitated their proteolytic degradation, thereby remarkably alleviating tau-induced cytotoxicity and maintaining tau homeostasis in neurons. Overall, these fabricated ligands can be promising candidates for nanoparticle-mediated pathogenic tau-targeted AD therapy.

#### 6.1.3 Neuroinflammation-targeted drug delivery

Neuroinflammation serves as a critical pathogenic driver in AD progression, representing a significant target for precise AD drug delivery [296]. More recently, increasing interest has been paid to cell-membrane-camouflaged nanoparticles, as they not only exhibit extended blood circulation, reduced immunogenicity, and enhanced biocompatibility, but also possess a natural tendency to migrate to inflammatory regions, which derives from the inherent characteristics of their source cells, such as macrophages, neutrophils, microglia, platelets, and MSCs. Macrophage-derived membranes have been considered important materials for nanotechnology-mediated neuroinflammation targeting. Under AD pathology, substantial cytokines, chemokines, and growth factors are produced and released, promoting a significant overexpression of endothelial adhesion proteins (including P-selectin/E-selectin, intercellular adhesion molecule-1 (ICAM-1), and VCAM-1) and a massive infiltration of peripheral immune cells into AD lesions [297]. Macrophage membrane-coated nanoparticles, which highly express CD44 and Mac-1 on their surface, will be rapidly recruited, specifically recognize and bind to the upregulated ligands on inflamed endothelium, and thus reside in the sites of neuroinflammation [298]. For example, Qi et al. established a biomimetic multi-mode and multi-target nanosystem (MoS_2_QDs/MM) using the macrophage membrane to functionally modify MoS_2_ QDs. This membrane coating endowed the nanocomposite with active targeting performance and inflammation-oriented chemotaxis, thereby facilitating the accumulation of MoS_2_QDs/MM in APP/PS1 mice brains [211]. Similarly, neutrophil membrane-camouflaged nanoparticles can also mimic the adhesion process of neutrophils and precisely home to inflammatory pathological regions owing to the high affinity of the membrane proteins of neutrophils (adhesion factor receptors such as β-integrin, P-selectin glycoprotein ligand-1, and chemokine receptors such as CXCR2) to P-selectin/E-selectin at the neuroinflammatory focus [[299], [300]]. Most notably, recent research reported that neutrophil membrane-coated nanoparticles without drug loading also exhibited significant anti-inflammatory effects by neutralizing inflammatory cytokines, sequestering chemokines, as well as interfering with the process of adhesion, providing new insights for their applications in disease therapy [301]. Moreover, multiple cell membrane engineering strategies have recently emerged to introduce or increase the expression of relevant functional membrane proteins for improving their inflammation-specific targeting ability. For example, wild-type C1498 cells were genetically modified to stably express very late antigen-4 (VLA-4, a heterodimer composed of integrin α4 and integrin β1), which had specific affinity to the upregulated VCAM-1 on the inflamed endothelial cells, thereby promoting the migration of VLA-4-expressing membrane-coated PLGA nanoparticles to inflammatory sites [302]. In addition to cell membrane-camouflaged nanoparticles, multiple cell-derived EVs have similar inflammation-targeting effects due to functional surface proteins inherited from their source cells. Jiang et al. designed a hybrid nanovesicle by fusing Ang-2 peptide-modified liposomes and MSC-derived exosomes. Ang-2 peptide increased BBB crossing via LRP-1-mediated transport, while MSC-derived exosomes provided chemotaxis and homing ability towards the pathological sites of inflammation, thereby synergistically facilitating precision drug delivery to AD lesions in APP/PS1 mice [99].

#### 6.1.4 Other microenvironment-responsive drug delivery

Microenvironment-responsive drug delivery systems that respond to various stimuli at AD lesions enable spatially targeted delivery and on-site controlled drug release, providing an advanced platform for precision AD therapy [303]. In these systems, nanoparticles are engineered to react to specific biological, chemical, and physical signals in the AD pathological microenvironment, including endogenous stimuli (ROS, pH, and enzyme activity) and external stimuli (ultrasound, magnetic force, temperature, and laser) [[304], [305]].

Harnessing endogenous stimuli offers great potential to achieve brain regional accumulation of nanoparticles within the AD microenvironment. Oxidative stress appears as an important determinant in AD pathogenesis and progression, making ROS-responsive nanoparticles an ideal platform for targeted drug delivery to AD lesions. Nanomaterials can be incorporated with ROS-sensitive moieties and linkers, such as thioketal, selenide, boronic acids/esters, and thioether [306]. Yang et al. designed a ROS-responsive nanocomposite introducing the thioketal bond to the PLGA nanoparticles, which was stable in acidic or basic solutions but underwent cleavage into two thiol fragments and acetone upon exposure to ROS. By virtue of this property, the nanocomposite exhibited fast and enhanced drug release when arriving at the AD microenvironment characterized by elevated levels of ROS [146]. Boronic acids/esters are sensitive to oxidative stimuli, as the C-B bond will be cleaved when exposed to ROS, leading to a cascade of self-immolative electronic elimination and ROS scavenging, thereby making them highly suitable materials for the development of ROS-responsive nanoparticles [306]. Liu et al. fabricated a PEG-based phenylboronic dendrimer for delivering p-Nrf2 to the AD microenvironment. The incorporated phenylboronic ester groups not only eliminated ROS but also enabled precise p-Nrf2 release, synergistically restoring cellular endogenous antioxidant ability, mitigating glial activation, and eventually improving the memory deficits of AD mice [89]. Jiang et al. added DSPE-Se-Se-PEG-Ang2 in the formulation of liposomes, which would rupture due to the Se-Se bond breaking in ROS, thus ensuring the stability of nanovesicles during systemic transport and site-specific drug release after reaching AD lesions [99]. In addition to oxidative stress, postmortem histopathological analyses revealed a significantly lower mean intracellular pH in the brains of AD patients, providing opportunities for pH-sensitive nanoparticle-mediated therapy to achieve on-site controlled drug release in the AD microenvironment [307].

Similarly, external manipulations by magnetic force, ultrasound, temperature, and light fields are increasingly exploited to dictate nanoparticle localization within AD pathological areas. For example, drug delivery systems based on magnetic nanoparticles or loading materials with magnetic properties can be directed to the AD lesions under precise magnetic field control. Hour et al. labeled the MSCs with superparamagnetic iron oxide nanoparticles, successfully achieving targeted delivery to the hippocampus of AD rats through magnetic guidance [308]. Focused ultrasound is another noninvasive technique that can not only temporarily open the BBB for drug passage but also control drug release. Nisbet et al. managed to deliver RN2N (an anti-tau scFv) with the assistance of focused ultrasound in a scanning mode, remarkably enhancing the uptake of RN2N by neurons where tau was predominantly localized and leading to improved therapeutic outcomes in AD mice [309]. Thermo-responsive nanoparticles will undergo reversible physicochemical transformations and exhibit on-site controlled drug release at specific temperature thresholds (primarily regulated by lower and upper critical solution temperature), having been studied for AD therapy. Kim et al. designed linear-dendritic nanoparticles based on thermo-responsive materials, including poly (L-lysine), poly (L-lactic acid), and poly (N-isopropylacrylamide), facilitating thermal-targeting and sustained release of nerve growth factor for AD therapy [310]. Notably, despite demonstrated efficacy and biosafety in many studies, external stimuli-responsive delivery systems for AD therapy are still in the preclinical development phase, and their potential safety profiles in humans remain undefined. Furthermore, these techniques require more precise modulation of the intensity, frequency, and duration of external stimuli to optimize therapeutic outcomes while minimizing potential damage to surrounding brain tissue.

### 6.2 Targeting specific cells in the AD brain

#### 6.2.1 Microglia-targeted drug delivery

Microglia are innate immune cells of the myeloid lineage that reside in the brain, playing an essential role in maintaining overall homeostasis of the CNS by eliminating dead cells and debris, regulating synaptic pruning, modulating neuronal plasticity and neural circuits, and providing immune surveillance [42]. During the early phase of AD, microglia can recognize pathogenic Aβ via damage-associated molecular patterns receptors, and then phagocytize and degrade them through multiple endocytic pathways, thereby exerting neuroprotective effects on the CNS. Along with disease progression, chronic immune stimulus leads to functional impairments of microglia, which are prone to continuous activation and pro-inflammatory states, contributing to exacerbating neuroinflammation and accelerating AD progression [40]. Therefore, it is promising to exploit nanoparticles for precision drug delivery to pathological microglia, thereby modulating microglial activation towards a phagocytic and anti-inflammatory phenotype for the treatment of AD. Notably, the phenotypic variance between healthy and diseased microglia presents unique opportunities for targeted delivery. For example, Terashima et al. identified the MG1 peptide (C-HHSSSAR-C) through *in vitro* and *in vivo* biopanning with phage display libraries, which exhibited high affinity for M1 microglia that represent the pro-inflammatory phenotype [311]. In a recent research, Wei et al. fabricated a cascade-targeted nano-modulator (Res@TcMNP/ASO) based on the cationic liposome that was decorated with acid-cleavable BBB-targeting peptide D-T7 and M1 microglia-specific peptide MG1, achieving the co-delivery of TREM2-lowering ASOs and resveratrol (Fig. 11A). After crossing the BBB mediated by D-T7 peptide-transferrin receptor interaction, Res@TcMNP/ASO selectively targeted activated M1 microglia, restoring microglial phagocytosis ability by TREM2-lowering ASOs and mitigating the immune stimulation of ROS by resveratrol, thus collectively suppressing the sustained activation of microglia and alleviating chronic neuroinflammation in APP/PS1 mice (Fig. 11B) [171]. Liu et al. developed a multifunctional superparamagnetic iron oxide nano-system with surface modification of Aβ oligomer-specific antibody W20 and XD4 peptide that could target class A scavenger receptors on microglia. This dual-targeting design promoted efficient phagocytosis and clearance of Aβ, significantly attenuating neuropathology and rescuing cognitive impairment of APP/PS1 mice [191]. Another potential alternative to achieve high-precision targeted drug delivery to microglia is to exploit the microglia-derived membranes or EVs, which possess a natural tendency to home to their source cells. Accumulating evidence indicates elevated expression of α4/β1 integrin, VCAM-1, and chemoattractants on the surface of microglia [312]. Therefore, Wang et al. developed a hybrid nanocomposite based on PEGylated-liposomes coated with BV2 microglial membrane (hFGF21@BCM-LIP) for the precise delivery of hFGF21 to activated microglia in AD brains. Owing to the α4/β1 integrin and VCAM-1 interaction between hFGF21@BCM-LIP and pathogenic microglia, hFGF21 was selectively accumulated in the targeted microglia with high efficiency, thereby inhibiting microglia-mediated neuroinflammation and alleviating cognitive impairment of AD mice [139]. In addition to the above strategies, other ligands, such as mannose, COG1410, and the CD11b antibody, have also been used to actively target receptors on microglia surfaces and enhance intracellular drug uptake, thus providing more opportunities for precision microglia-specific drug delivery [[104], [282], [313]].

**Fig. 11:**
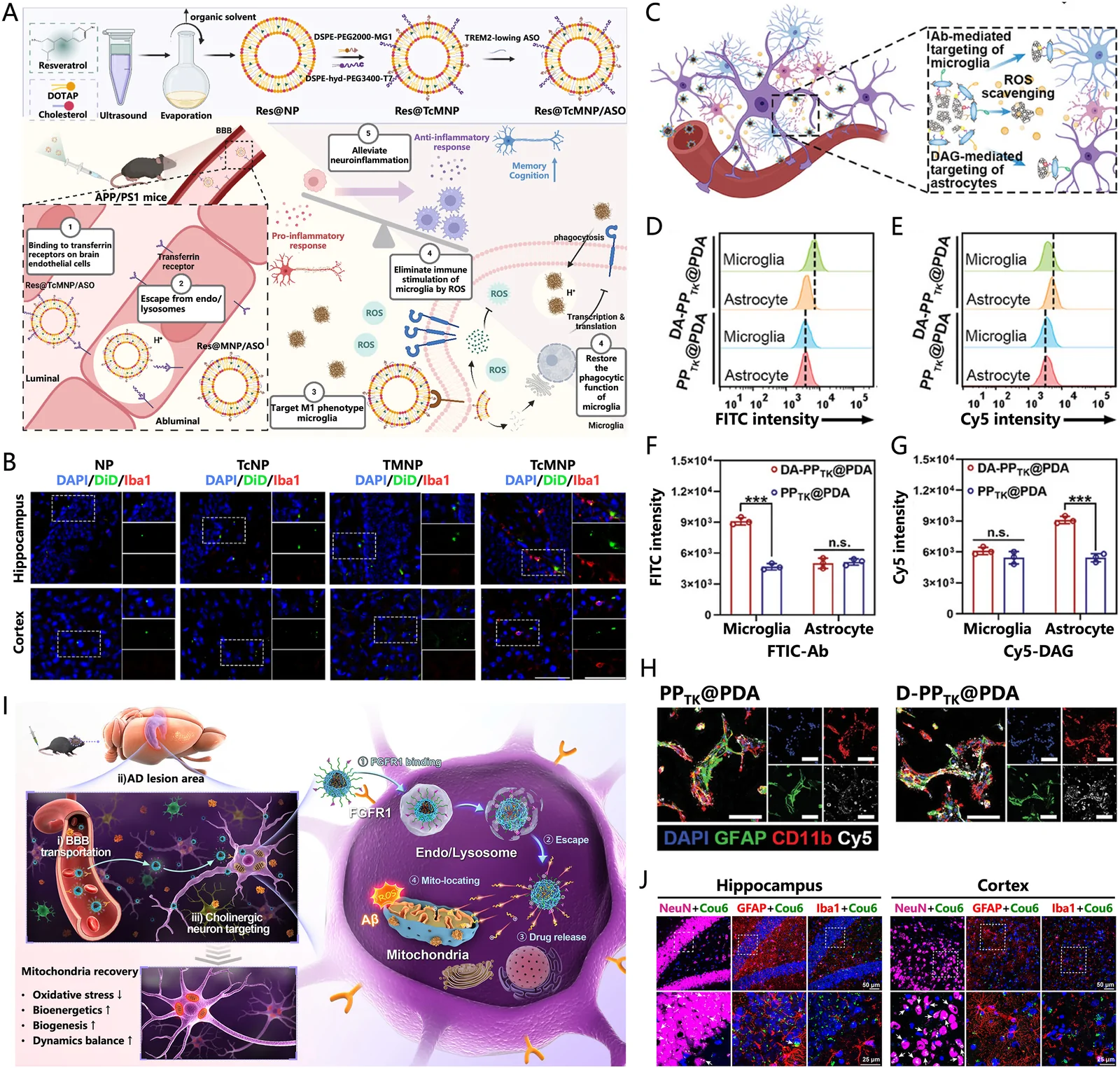
**Specific cell type-targeted nanoparticles for precision drug delivery.** (A) Schematic illustration of nanomodulator (Res@TcMNP/ASO) synthesis, BBB penetration, M1 microglia targeting, and alleviation of microglia-associated neuroinflammation in APP/PS1 mice. (B) Representative fluorescent images illustrating the co-localization of nanoparticles (Green) and microglia (Red) in the hippocampus and cortex. Scale bar = 50 μm. Reproduced with permission [171]. Copyright 2025, Elsevier. (C) Schematic illustration of the disassembly of nanoparticles in the pro‐inflammatory microenvironment and glial cell-specific targeting mechanism. (D-E) Flow cytometry analysis of cellular uptake of FITC-labeled and Cy5-labeled PP_TK_@PDA or DA-PP_TK_@PDA in primary microglia and astrocytes. (F-G) Quantification of fluorescent intensity of FITC-labeled A-PPH (F) and Cy5-labeled D-PPA (G) in primary astrocytes. (H) Representative fluorescent images of the internalization of Cy5-labeled PP_TK_@PDA and D‐PP_TK_@PDA in co‐cultured primary microglia and astrocytes at 1 h post-incubation. Scale bar = 100 μm. Reproduced with permission [165]. Copyright 2024, John Wiley and Sons. (I) Schematic illustration of FGL-NP(Cit)/HNSS crossing the BBB and specifically targeting cholinergic neurons in AD lesions with high FGFR1 expression. (J) Representative fluorescent images illustrating the distribution of nanoparticles in the hippocampus and entorhinal cortex in 3×Tg-AD mice at 1 h post-injection. Reproduced with permission [158]. Copyright 2022, American Chemical Society. (For interpretation of the references to color in this figure legend, the reader is referred to the Web version of this article.)

#### 6.2.2 Astrocyte-targeted drug delivery

Astrocytes are acknowledged to form the scaffold of the entire CNS, serving vital housekeeping functions including regulating cerebral blood flow, participating in BBB composition, maintaining neurotransmitter homeostasis, promoting synaptogenesis, and supplying neurotrophic support [40]. Under AD conditions, astrocytes respond to pathological insults, such as Aβ aggregates and hyperphosphorylated tau, and alter towards A1 phenotypes that are toxic to the CNS, contributing to the exacerbation of neuroinflammation, dysregulation of microcirculation, and disruption of the BBB, and eventually leading to synapse loss and impaired cognition [43]. Therefore, targeted delivery to astrocytes makes sense for alleviating neuropathology for AD therapy. Several ligands, such as DAG peptide, triphenylphosphonium cation, reactive astrocyte-homing peptide (AS1), lipopolysaccharide, and gH625 peptide, are reported to be promising for preferential delivery of drugs to astrocytes [[75], [165]]. Connective tissue growth factor, a matricellular protein, is significantly upregulated on astrocytes in AD brains and constantly overexpressed along with astrocyte reaction, serving as an important mediator for astrocyte-targeting transport of substrates (such as DAG peptide) [165]. Recently, Liu et al. proposed a modular-designed nanoplatform (DA-PP_TK_@PDA) modified with Ab peptide (an Aβ-derived peptide) for microglia-specific delivery of hydroxychloroquine, and DAG peptide for astrocyte-specific delivery of all-trans retinoic acid, aiming to restore the immune function of chronic-tolerant microglia and trans-differentiate activated astrocytes into functional neurons in AD brains (Fig. 11C). As illustrated in Fig. 11D–H, the DAG-decorated nanoparticles (D-PP_TK_@PDA) promoted the uptake of Cy5-conjugates by astrocytes but not by microglia compared to non-decorated nanoparticles, confirming that DAG modification can effectively facilitate selective drug delivery to activated astrocytes. CCL2, a cytokine primarily secreted by astrocytes, is suggested to be up-regulated in many neuropathic conditions, effectively recruiting cells that express CCR2 to the affected region. Based on this, Lin et al. formulated a biomimetic nanovesicle by hybridizing platelet membranes and HEK293T cell membranes that were engineered to overexpress CCR2. After intravenous injection, the drug-loaded nanoparticles actively migrated to the neuroinflammation-related lesions due to the recruitment of increased CCL2, significantly enhancing drug accumulation in both microglia and astrocytes within 5×FAD transgenic AD mice brains.

#### 6.2.3 Neuron-targeted drug delivery

Neurons, the fundamental units of the CNS, undergo irreversible damage and loss of structure and function in AD, directly contributing to cognitive decline and disease progression. Therefore, precision drug delivery to injured neurons for protection and restoration is of fundamental importance for effective AD therapy. Research has illustrated that the physical properties of nanoparticles, including size, morphology, and surface charge, significantly influence their cellular internalization by neurons. Reportedly, nanoparticles with a smaller size or negative charges are preferentially internalized by neurons and have an advantage for nanotechnology-mediated neuron-targeting [75]. In addition, various cell-penetrating peptides are increasingly being utilized for the functionalization of nanoparticles, as they can actively target the neuron-specific receptors and facilitate the precision drug delivery to neurons. Tet-1 peptide, a 12-amino acid fragment derived from the carboxy-terminal region of the tetanus neurotoxin, exhibits high binding affinity to GT1b ganglioside receptors that are abundantly expressed on neurons [314]. Based on this, Guo et al. developed a brain neuron-targeted nano-combination system dually modified with the BBB-targeted peptide CGN and the neuron-targeted peptide Tet1. The nano-system exhibited more drug accumulation in diseased neurons by cascade targeting via CGN and Tet1, and improved synergistic therapeutic efficacy of the loaded H102 and NAP in 3×Tg-AD transgenic mice [156]. RVG 29, a 29-amino acid peptide originating from the coat protein of rabies virus, serves as a targeting moiety binding to the nAChRs that are highly expressed on the neuronal surface [315]. This targeting specificity enables the RVG29 peptide to facilitate the efficient uptake of conjugated nanoparticles by neurons, which has been widely used in neuron-targeted drug delivery. For instance, Zhou et al. reported that compared to non-modified nanoparticles, RVG-modified nanoparticles exhibited a higher BBB penetration efficacy and more drug accumulation in the neurons of APP/PS1 mice, contributing to improved therapeutic effects in neuron protection and rescue of cognitive impairment [288]. Moreover, in a recent study, Huang et al. engineered primary NSCs by Lamp2b-RVG-containing adenovirus to acquire RVG-overexpressed NSC membrane, which was further used for the coating of PLGA nanoparticles loaded with bexarotene and AgAuSe QDs. NSCs are known to have a natural brain-homing property, tending to move towards homologous cells and home to injury sites in the brain. Therefore, by the synergistic effects of NSC membrane and overexpressed RVG peptide, the nanocomposite achieved significant enhancement of BBB penetration and precise drug delivery to neurons in AD brains [115]. In another study, RVG29 was co-functionalized on the surface of the RBC membrane together with triphenylphosphine cation, a mitochondria-targeted ligand which could induce mitochondrial internalization driven by the negative mitochondrial membrane potential [180]. By combination with the RVG29 and triphenylphosphine cation, the resveratrol-loaded nanoparticles not only targeted the neurons in the brain but also further precisely localized to the mitochondria, significantly alleviating Aβ-induced mitochondrial oxidative stress and cognitive deficits of APP/PS1 mice. These neuronal-mitochondria cascade-targeted nanoparticles provide a novel strategy for precision mitochondrion-targeted therapy delivery for AD. However, studies revealed that other cells, including microglia and astrocytes, can also exhibit the overexpression of nAChRs under pathological conditions, undermining the specificity of RVG29-mediated neuron-targeting. Fibroblast growth factor receptor 1 (FGFR1) is reported to be abundantly and specifically expressed on neurons, particularly on cholinergic neurons in areas that show the greatest vulnerability to AD pathology [316]. Thus, Qian et al. developed a multifunctional nano-system modified with FGL peptide, a neuronal cell adhesion molecule mimetic peptide with strong binding capacity to FGFR1 (Fig. 11I). As illustrated in Fig. 11J, compared to microglia and astrocytes that predominantly express FGFR2 and FGFR3, FGL-modified nanoparticles were preferentially internalized by neurons in the entorhinal cortex and hippocampus, exhibiting 2.2-fold greater neuron-targeted specificity than non-modified nanoparticles [158]. In addition, some receptors and transporters, such as RAGE, are found to be differentially expressed on neurons between healthy humans and AD patients, offering unique opportunities to develop diseased neuron-specific delivery systems that maximize targeting precision while minimizing off-target effects, which provide new insights and hold great promise for future advancements [317].

## 7 Future perspectives

In this review, we comprehensively analyzed the recent advances in precision nanoparticle-based drug delivery systems for AD therapy. The past few decades have seen a burgeoning interest in various types of nanoparticles, as well as diverse nano-techniques enabling more precise drug delivery to AD lesions. Despite the significant progress made thus far, most of them remain in the preclinical stages. Therefore, enormous work is imperative to realize the transition from the current investigation to eventual clinical application and popularization. Some potential challenges in clinical translation that must be overcome in future research are suggested as follows:

**Clarifying the exact pathogenesis of AD and developing multi-target combination therapies:** Current clinical drugs for AD treatment only serve to alleviate the symptoms but fail to cure AD or halt the disease progression, which primarily results from the incomplete understanding of its underlying mechanisms. Therefore, clarifying the exact pathogenesis to identify precise therapeutic targets and investigate novel drugs and medical interventions can be the cornerstone of DMT for AD. As discussed in the previous context, AD is characterized by a complex and interconnected network of different pathological processes rather than isolated molecular events. Dynamic pathological crosstalk between multiple mechanisms, such as Aβ accumulation, tau pathology, and neuroinflammation, collectively drives neurodegeneration and accelerates disease progression, seriously challenging the conventional drug discovery approaches rooted in the “one molecule, one target” paradigm. Therefore, single-target medications have consistently demonstrated high rates of failure in clinical trials, highlighting the necessity to elucidate the underlying dynamic crosstalk among multiple pathogenic factors and develop multi-target combination therapies. Based on this, versatile nanoparticles hold great promise to fully take advantage of their high drug-loading capacities, serving as a robust platform that concurrently targets these interconnected pathological pathways and achieves more effective AD treatment.

**Optimizing nanoparticle physicochemical properties by machine learning and artificial intelligence:** Different physicochemical and material parameters, including the size, morphology, composition, surface charges, and surface modifications, collectively contribute to the biocompatibility, drug loading capacity, *in vivo* and storage stability, pharmacokinetics, BBB permeability, and targeting efficiency of nanoparticles. Thus, future studies should consistently optimize these properties to enhance their *in vivo* safety, therapeutic efficacy, and clinical translation potential. Fortunately, recent advances in machine learning and artificial intelligence can provide a set of feasible and robust tools to facilitate the rational design and refinement of nanoparticles. Multiple artificial intelligence models based on machine learning algorithms, such as support vector machines, random forests, and extreme learning machines, have been established to predict the *in vivo* biocompatibility, biodistribution, pharmacokinetics, and pharmacodynamics of nanoparticles through the analysis of high-dimensional datasets [318]. These techniques can also provide detailed insights into the complicated interactions among nanocarriers, drugs, and specific cells at molecular scales [319]. Furthermore, due to their high throughput screening abilities, machine learning and artificial intelligence can accelerate the discovery, identification, and selection of novel cell-penetrating peptides, homing peptides, or targeting ligands specific to the BBB, AD pathology, and affected cells, enabling the development of nanoparticle-based delivery systems with higher BBB permeability and more efficient precision targeting [320]. Overall, machine learning and artificial intelligence-aided optimization of design and synthesis hold great promise to drive forward clinical translation of precision nanoparticle-based AD therapy.

**Improving nanoparticle *in vivo* behavior and long-term biosafety:** Although numerous preclinical studies have reported satisfactory biocompatibility of nanomaterials, their clinical safety requires more rigorous validation, particularly considering the significant cross-species differences and complex nanoparticle-host interactions. Extensive efforts are needed to optimize relevant parameters to minimize the intrinsic toxicity of nanomaterials, as well as verify their biosafety across multiple biological models. Beyond intrinsic toxicity of nanomaterials and their degradation products, the *in vivo* behavior of nanoparticles following systemic administration also critically influences their safety profile. In the bloodstream, plasma proteins rapidly adsorb onto nanoparticle surfaces, forming a protein corona that can alter particle size, surface properties, cellular recognition, pharmacokinetics, and biodistribution [321]. Importantly, corona formation may partially mask or reorganize targeting ligands, reducing their specificity and disrupting interactions with BBB transporters or receptors, thereby impairing BBB penetration and lesion-selective delivery [322]. Meanwhile, opsonization and sequestration by the MPS can cause substantial hepatic and splenic accumulation, reducing the nanoparticle fraction available for BBB crossing while increasing off-target organ exposure [74]. Strategies that improve lesion-specific delivery may help mitigate some of these risks. Computational modeling, including molecular docking and molecular dynamics simulations, can facilitate the rational screening and optimization of targeting ligands and multi-ligand surface-engineering strategies for cascaded precision targeting [323]. However, increasingly complex surface modification may introduce receptor saturation, altered pharmacokinetics, and immunogenicity concerns [324]. Similarly, externally assisted strategies, such as focused ultrasound, despite offering enhanced spatiotemporal control of nanoparticles, may increase additional risks of BBB or vascular injury. These issues require careful evaluation to balance targeting efficiency against safety in translational applications. Furthermore, long-term toxicity assessment is particularly important for AD, where repeated treatment is likely to be necessary. Although single-dose toxicity may be limited, repeated exposure can induce complement activation, anti-carrier or anti-ligand immune responses, altered pharmacokinetics, or cumulative retention of inorganic or poorly biodegradable nanoparticles. Future studies should therefore systematically characterize repeated-dose pharmacokinetics, immunogenicity, degradation, clearance, and long-term accumulation in both the brain and peripheral organs. Machine learning and artificial intelligence offer new prospects for predictive assessment of intrinsic toxicity, *in vivo* behavior, and repeat-dose feasibility, thereby supporting the rational design of safer nanocarriers for long-term administration [325].

**Constructing more realistic BBB and AD models:** The development of more realistic models for evaluating BBB permeability is imperative. To date, two-dimensional monolayer cell models, such as *in vitro* transwell-based systems, are commonly employed as a standard technique to assess the BBB-crossing ability and quantify penetration efficiency of nanoparticles. However, these traditional static BBB models do not fully recapitulate the structural and functional complexity of the human NVU and BBB, as they do not account for the critical influence of shear stress induced by the blood flow. On the other hand, another major challenge is the absence of preclinical AD models that truly represent the human condition. AD is a complicated and multifactorial disorder; yet, most established animal models for AD, such as the widely used APP/PS1 transgenic rodents or those injected with Aβ or tau proteins, primarily focus on a single pathogenic contributor to the AD phenotype, thus failing to replicate brain physiology or reflect the human-like AD pathologies accurately. In addition, cross-species variations, including disparities in neuron and glia structure, receptor expression, and ligand affinity between humans and animals, further limit their ability to predict human therapeutic responses. Thus, promising therapeutic outcomes and safety profiles observed in preclinical studies are often unable to be reproduced in human patients, highlighting the urgent need for more realistic and truly representative preclinical AD models to bridge the gap. Notably, emerging advanced technologies, such as microfluidics, organoids, and organ-on-a-chip, have recently attracted widespread attention for their great potential to address these challenges [326]. BBB organoids, which are generated by co-culturing BMECs, pericytes, and astrocytes, can reproduce key barrier features, such as tight-junction formation, molecular transporters, and drug-efflux pumps [327]. Therefore, these 3D BBB systems offer considerable potential for screening nanoparticle permeability, evaluating BBB-targeting efficiency, and investigating transport mechanisms [328]. Moreover, human brain organoids, particularly those derived from patient-specific induced pluripotent stem cells, provide an important platform by preserving human genetic backgrounds and recreating 3D neural tissue organization [329]. Patient-derived brain organoids with familial AD-associated mutations have been reported to spontaneously reproduce key AD-related phenotypes, including Aβ accumulation, tau hyperphosphorylation, endosomal abnormalities, and neuronal dysfunction, and have been applied to pharmacological testing and drug screening [330]. More advanced vascularized and neuroimmune organoids further incorporate vascular cells, microglia, astrocytes, and neurons, enabling simultaneous investigation of NVU dysfunction, neuroinflammation, and neuronal pathology. Notably, a recently developed vascularized neuroimmune organoid model exposed to brain extracts from sporadic AD patients could reproduce Aβ and tau aggregates, microglial activation, excessive synaptic pruning, neuronal loss, and impaired neural network activity [331]. This model was further employed to evaluate responses to lecanemab, highlighting the potential of organoids for human-relevant therapeutic assessment. Brain-on-a-chip devices are highly biomimetic cell-based systems that utilize microfluidic, 3D tissue engineering, and semiconductor-based technologies to artificially recapitulate the complex structure and functionality of the human BBB and brain parenchyma *in vitro*, providing a more pathologically realistic and clinically relevant preclinical model [332]. These devices typically integrate human-derived neural cells, such as neurons and glial cells, within a precisely controlled microenvironment that includes extracellular matrix scaffolds and fluidic channels to simulate perfusion and nutrient exchange [333]. Thus, brain-on-a-chip not only replicates the dynamic fluidic microenvironment and transport processes of the BBB, but also facilitates real-time monitoring of cellular responses, including electrical activity, cell-cell interactions, drug-cell interactions, cell-nanoparticle interactions and cell-matrix interactions in AD pathophysiology [334]. Moreover, compared to animal models, brain-on-a-chip exhibits lower cost and higher reproducibility, representing a promising alternative for investigating drug delivery strategies and therapeutic effects in AD treatment. Future integration of patient-derived brain organoids and organ-on-chip technologies may combine the biological complexity of organoids with the dynamic control of microphysiological systems, providing a more comprehensive platform for evaluating BBB transport, intracerebral distribution, cell-specific targeting, therapeutic efficacy, and safety of precision nanomedicines for AD.

**Developing intelligent and multifunctional nano-systems:** Future investigations should seek to fabricate more intelligent multifunctional nanoparticle-based systems that enable the integration of navigation, real-time imaging, diagnostics, monitoring, and therapeutic capabilities for AD. For example, nanoparticle-mediated co-delivery of AD-specific small-molecule diagnostic agents, such as probes or biosensors targeting Aβ, tau, ROS, and metal ions, provides a non-invasive approach for real-time detection and tracking of AD biomarkers, contributing to the improvement of early screening, diagnosis, surveillance, and clinical intervention for AD patients. In addition, the incorporation of multimodal neuroimaging technologies, such as near-infrared II fluorescence imaging, ultrahigh-field MRI, and positron emission tomography, allows real-time *in vivo* monitoring of nanoparticle movement and localization, offering valuable insights into their biodistribution and targeting efficiency. More importantly, certain imaging agents, such as near-infrared probes, offer promising opportunities for the synergistic integration of photodynamic and photothermal therapies for AD, thus facilitating a versatile nano-system that combines imaging, diagnosis, and treatment functions within a unified platform. Overall, such multifunctional nano-systems hold great promise for the comprehensive management of AD.

**Establishing a translational framework for manufacturing, quality control, and regulatory compliance**: Beyond therapeutic efficacy and safety concerns, the clinical translation of nanomedicine critically depends on robust chemistry, manufacturing, and controls. Scalable and reproducible manufacturing remains a major barrier, as nanoparticle properties are highly sensitive to formulation composition and processing conditions. Key parameters, including particle size and polydispersity, morphology, surface charge, ligand density, and release kinetics, may change during the transition from laboratory-scale preparation to industrial production, ultimately affecting *in vivo* behavior, therapeutic efficacy, and safety. Therefore, critical quality attributes and critical process parameters should be defined to ensure batch-to-batch consistency and compliance with good manufacturing practice requirements [335]. These challenges are amplified when producing multi-component or biomimetic systems. The incorporation of multiple targeting ligands, stimuli-responsive moieties, imaging agents, or therapeutic cargos expands the number of material attributes and manufacturing variables that must be characterized and controlled. This increased complexity further complicates analytical characterization, scale-up, stability assessment, and regulatory compliance. Biomimetic systems are particularly challenging to manufacture and standardize due to heterogeneity in biological sources, cell culture conditions, membrane isolation, vesicle purification, residual impurities, membrane integrity, and surface-protein composition or activity [336]. In addition, sterilization requires formulation-specific optimization, as conventional procedures such as heat treatment, irradiation, and filtration may alter nanoparticle structure, surface functionality, lipid integrity, or cargo activity [337]. Moreover, long-term storage stability represents another essential translational requirement, as aggregation, fusion, hydrolysis, oxidation, and premature cargo leakage may compromise physicochemical stability and biological potency. Accordingly, a robust translational framework integrating scalable manufacturing, quality control, sterilization, storage stability, and regulatory compliance is essential for the clinical translation of nanomedicine.

To conclude, precision nanoparticle-based drug delivery systems represent a rapidly advancing and highly promising field in the AD diagnosis and therapy. While significant challenges lie ahead, ongoing multidisciplinary collaboration among basic researchers and clinicians in materials science, biotechnology, neurobiology, and medicine is steadily narrowing the divide between research discoveries and clinical practice, thus accelerating the translation of nanoparticle-mediated therapies from bench to bedside. With continuous investigation and refinement, precision nanomedicine is poised to revolutionize AD management strategies in the future.

## CRediT authorship contribution statement

**Qiujie Shan:** Writing – original draft, Investigation, Data curation, Conceptualization. **Wenao Zheng:** Writing – review & editing, Validation. **Weina Yao:** Writing – review & editing, Conceptualization. **Yamei Bai:** Writing – review & editing. **Wanchuan Ding:** Writing – review & editing, Validation. **Ji Wang:** Writing – review & editing, Supervision, Conceptualization. **Feng Bai:** Writing – review & editing, Supervision, Funding acquisition, Conceptualization.

## Compliance with ethics requirements

This article does not contain any studies with human or animal subjects.

**Table:** List of Abbreviations

Aβ	β-amyloid
ACh	acetylcholine
AChEI	acetylcholinesterase inhibitor
AChR	acetylcholine receptor
AD	Alzheimer's disease
ADSC	adipose-derived mesenchymal stem cell
ALB	albumin
ALP	alkaline phosphatase
ALT	alanine aminotransferase
AMT	adsorptive-mediated transcytosis
Ang-2	angiopep-2
ApoE	apolipoprotein E
APP	amyloid precursor protein
APP/PS1	APPswe/PSEN1dE9
AQP4	aquaporin-4
ASO	antisense oligonucleotide
AST	aspartate aminotransferase
ATP	adenosine triphosphate
AuNP	gold nanoparticle
BACE1	β-site amyloid precursor protein-cleaving enzyme 1
BBB	blood-brain barrier
BDNF	brain-derived neurotrophic factor
BM	basement membrane
BMEC	brain microvascular endothelial cell
BTA	benzothiazole aniline
BUN	blood urea nitrogen
CAA	cerebral amyloid angiopathy
CBF	cerebral blood flow
CCL2	chemokine (C-C motif) ligand 2
CCR2	chemokine (C-C motif) receptor 2
CeNP	ceria nanoparticle
ChAT	choline acetyltransferase
CMT	carrier-mediated transport
CNS	central nervous system
CPP	cell-penetrating peptide
CRE/CREA	creatinine
CSF	cerebrospinal fluid
CTGF	connective tissue growth factor
DMT	disease-modifying therapy
ECM	extracellular matrix
EGCG	epigallocatechin gallate
EV	extracellular vesicle
5×FAD	five familial Alzheimer's disease
FDA	Food and Drug Administration
FGFR1	Fibroblast growth factor receptor 1
GLUT1	glucose transporter 1
GM1	monosialotetrahexosylganglioside
hFGF21	human fibroblast growth factor 21
HIRMAb	human insulin receptor monoclonal antibody
i.c.m.	intracisterna magna
i.c.v.	intracerebroventricular
i.n.	intranasal
i.p.	intraperitoneal
i.v.	intravenous
ICAM-1	intercellular adhesion molecule-1
IgG	immunoglobulin G
IL	interleukin
InsR	insulin receptor
LAT1	L-type amino acid transporter 1
LDLR	low-density lipoprotein receptor
LfR	lactoferrin receptor
LIPUS	low-intensity pulsed ultrasound
LNP	lipid nanoparticle
LRP-1	low-density lipoprotein receptor-related protein 1
MB	methylene blue
MCI	mild cognitive impairment
miRNA	microRNA
MNP	magnetic nanoparticle
MPS	mononuclear phagocyte system
MRI	magnetic resonance imaging
MSC	mesenchymal stem cell
MSN	mesoporous silica nanoparticle
nAChR	nicotinic acetylcholine receptor
NAD	nicotinamide adenine dinucleotide
NCAM	neural cell adhesion molecule
NFT	neurofibrillary tangle
NGF	nerve growth factor
NIR	near-infrared light irradiation
NLC	nanostructured lipid carrier
NLRP3	NOD-like receptor family pyrin domain-containing 3
NP	nanoparticle
Nrf2	nuclear factor (erythroid-derived 2)-like 2
NSC	neural stem cell
NSE	neuron-specific enolase
NVU	Neurovascular unit
OA	okadaic acid
PAM	plaque-associated microglia
PCL	polycaprolactone
PDA	polydopamine
PdNP	palladium nanoparticle
PEG	poly(ethylene glycol)
PEI	polyethyleneimine
PHFs	paired helical filaments
PLA	polylactic acid
PLGA	poly(lactide-co-glycolide) acid
PMLA	poly(β-L-malic acid-trileucine) copolymer
PROTAC	proteolysis-targeting chimera
PSD95	postsynaptic density protein 95
PTMC	poly(trimethylene carbonate)
PVA	polyvinyl alcohol
PVP	polyvinylpyrrolidone
QD	quantum dot
RAGE	receptor for advanced glycation end-products
RBC	red blood cell
RDP	rabies virus-derived peptide
RES	reticuloendothelial system
rHDL	reconstituted high-density lipoprotein
RMT	receptor-mediated transcytosis
RNS	reactive nitrogen species
ROS	reactive oxygen species
RuNP	ruthenium nanoparticle
RVG	rabies virus glycoprotein
s.c.	subcutaneous
siRNA	small interfering RNA
SLN	solid lipid nanoparticle
SPION	superparamagnetic iron oxide nanoparticle
STZ	streptozocin
SYP	synaptophysin
TAT	trans-activator of transcription
TC	total cholesterol
TfR	transferrin receptor
3×Tg	triple transgenic
TJ	tight junction
TNF-α	tumor necrosis factor-α
TP	total protein
TREM2	triggering receptor expressed on myeloid cells 2
VCAM-1	vascular cell adhesion molecule-1
VLA-4	very late antigen-4
WBC	white blood cell
ZO	zonula occludens

## Declaration of competing interest

The authors declare that they have no known competing financial interests or personal relationships that could have appeared to influence the work reported in this paper.

## Data Availability

No data was used for the research described in the article.
